# Supplementary material for: Endogenous retroviruses mediate transcriptional rewiring in response to oncogenic signaling in colorectal cancer
Source: Sci Adv. 2024 Jul 17;10(29):eado1218. doi: 10.1126/sciadv.ado1218 (PMC466953; doi:10.1126/sciadv.ado1218)
Supplement: Supplementary file 1 — Figs. S1 to S84 Legends for tables S1 to S27 [file sciadv.ado1218_sm.pdf]

Science Advances
AAAS

Supplementary Materials for

Endogenous retroviruses mediate transcriptional rewiring in response to oncogenic signaling in colorectal cancer

Atma Ivancevic *et al.*

Corresponding author: Edward B. Chuong, edward.chuong@colorado.edu

Sci. Adv. **10**, eado1218 (2024)
DOI: 10.1126/sciadv.ado1218

The PDF file includes:

Figs. S1 to S84
Legends for tables S1 to S27

Other Supplementary Material for this manuscript includes the following:

Tables S1 to S27

Fig. S1.

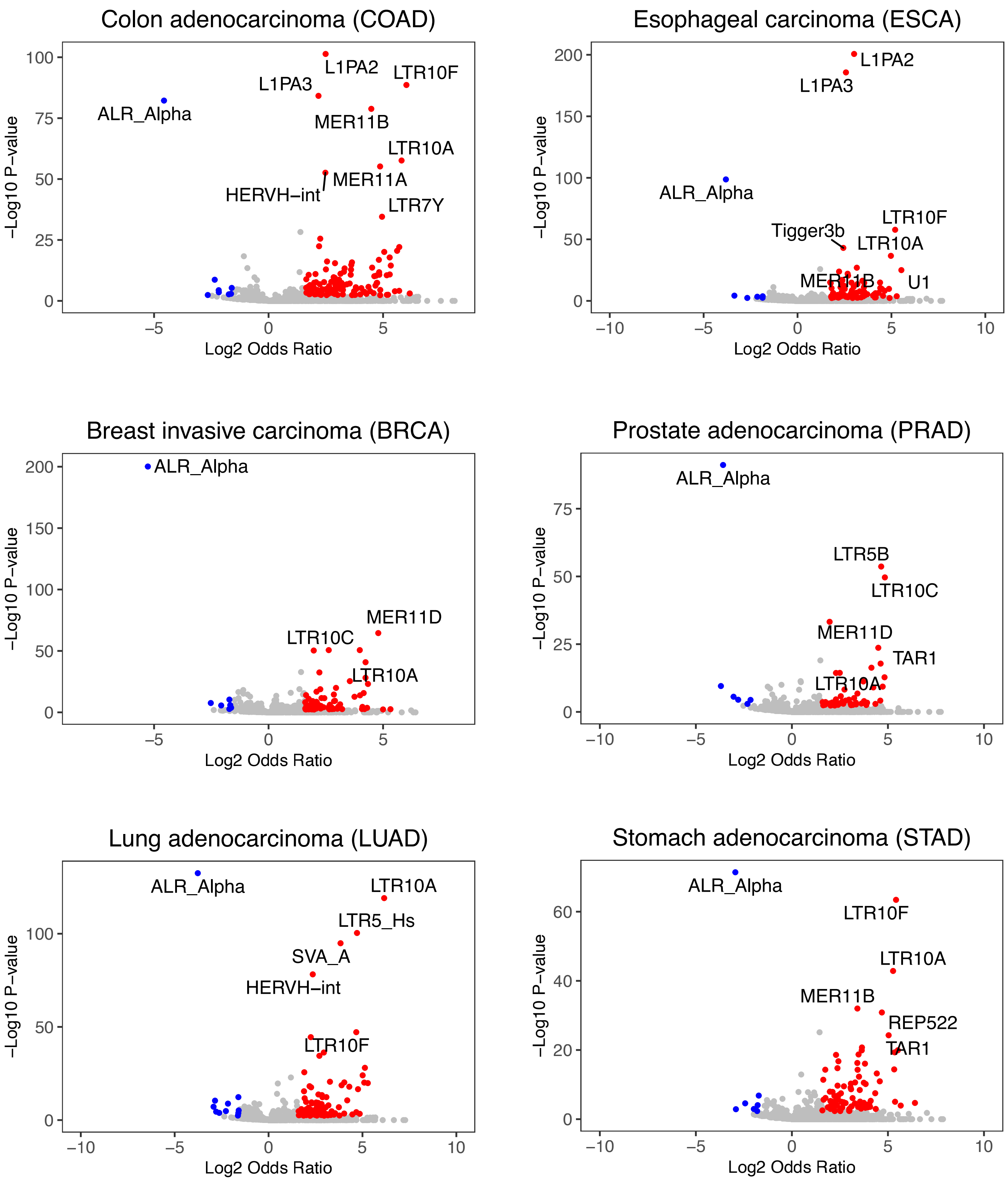

Fig. S1. Enrichment of TE families within cancer-specific ATAC-seq associated with different cancer subtypes from TCGA. Significantly enriched TEs are shown in red; depleted TEs are shown in blue.

Fig. S2.

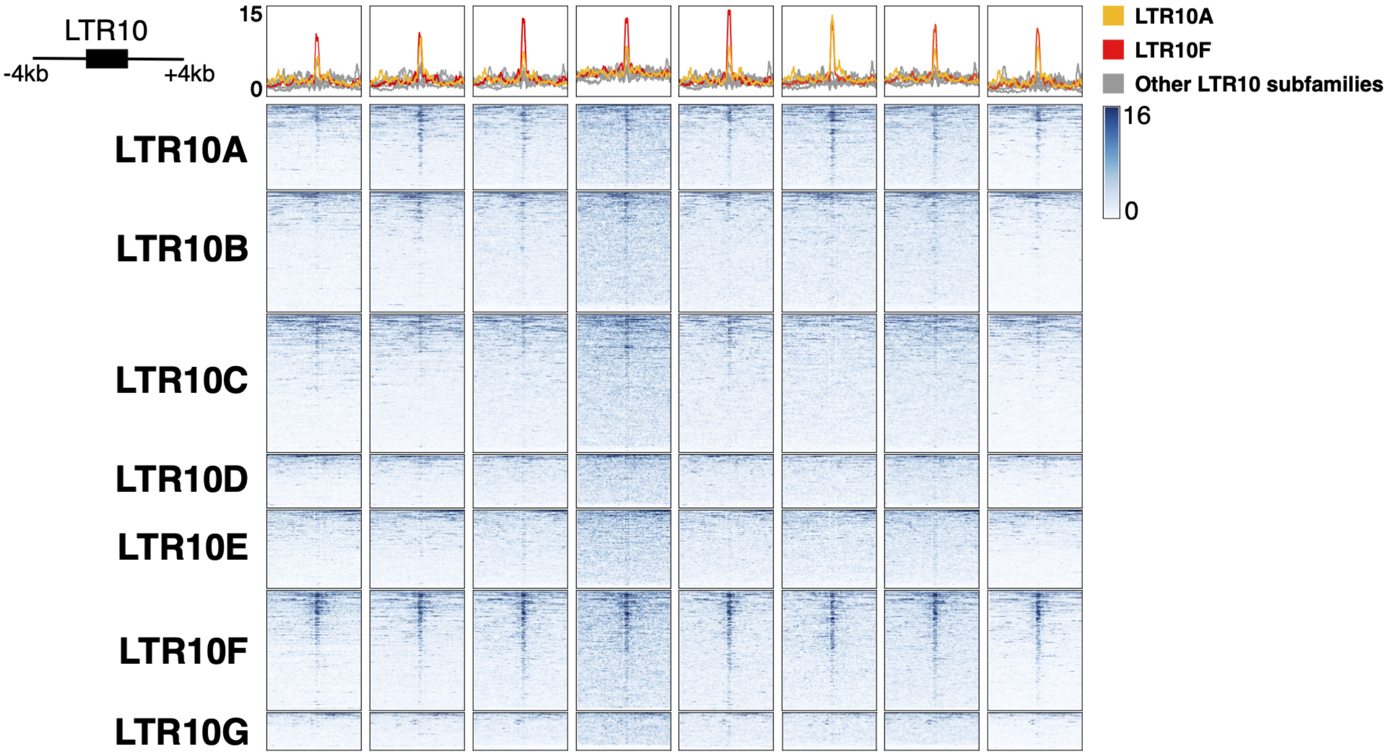

Fig. S2. Heatmap of representative patient tumor ATAC-seq signals (TCGA patients P053, P012, P002, P025, P004, P016, P001, P049) over all LTR10 elements, separated by LTR10 subfamily. Metaprofiles represent average normalized ATAC signal across elements.

Fig. S3.

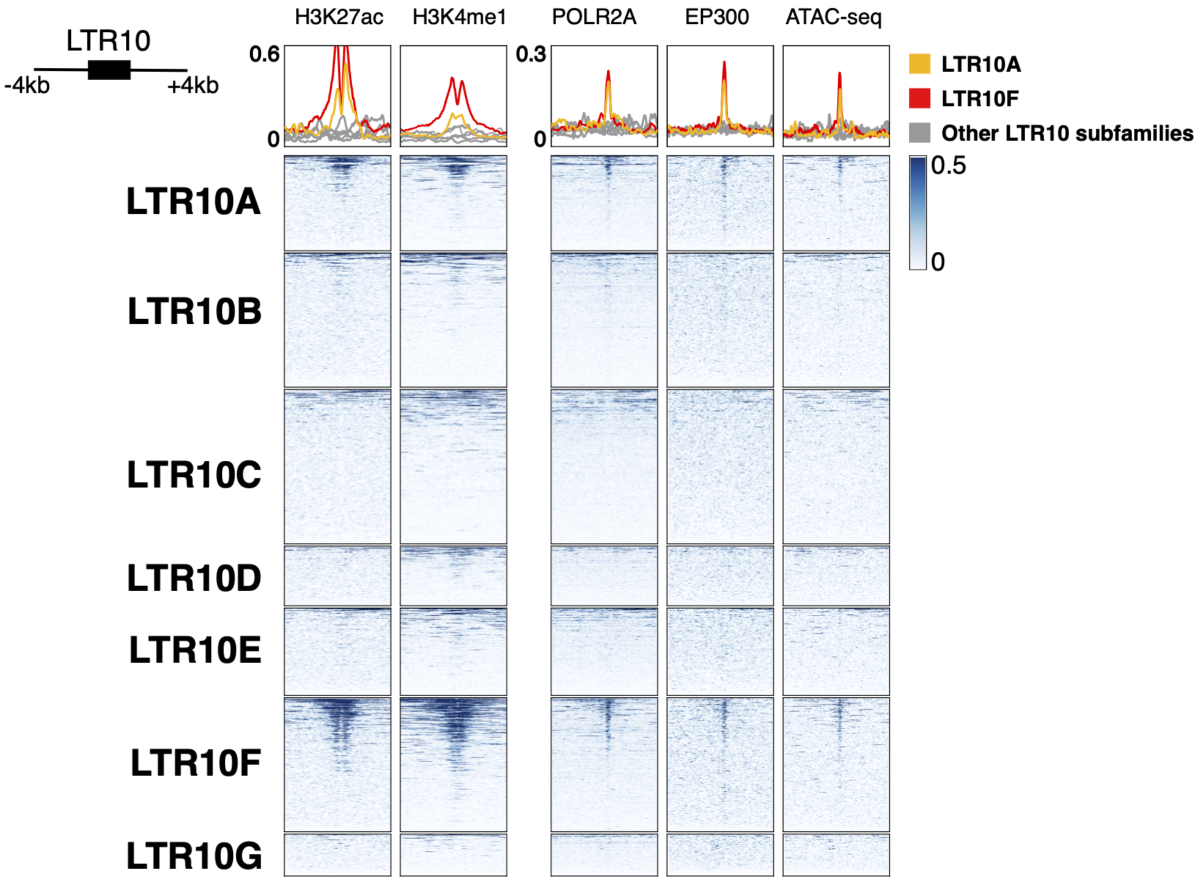

Fig. S3. Heatmap of enhancer-associated chromatin marks from HCT116 cells over all LTR10 elements, separated by LTR10 subfamily. From left to right: H3K27ac ChIP-seq (GSE97527), H3K4me1 ChIP-seq (GSE101646), POLR2A ChIP-seq (GSE32465), EP300 ChIP-seq (GSE51176), and HCT116 ATAC-seq (GSE126215). Metaprofiles represent the normalized signal across elements.

Fig. S4.

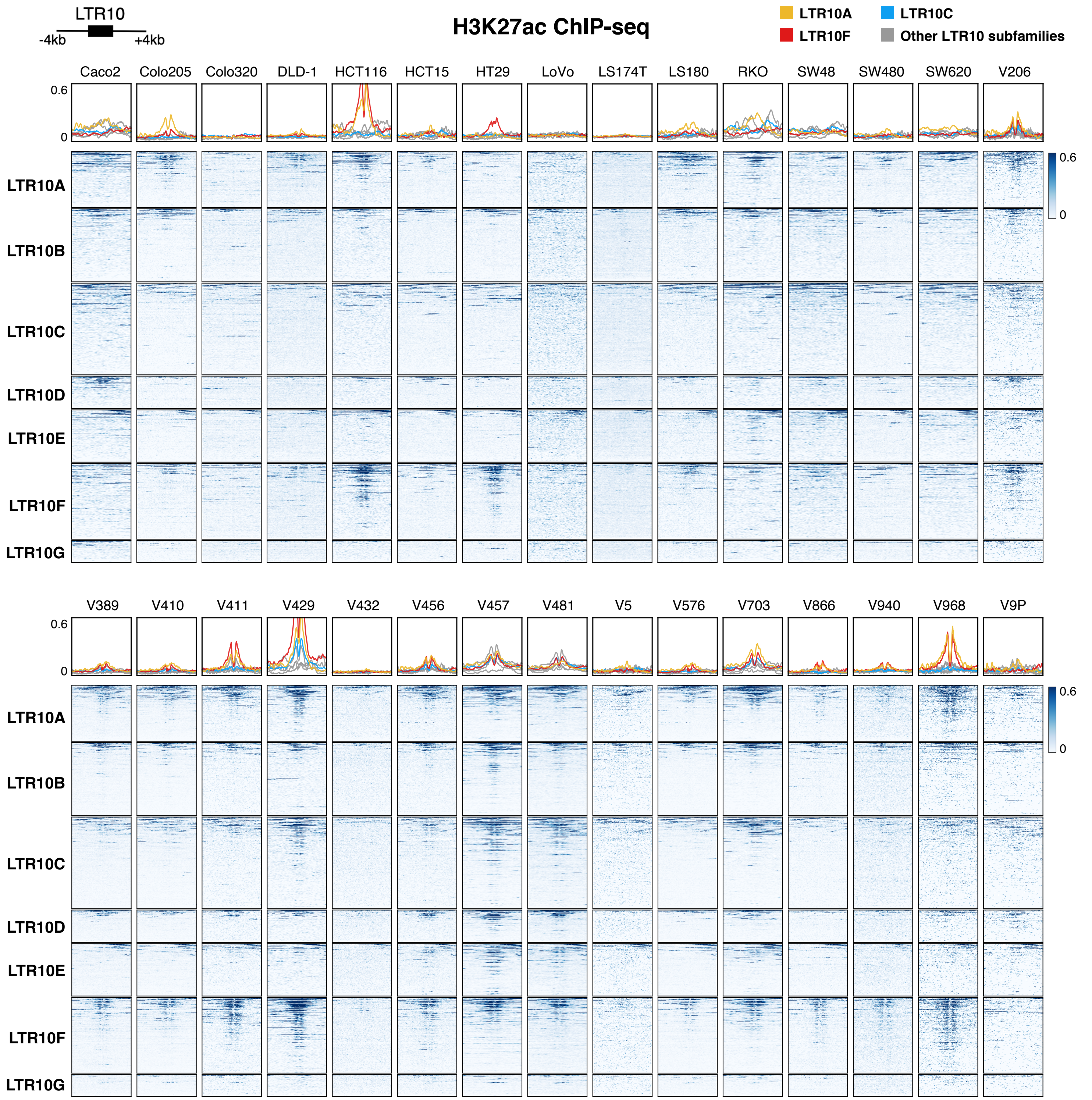

Fig. S4. Heatmap of H3K27ac ChIP-seq from 30 colorectal cancer cell lines over all LTR10 elements, separated by LTR10 subfamily. Cell line mutation information and GEO accession numbers are provided in table S2. Metaprofiles represent the normalized signal across elements.

Fig. S5.

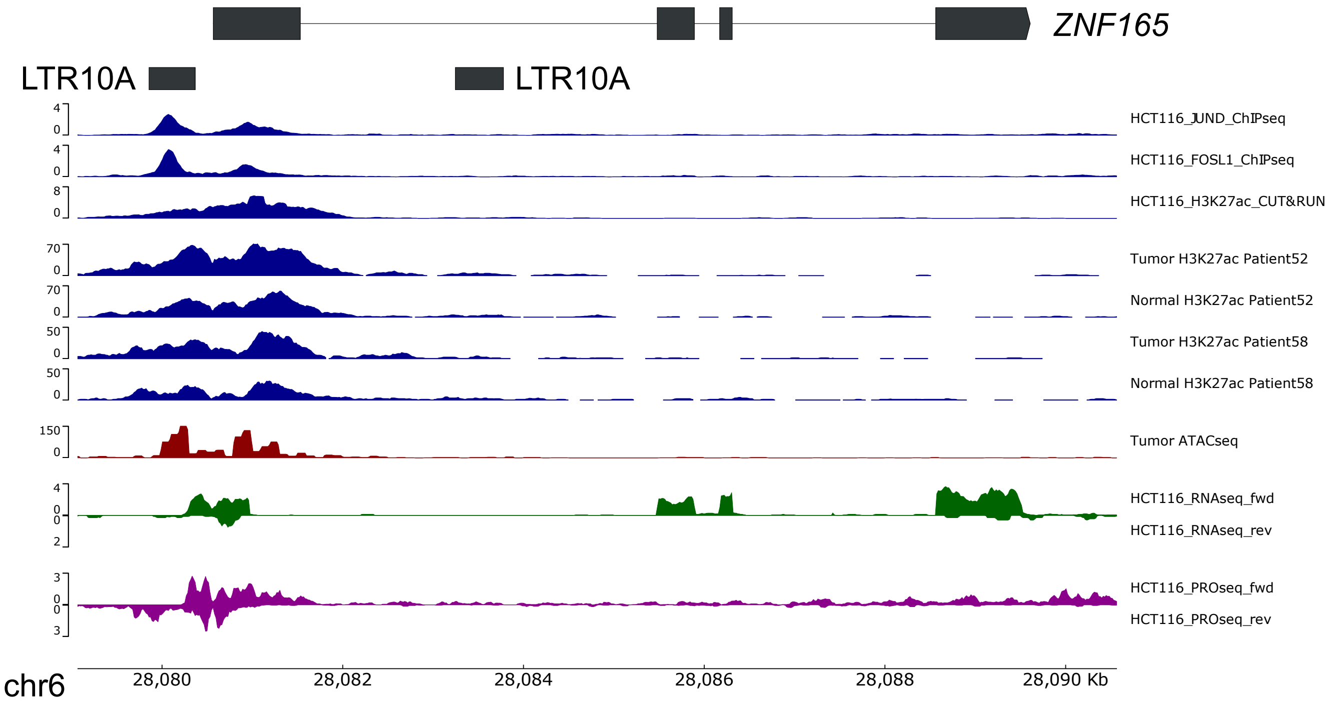

Fig. S5. Genome browser view of an LTR10A element co-opted as a promoter for *ZNF165*. From top to bottom: JUND and FOSL1 ChIP-seq (GSE32465), H3K27ac CUT&RUN (in-house), H3K27ac ChIP-seq from matched tumor/normal samples from the CEMT Canadian Epigenome Project (patients AKCC52 and AKCC58), tumor ATAC-seq from TCGA (patient COAD P022), HCT116 RNA-seq (in-house), and HCT116 PRO-seq (GSE129501).

Fig. S6.

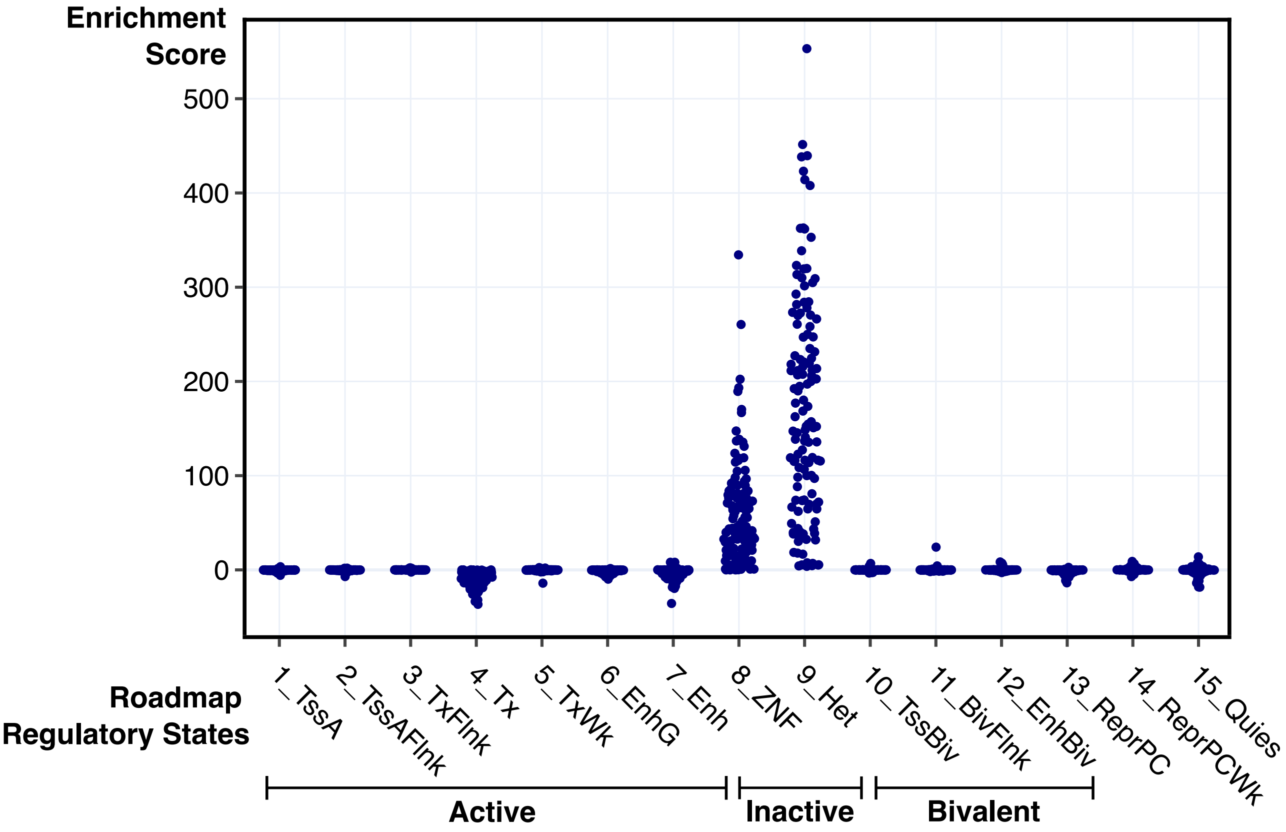

Fig. S6. Dotplot showing enrichment scores of LTR10A/F elements for all Roadmap tissues (n=127), across the fifteen regulatory states defined by Roadmap.

Fig. S7.

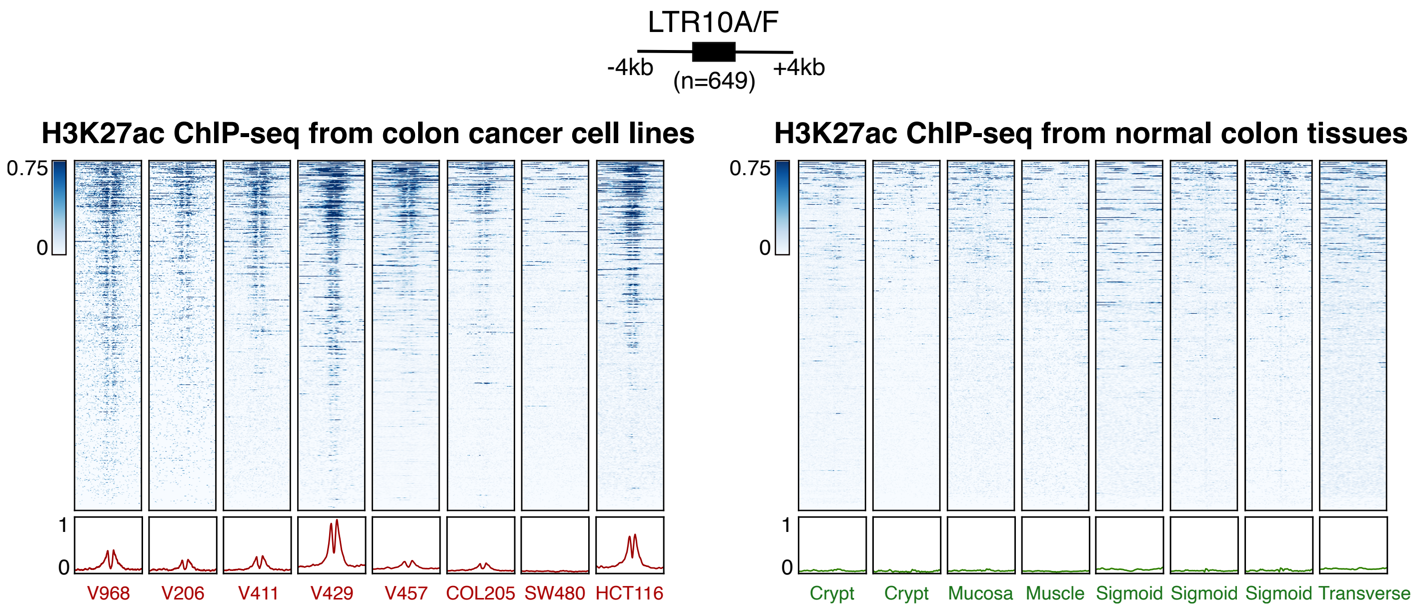

Fig. S7. Heatmap of H3K27ac ChIP-seq from different colorectal cancer cell lines (GSE77737) versus H3K27ac ChIP-seq from normal colon tissues (GSE77737, GSE17312, GSE101136, GSE101031, GSE16256), over the merged set of 649 LTR10A/F elements. Bottom metaprofiles represent the normalized signal across elements.

Fig. S8.

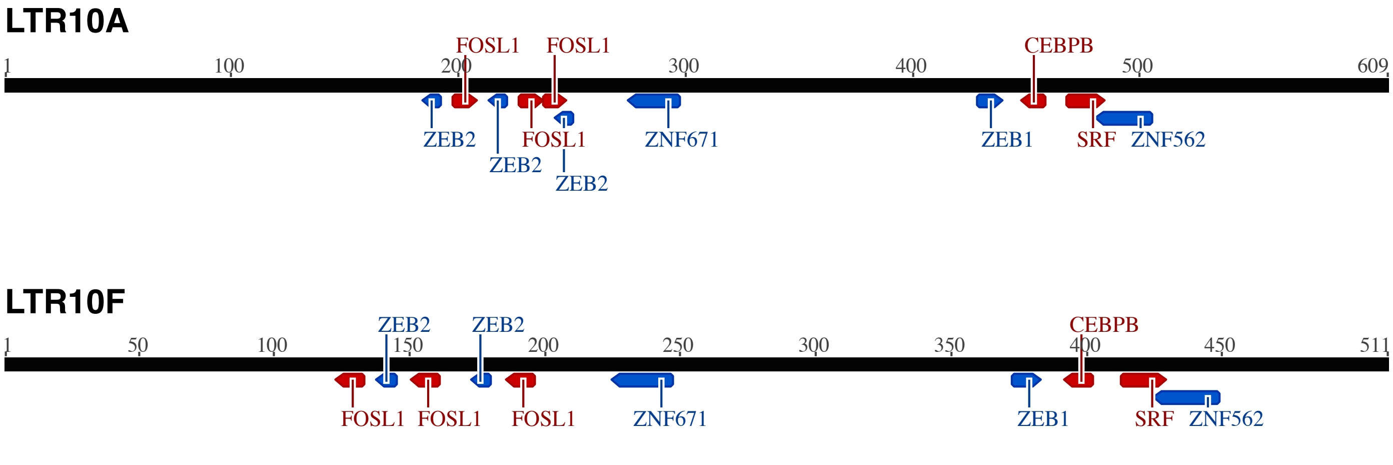

Fig. S8. Schematic of predicted activator and repressor binding motifs within LTR10A and LTR10F consensus sequences. Transcriptional activators are colored in red; repressors are colored in blue.

Fig. S9.

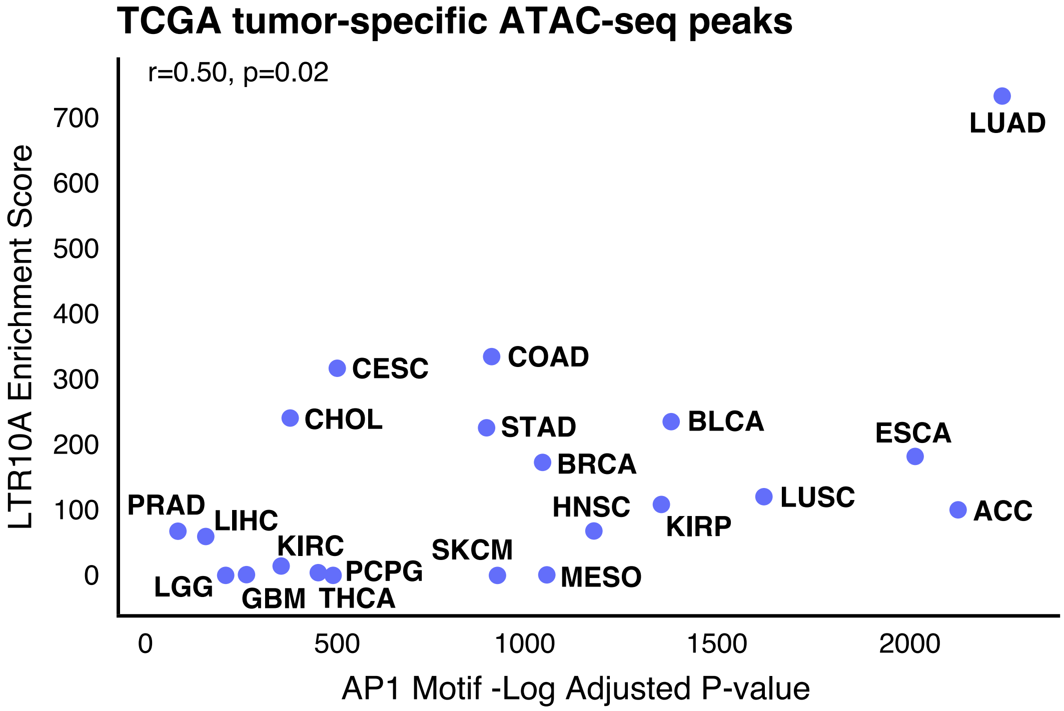

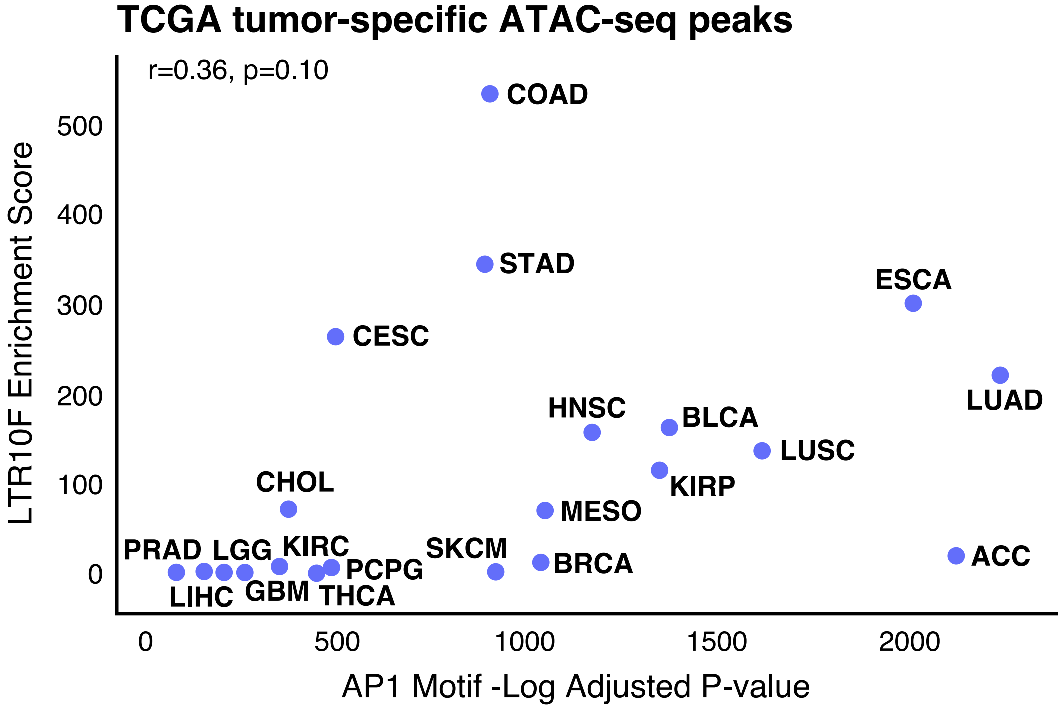

Fig. S9. Enrichment of LTR10A or LTR10F versus enrichment of AP1 binding motifs in tumor-specific ATAC-seq peaks for 21 cancer types. Cancer types are labeled by their TCGA abbreviations (e.g. COAD = colon adenocarcinoma). The Pearson correlation coefficient (r) and p-value are shown for each plot.

Fig. S10.

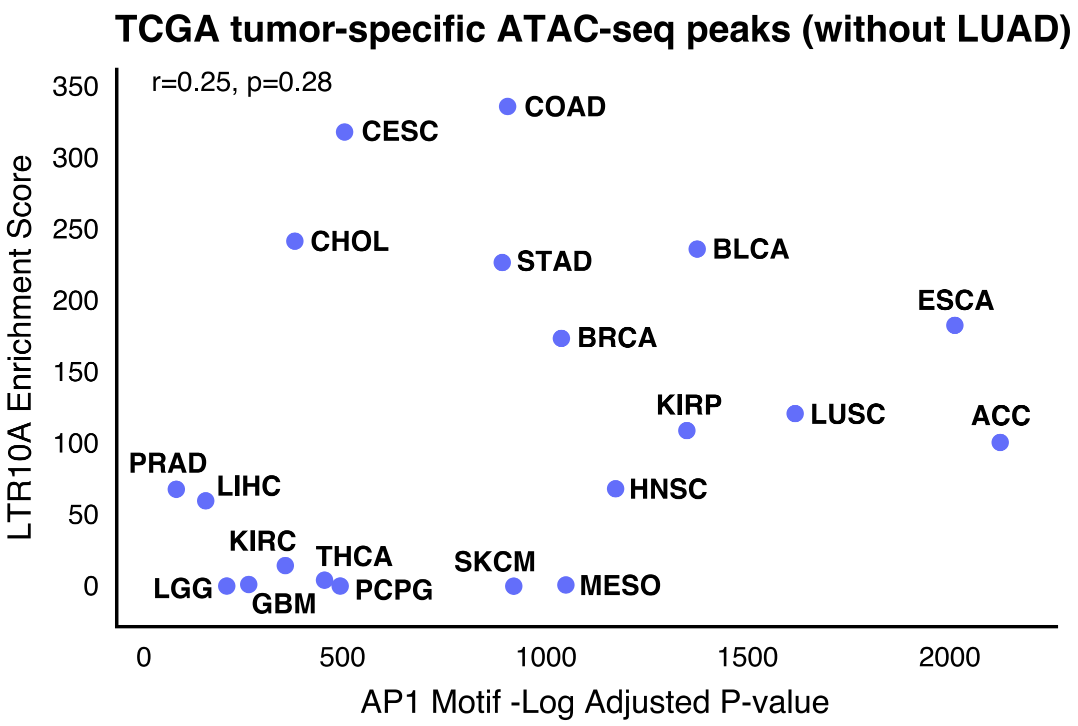

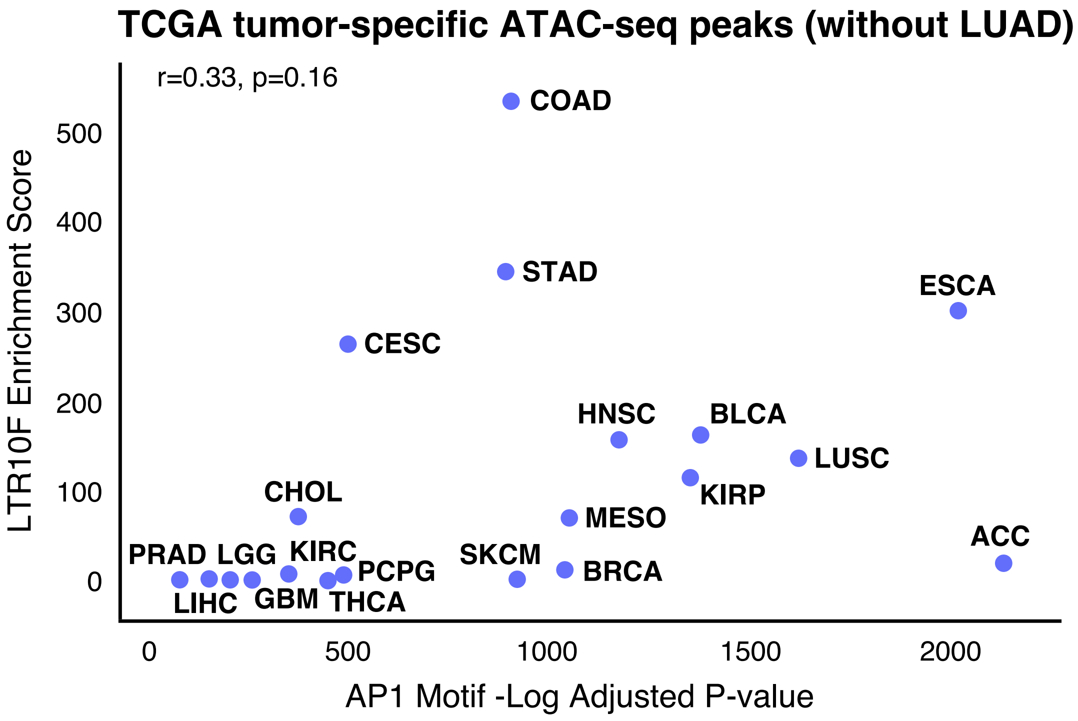

Fig. S10. Same as fig. S9, but with lung adenocarcinomas (LUAD) removed as an outlier. Enrichment of LTR10A or LTR10F versus enrichment of AP1 binding motifs in tumor-specific ATAC-seq peaks for 20 cancer types. Cancer types are labeled by their TCGA abbreviations (e.g. COAD = colon adenocarcinoma). The Pearson correlation coefficient (r) and p-value are shown for each plot.

Fig. S11.

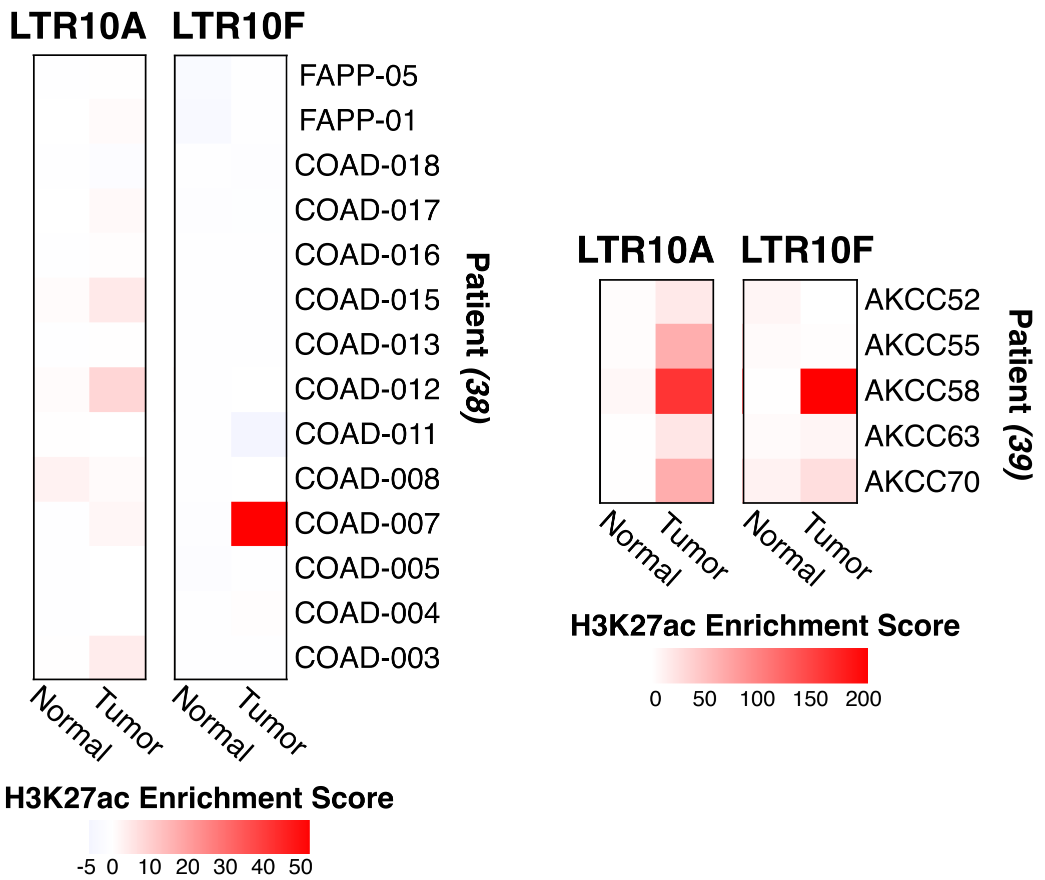

Fig. S11. Enrichment of LTR10A/F elements in H3K27ac-defined regulatory regions from patient matched tumor/normal samples. ChIP-Seq data obtained from *(38)* and *(39)*.

Fig. S12.

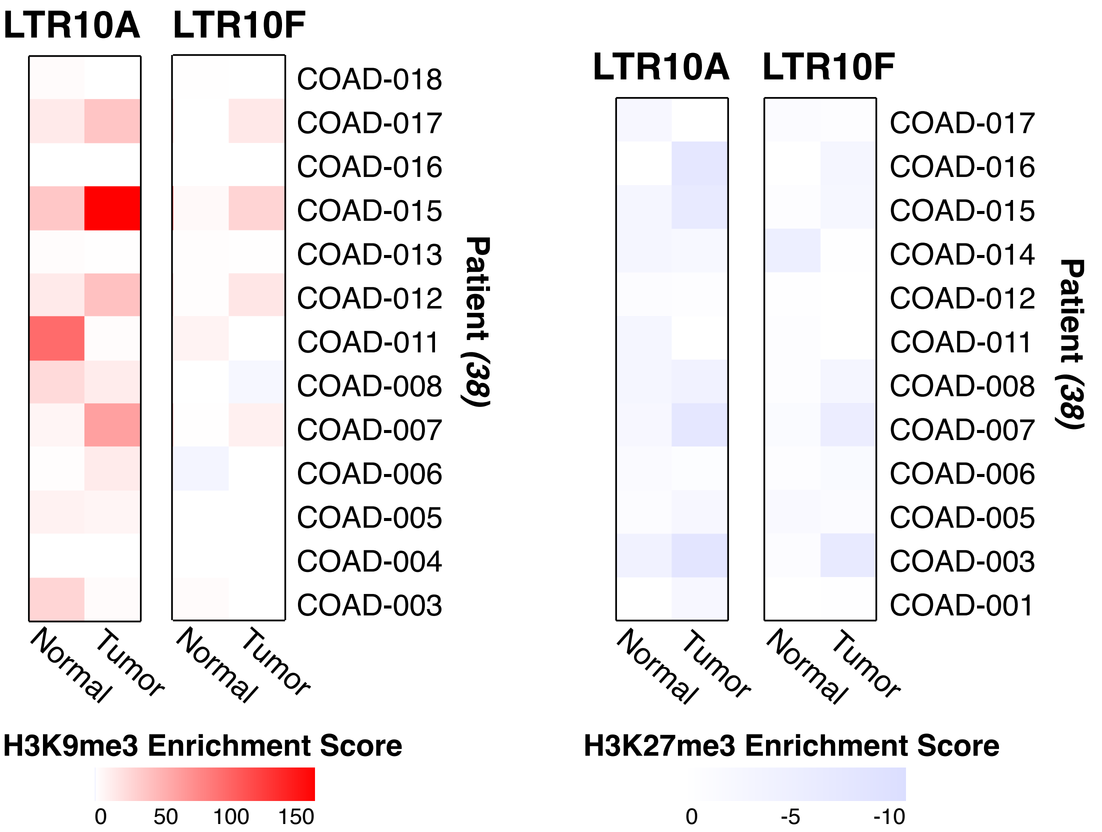

Fig. S12. Enrichment of LTR10A/F elements in H3K9me3 and H3K27me3 regulatory regions from patient matched tumor/normal samples. ChIP-Seq data obtained from *(38)*.

Fig. S13.

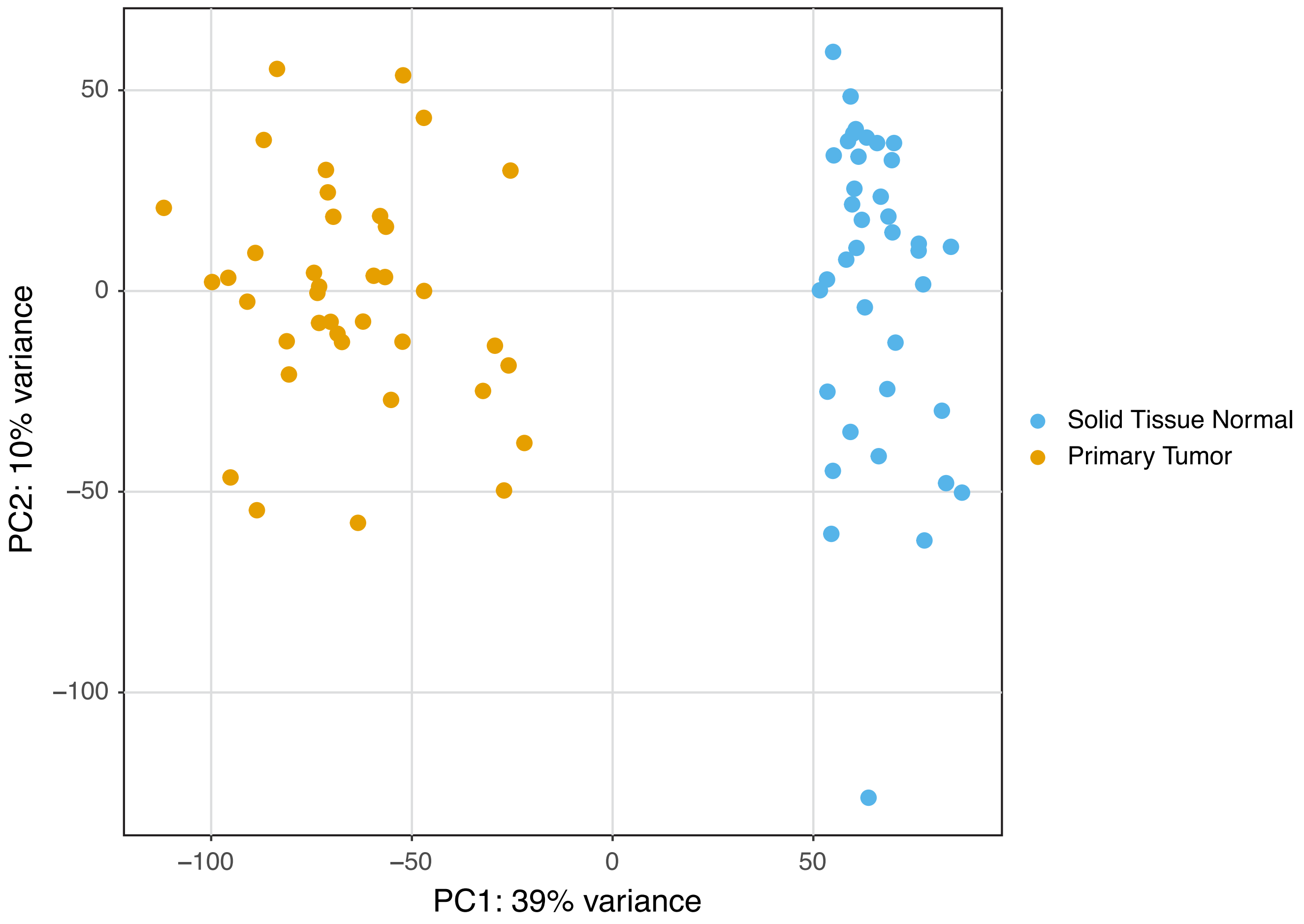

Fig. S13. Principal component analysis (PCA) of bulk RNA-seq data from TCGA-COAD controlled access data, showing matched tumor/normal samples from 38 patients with colorectal adenocarcinomas. Each patient has one tumor and one normal colon sample. PCA is based on gene expression only (TEs not included).

Fig. S14.

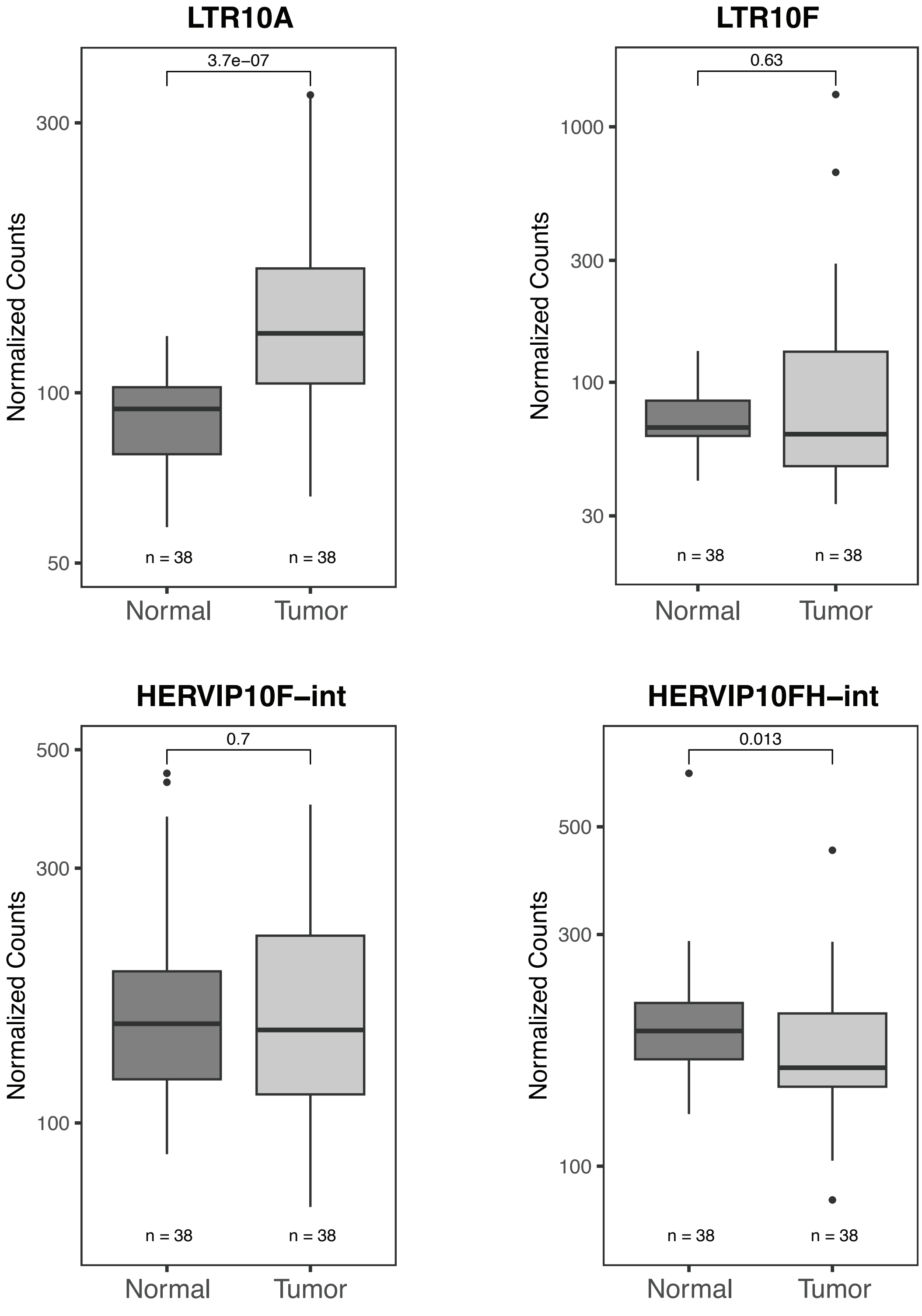

Fig. S14. Boxplots of paired sample Wilcoxon test p-values for normalized counts of LTR10 transcripts from bulk RNA-seq of 38 patient-derived matched tumor/normal samples. Normalized counts are depicted on a log scale. RNA-seq was downloaded from TCGA-COAD controlled access data.

Fig. S15.

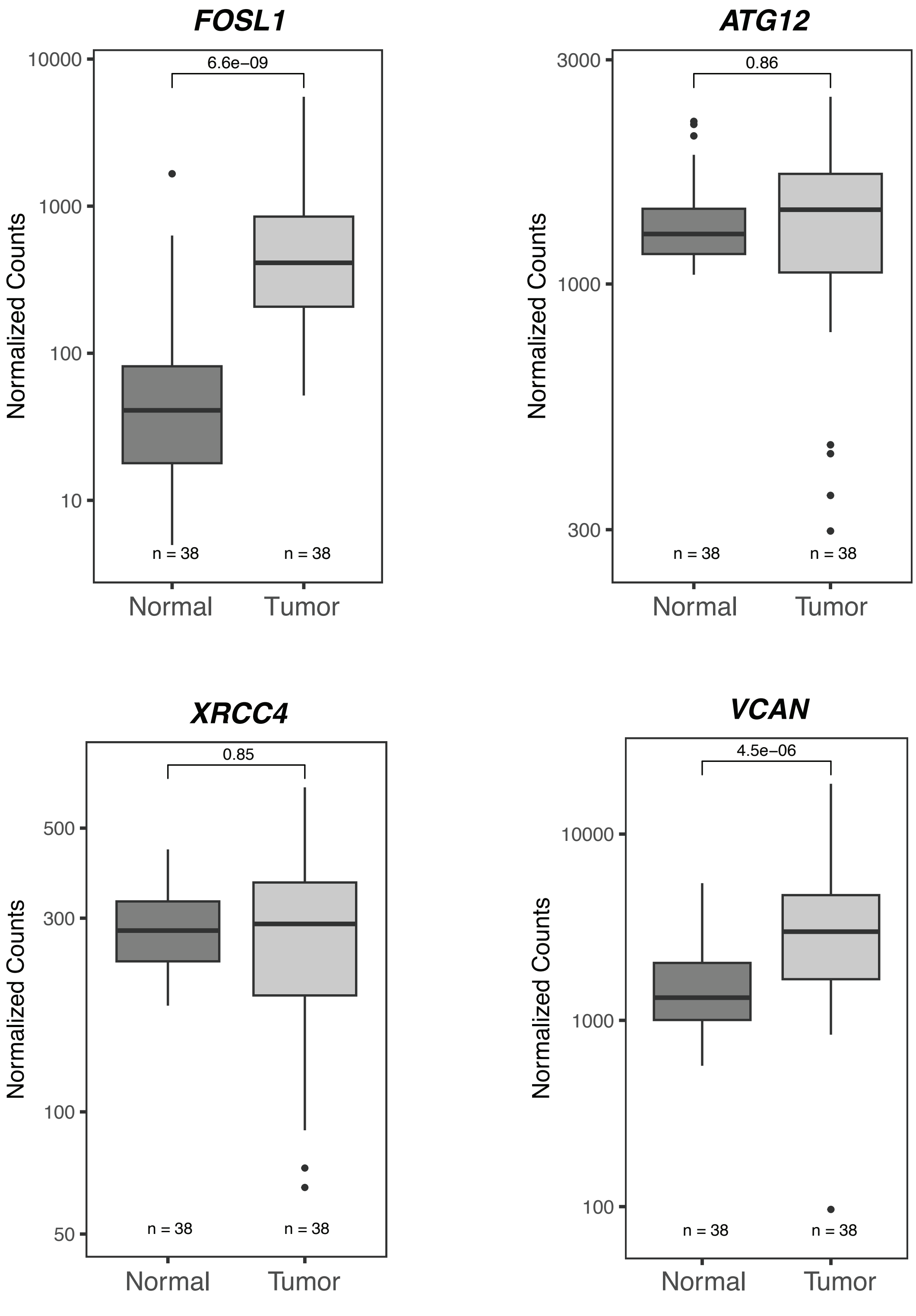

Fig. S15. Boxplots of paired sample Wilcoxon test p-values for normalized counts of LTR10-associated genes from bulk RNA-seq of 38 patient-derived matched tumor/normal samples. Normalized counts are depicted on a log scale. RNA-seq was downloaded from TCGA-COAD controlled access data.

Fig. S16.

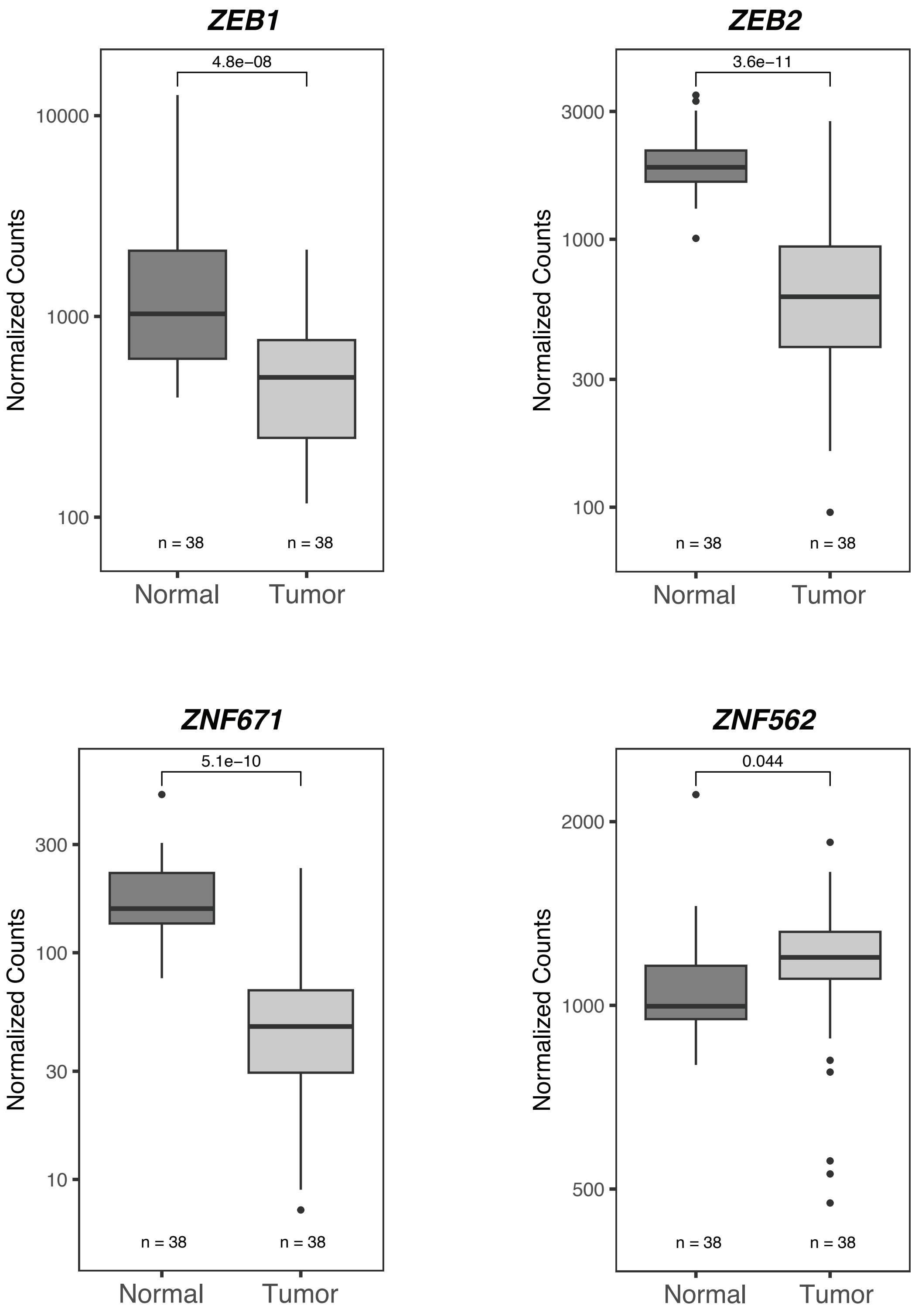

Fig. S16. Boxplots of paired sample Wilcoxon test p-values for normalized counts of zinc fingers predicted to act as LTR10 repressors, from bulk RNA-seq of 38 patient-derived matched tumor/normal samples. Note that *ZNF562* is a predicted repressor of LTR10 elements in healthy cells, but appears to be regulated by an LTR10F promoter/enhancer in colorectal cancer cells. Normalized counts are depicted on a log scale. RNA-seq was downloaded from TCGA-COAD controlled access data.

Fig. S17.

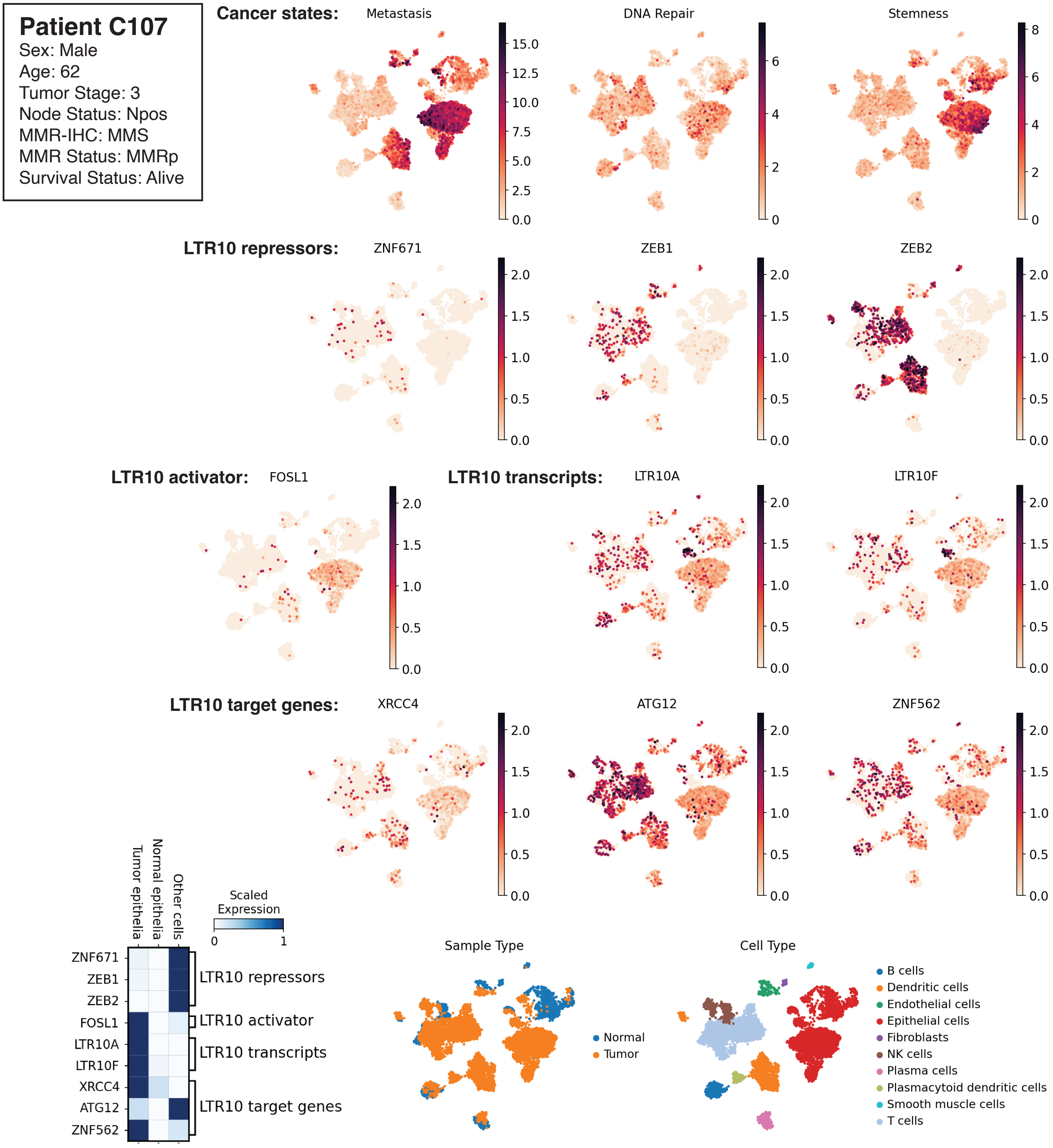

Fig. S17. Detailed analysis of patient C107 using single cell RNA-seq from *(40)*. Cancer states were predicted using the CancerSEA database. Cell types were inferred using the PanglaoDB database of gene expression markers. Sample type was provided by the original authors.

Fig. S18.

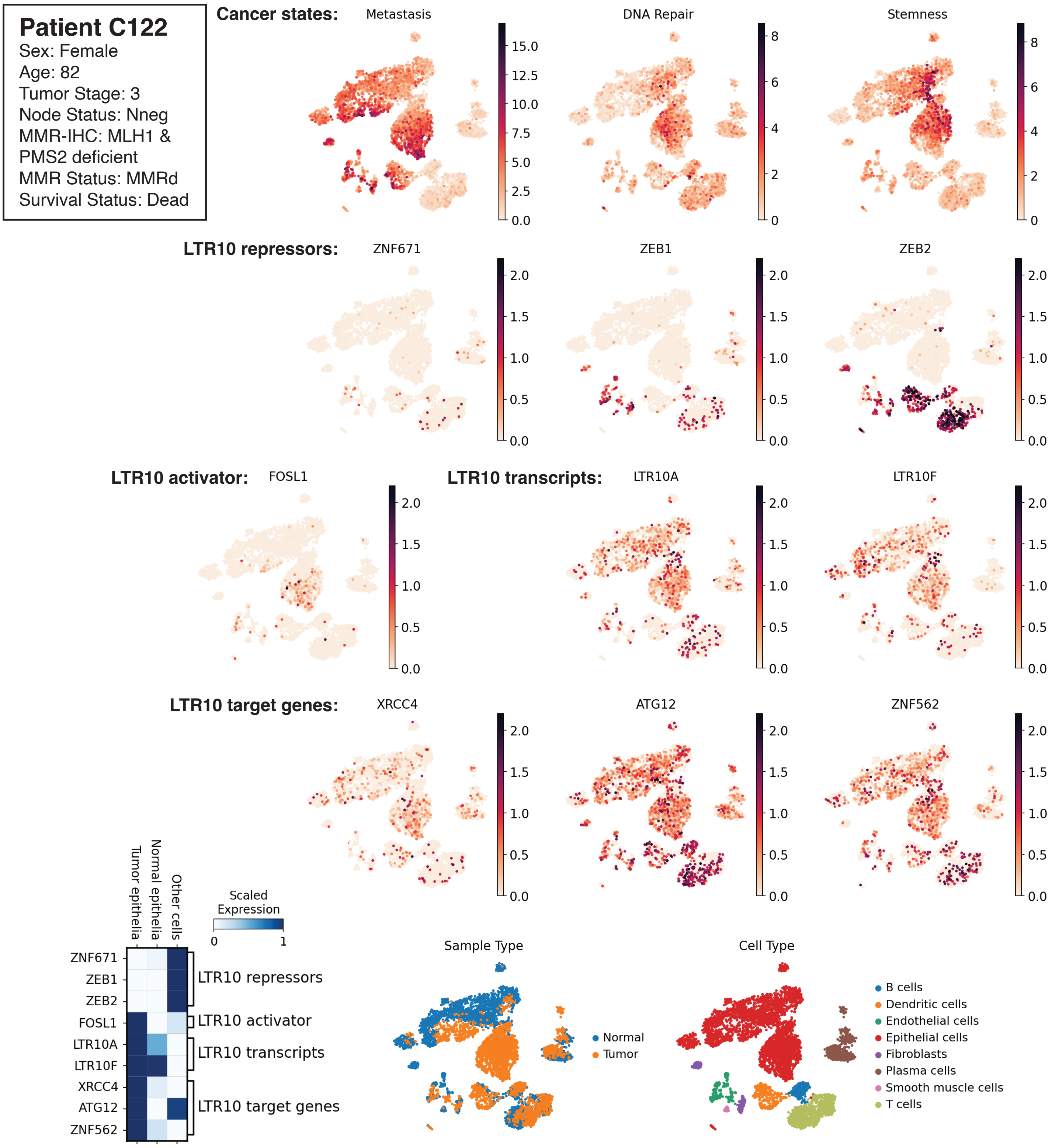

Fig. S18. Detailed analysis of patient C122 using single cell RNA-seq from *(40)*. Cancer states were predicted using the CancerSEA database. Cell types were inferred using the PanglaoDB database of gene expression markers. Sample type was provided by the original authors.

Fig. S19.

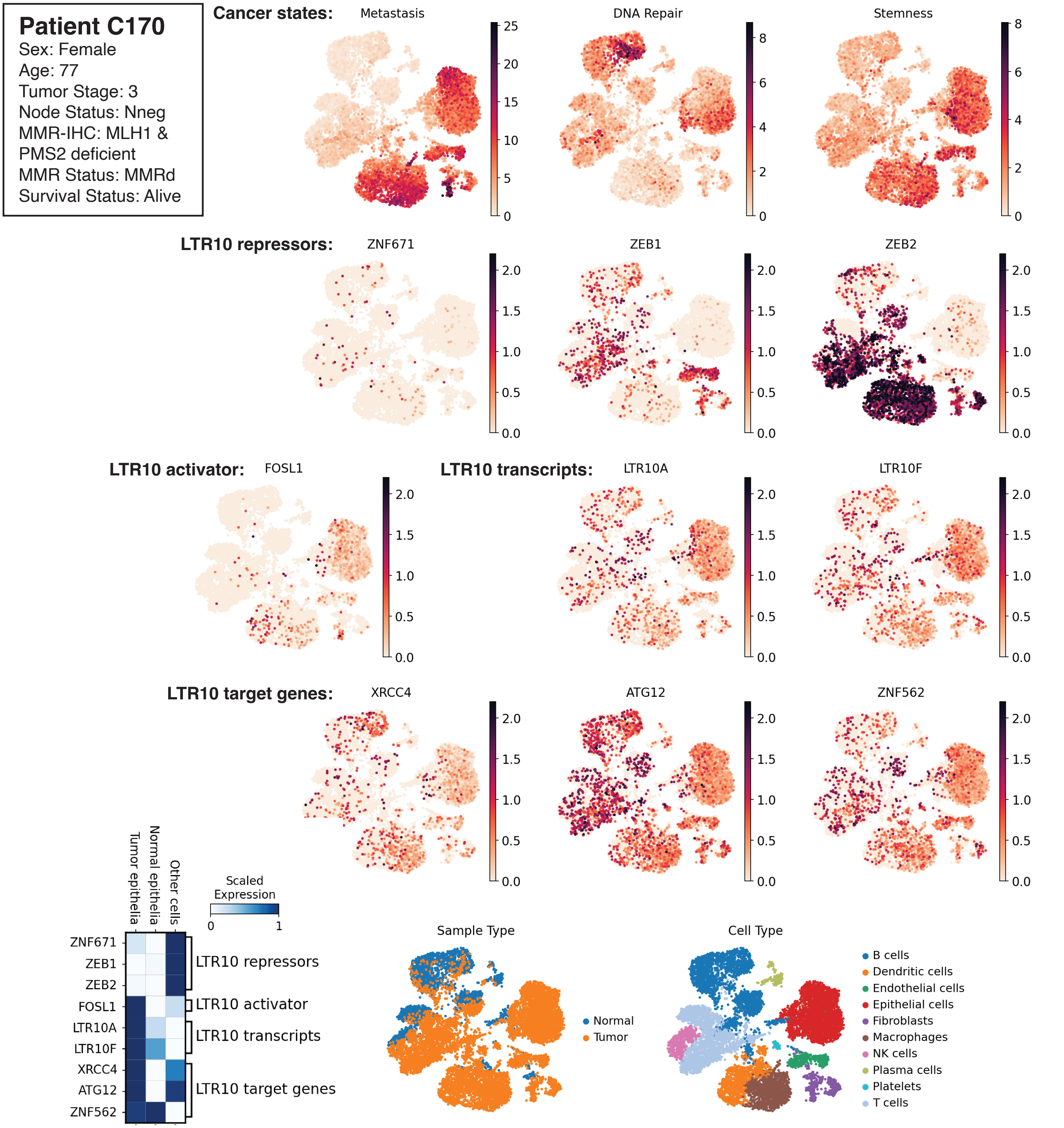

Fig. S19. Detailed analysis of patient C170 using single cell RNA-seq from *(40)*. Cancer states were predicted using the CancerSEA database. Cell types were inferred using the PanglaoDB database of gene expression markers. Sample type was provided by the original authors.

Fig. S20.

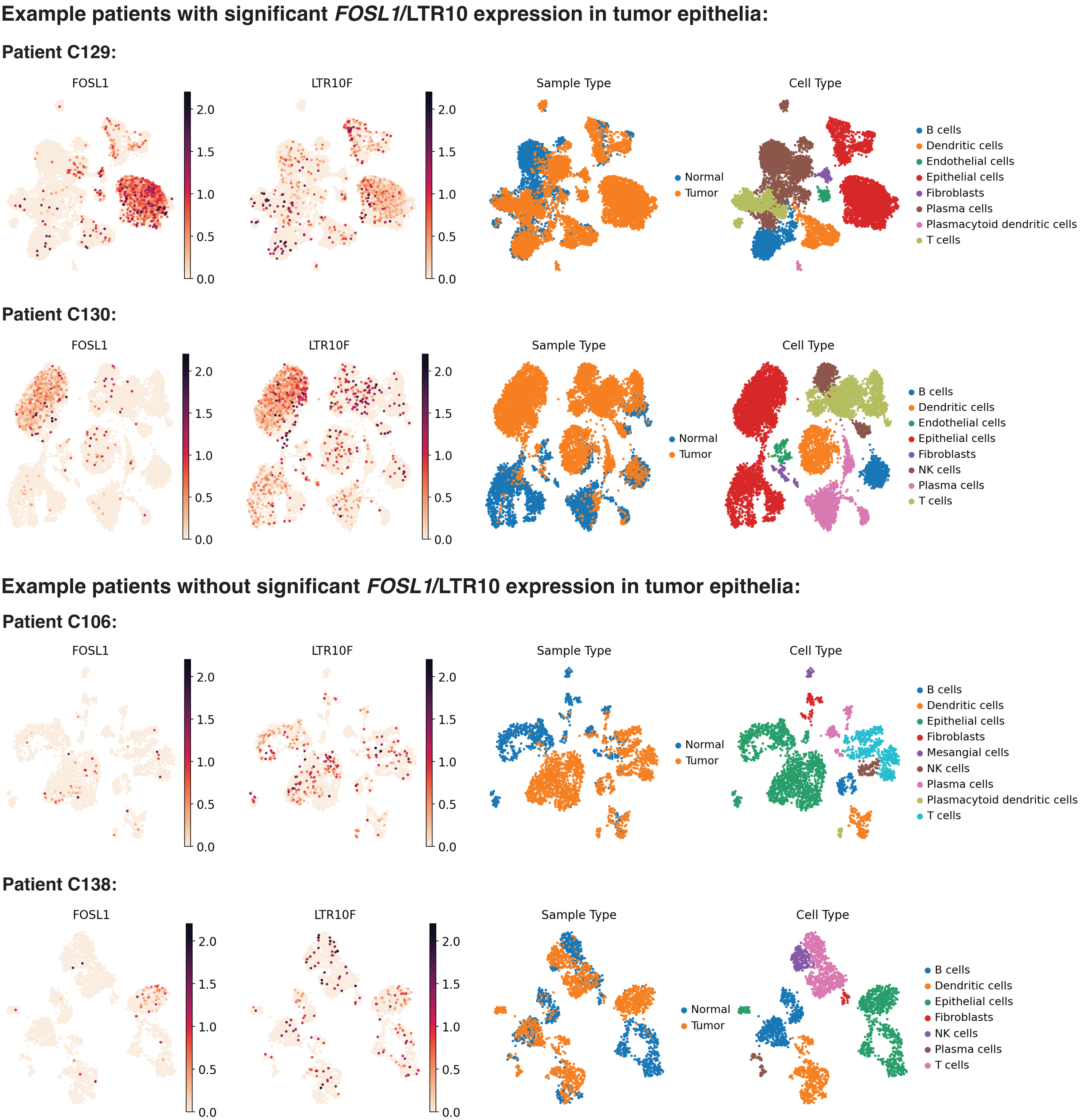

Fig. S20. UMAP visualization of single cell RNA-seq showing *FOSL1* and LTR10F expression profiles for additional patients from *(40)*.

Fig. S21.

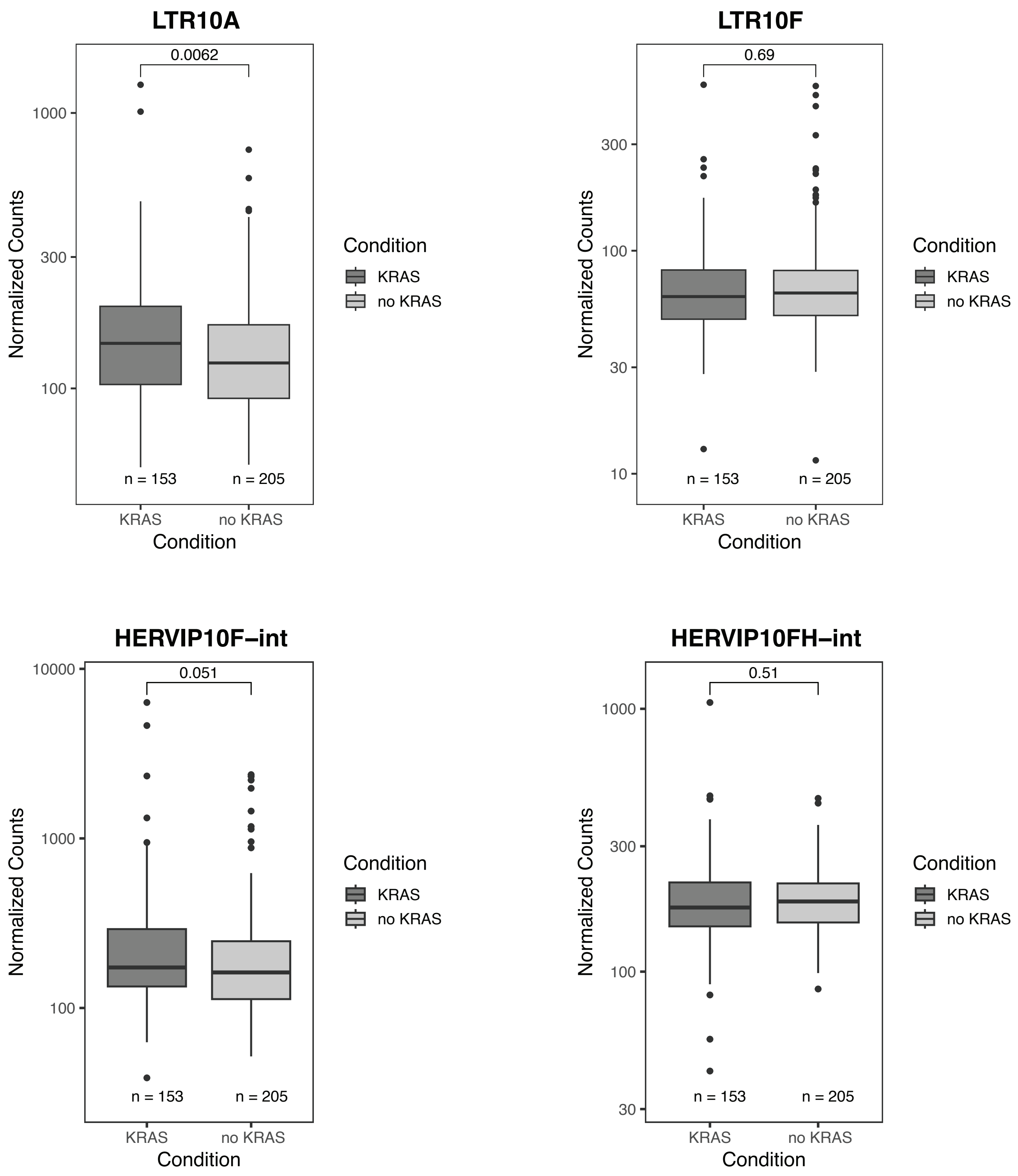

Fig. S21. Boxplots of Wilcoxon test p-values (unpaired) for normalized counts of LTR10 transcripts from bulk RNA-seq of 358 colorectal tumors with *KRAS* mutation status. KRAS = tumor with *KRAS* mutation; no KRAS = tumor without *KRAS* mutation. Normalized counts are depicted on a log scale. Tumor RNA-seq was downloaded from TCGA-COAD controlled access data.

Fig. S22.

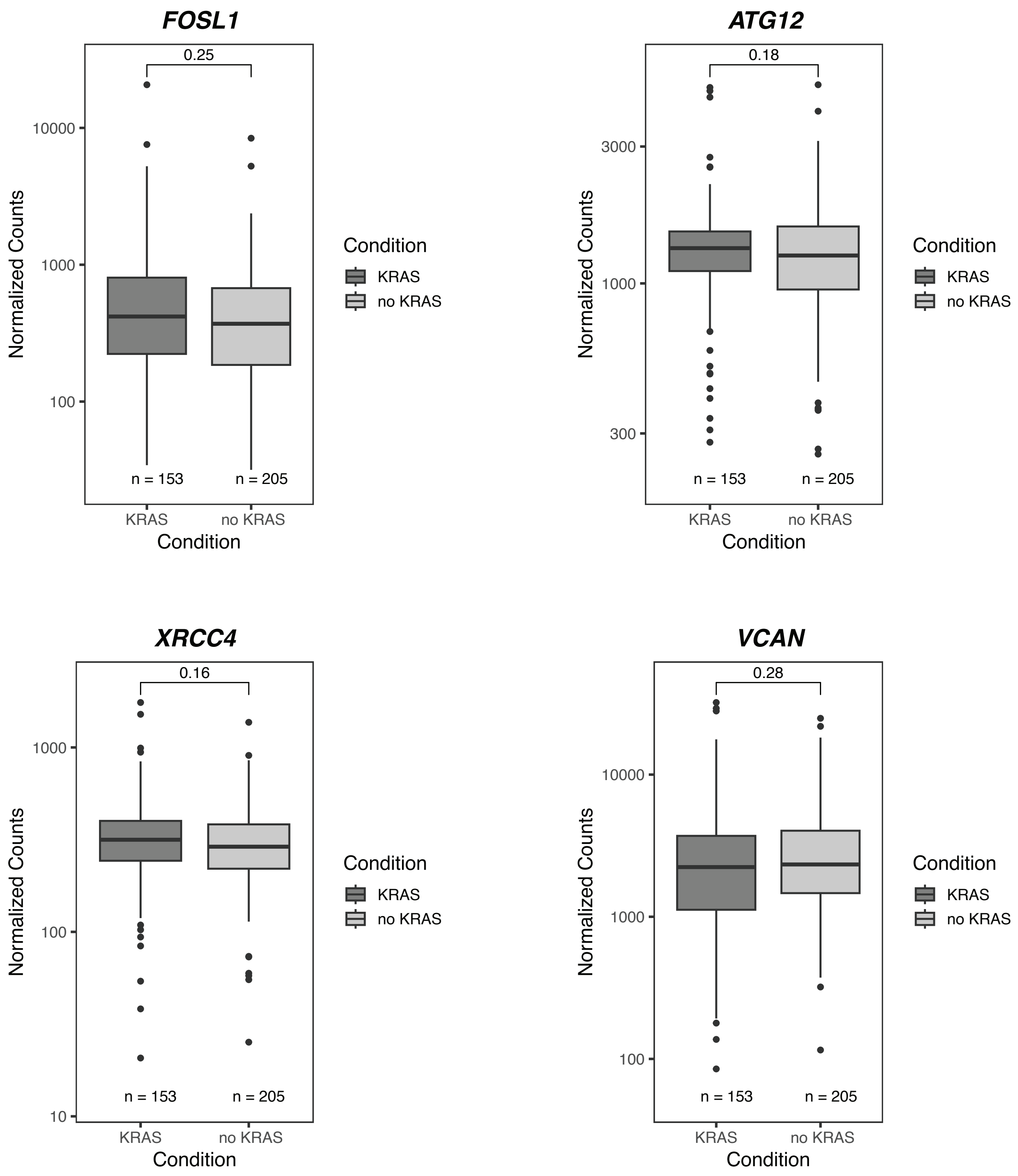

Fig. S22. Boxplots of Wilcoxon test p-values (unpaired) for normalized counts of LTR10-associated genes from bulk RNA-seq of 358 colorectal tumors with *KRAS* mutation status. KRAS = tumor with *KRAS* mutation; no KRAS = tumor without *KRAS* mutation. Normalized counts are depicted on a log scale. Tumor RNA-seq was downloaded from TCGA-COAD controlled access data.

Fig. S23.

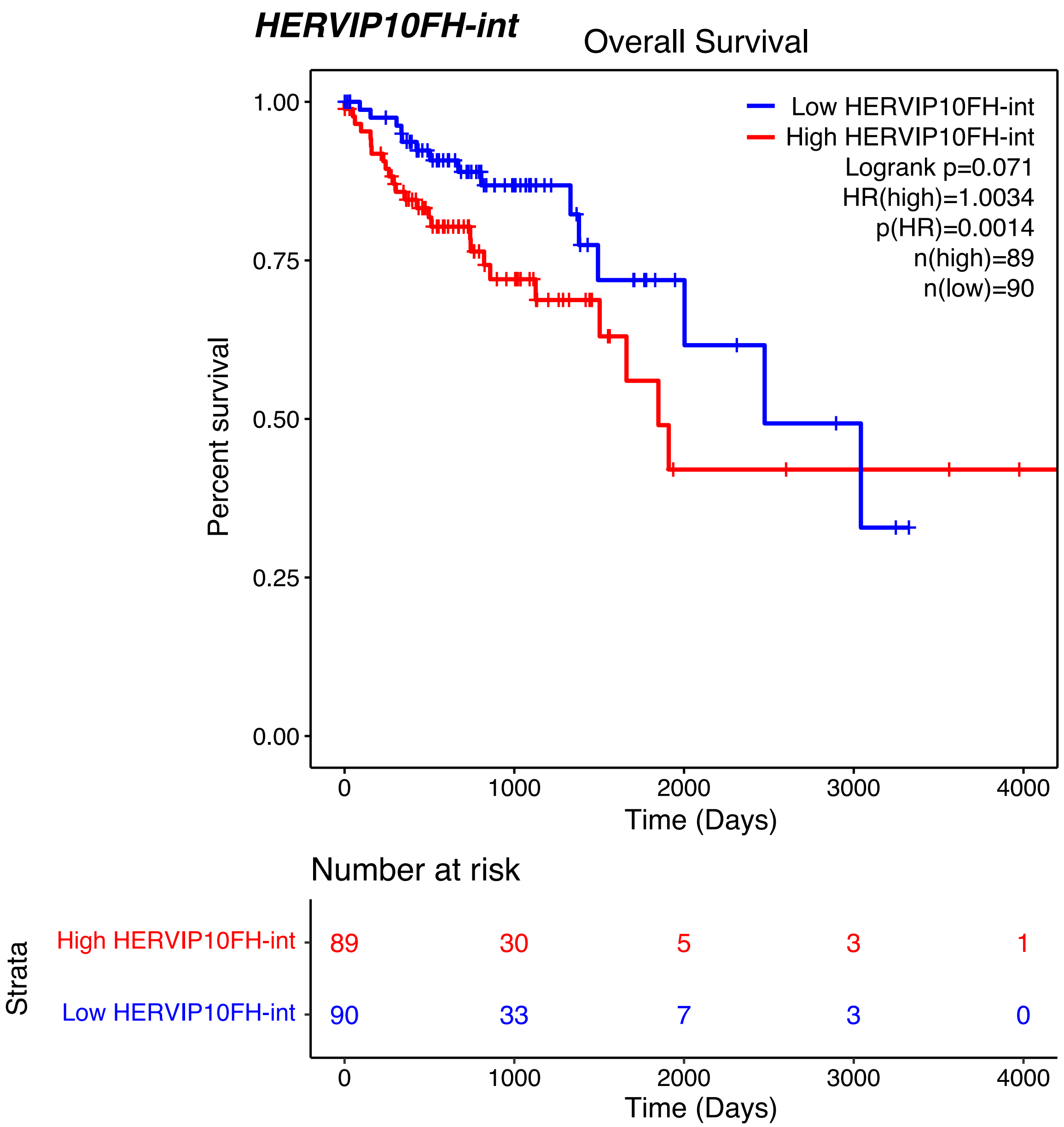

Fig. S23. Kaplan-Meier and Cox regression survival analyses evaluating the prognostic significance of HERVIP10FH-int expression in colorectal cancer. A total of 358 colorectal tumors from the TCGA-COAD dataset were analyzed based on normalized HERVIP10FH-int expression levels. Patients were categorized into groups according to the upper and lower quartiles of HERVIP10FH-int expression, and survival rates between the groups were compared to assess the impact of HERVIP10FH-int on patient outcomes.

Fig. S24.

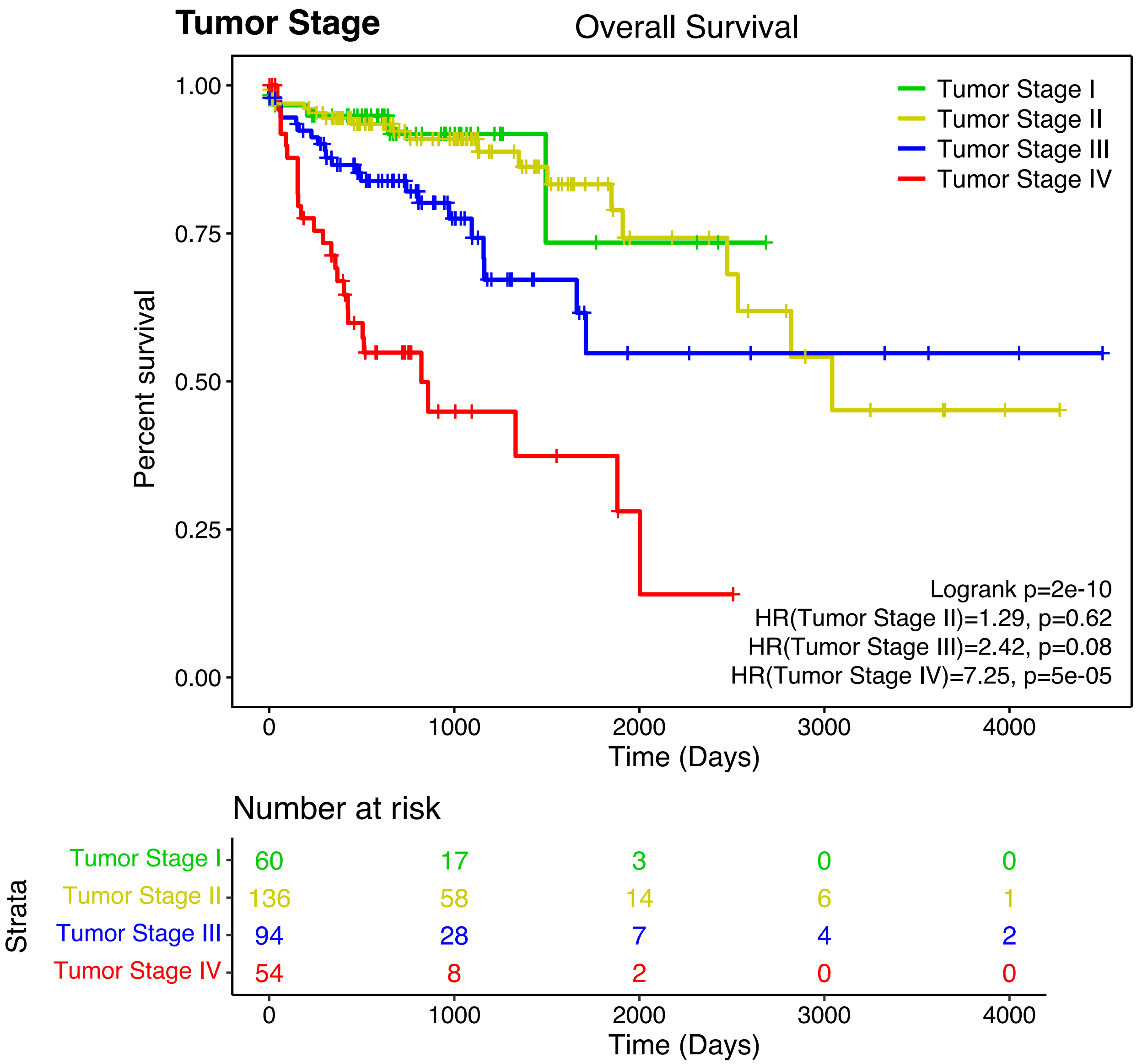

Fig. S24. Kaplan-Meier and Cox regression survival analyses assessing the impact of tumor stage on survival in colorectal cancer. A total of 358 colorectal tumors from the TCGA-COAD dataset were categorized according to AJCC pathologic stage (Stage I, II, III, or IV). This figure compares the survival rates among these groups to examine the relationship between tumor stage and survival outcomes.

Fig. S25.

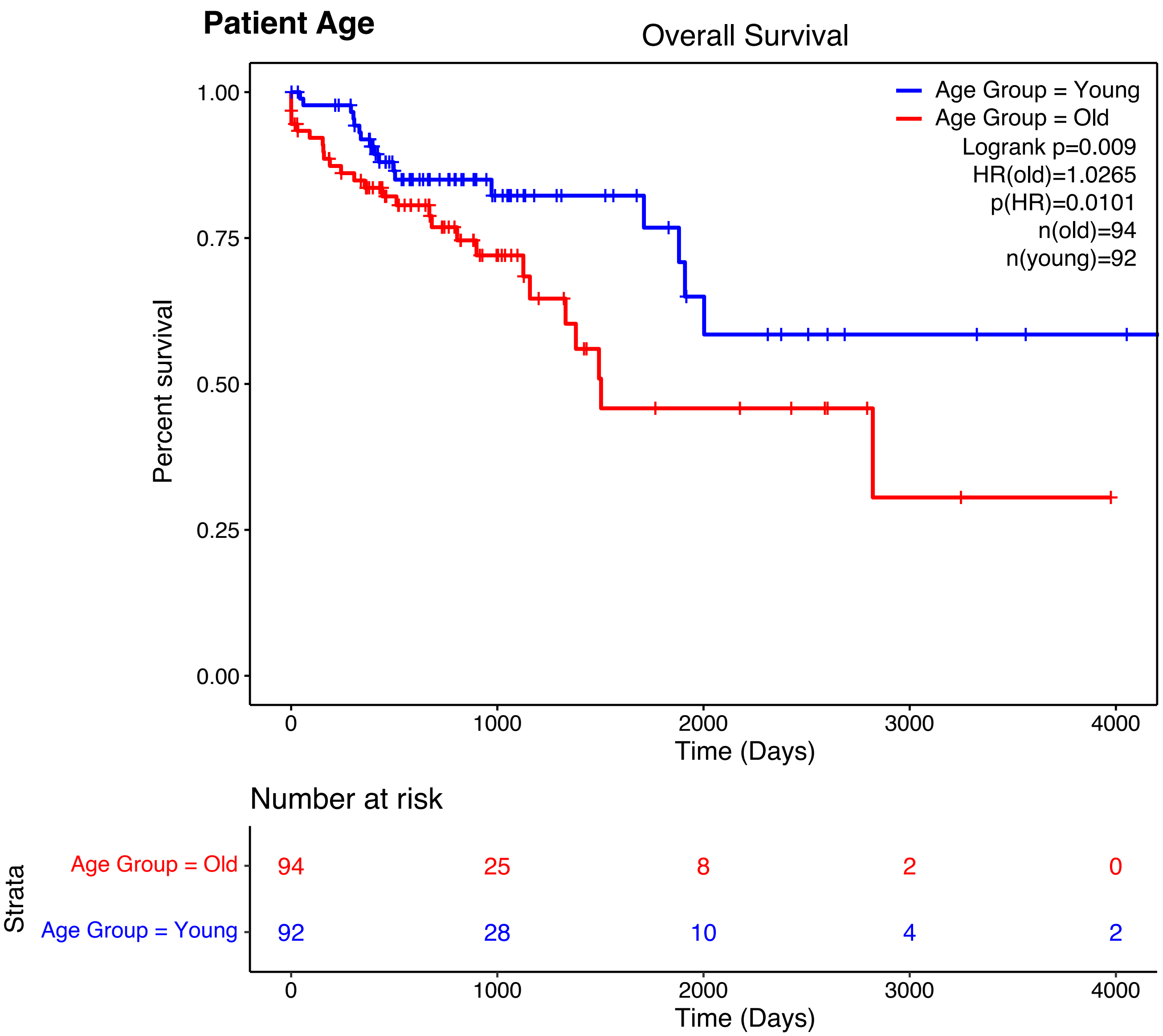

Fig. S25. Kaplan-Meier and Cox regression survival analyses based on patient age in colorectal cancer. A total of 358 colorectal tumors from the TCGA-COAD dataset were analyzed. Patients were divided into 'young' and 'old' age groups based on the upper and lower quartiles of age at index. This figure compares the survival rates between the two groups, assessing the impact of age on survival outcomes.

Fig. S26.

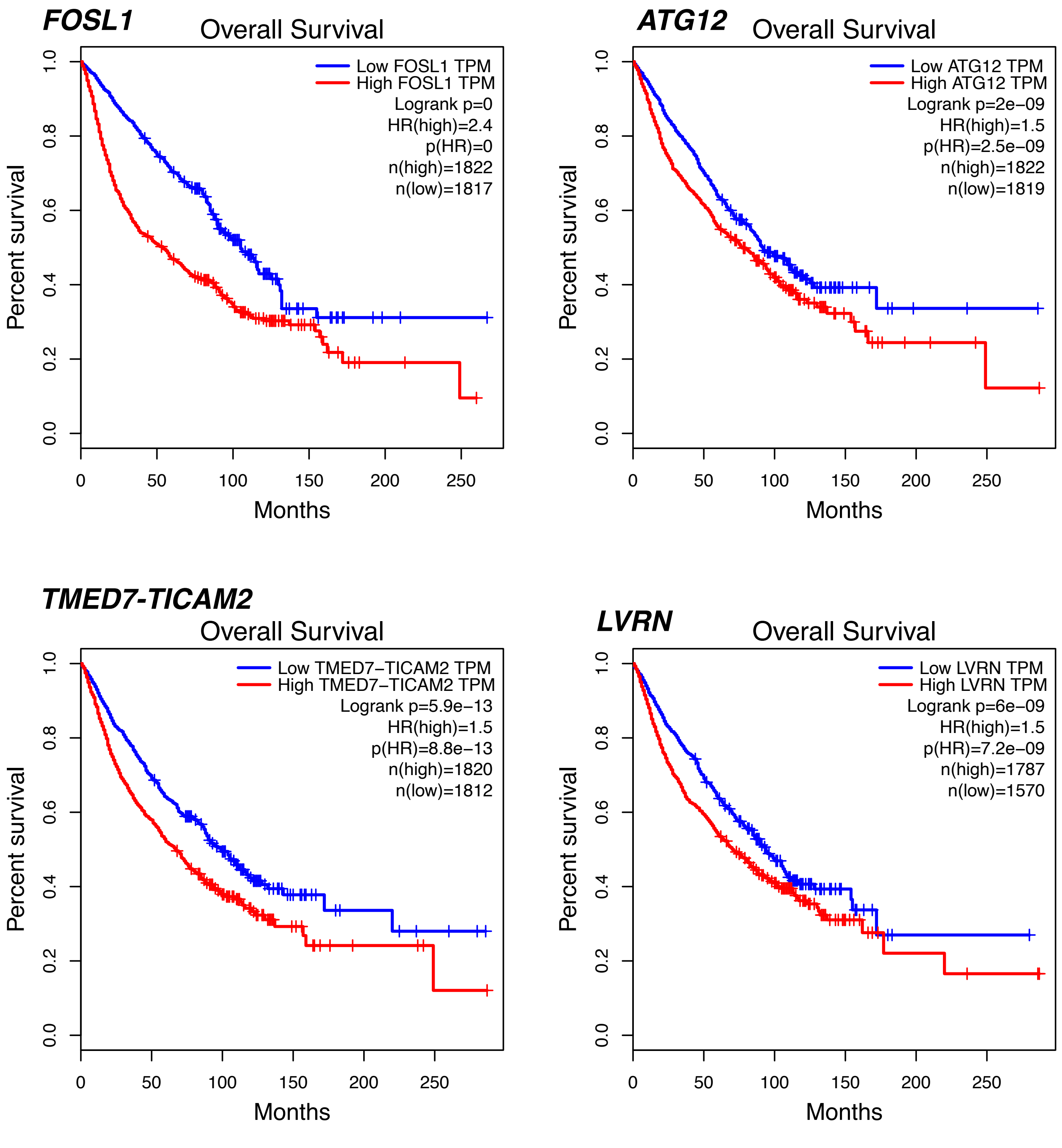

Fig. S26. Kaplan Meier and Cox regression survival analyses based on the expression of LTR10-associated genes, as calculated by GEPIA (gepia.cancer-pku.cn). A total of 7288 patient-derived tumors from 21 epithelial cancers (TCGA tumor abbreviations ACC, BLCA, BRCA, CESC, CHOL, COAD, ESCA, HNSC, KIRC, KIRP, LIHC, LUAD, LUSC, OV, PAAD, PRAD, READ, STAD, THCA, UCEC, UCS) were included in the analysis. For each gene, patients were divided into groups based on upper and lower quartiles of gene expression, and their survival rates were compared to evaluate the potential prognostic significance of these genes.

Fig. S27

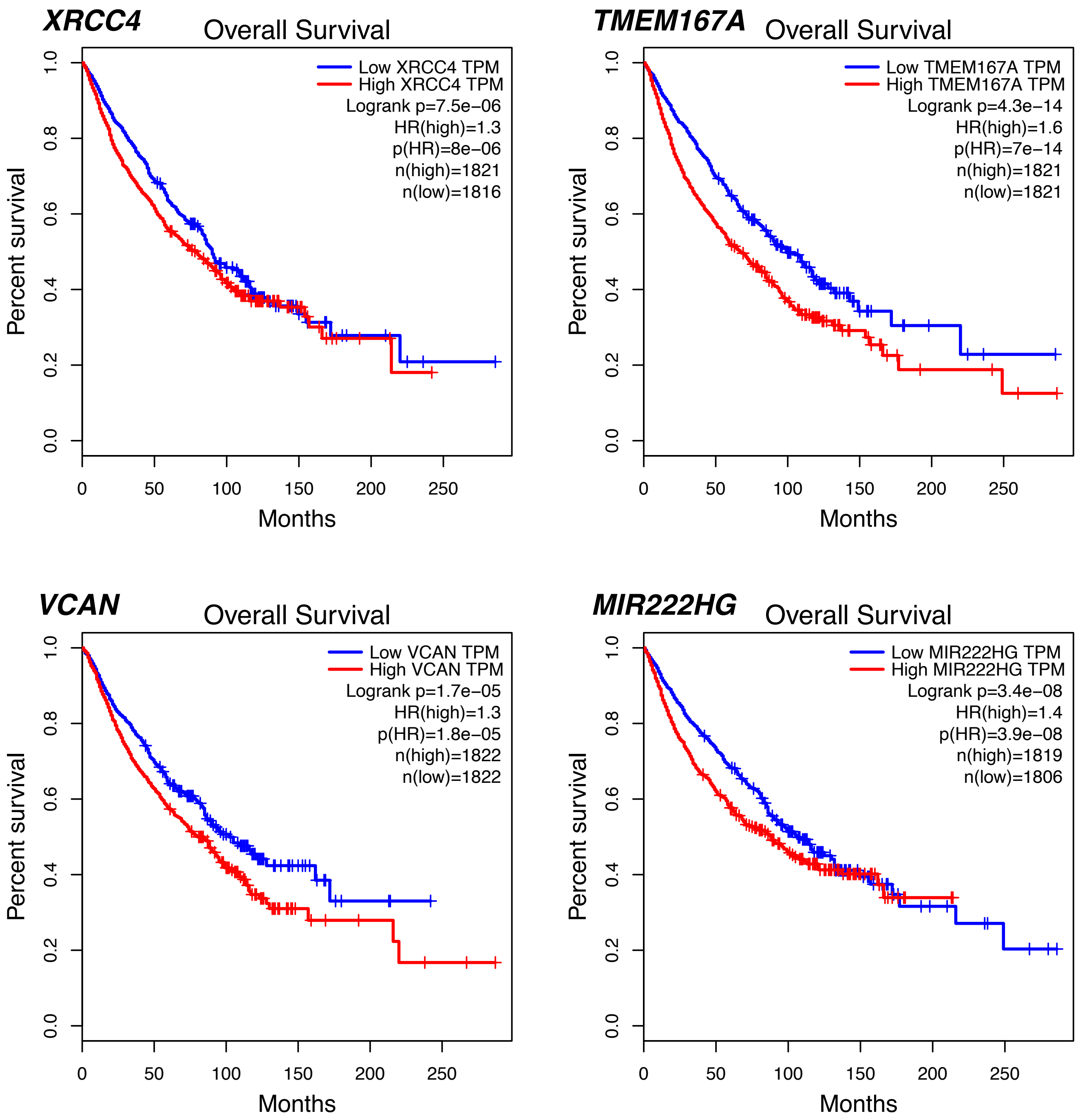

Fig. S27. Kaplan Meier and Cox regression survival analyses based on the expression of LTR10-associated genes, as calculated by GEPIA (gepia.cancer-pku.cn). A total of 7288 patient-derived tumors from 21 epithelial cancers (TCGA tumor abbreviations ACC, BLCA, BRCA, CESC, CHOL, COAD, ESCA, HNSC, KIRC, KIRP, LIHC, LUAD, LUSC, OV, PAAD, PRAD, READ, STAD, THCA, UCEC, UCS) were included in the analysis. For each gene, patients were divided into groups based on upper and lower quartiles of gene expression, and their survival rates were compared to evaluate the potential prognostic significance of these genes.

Fig. S28.

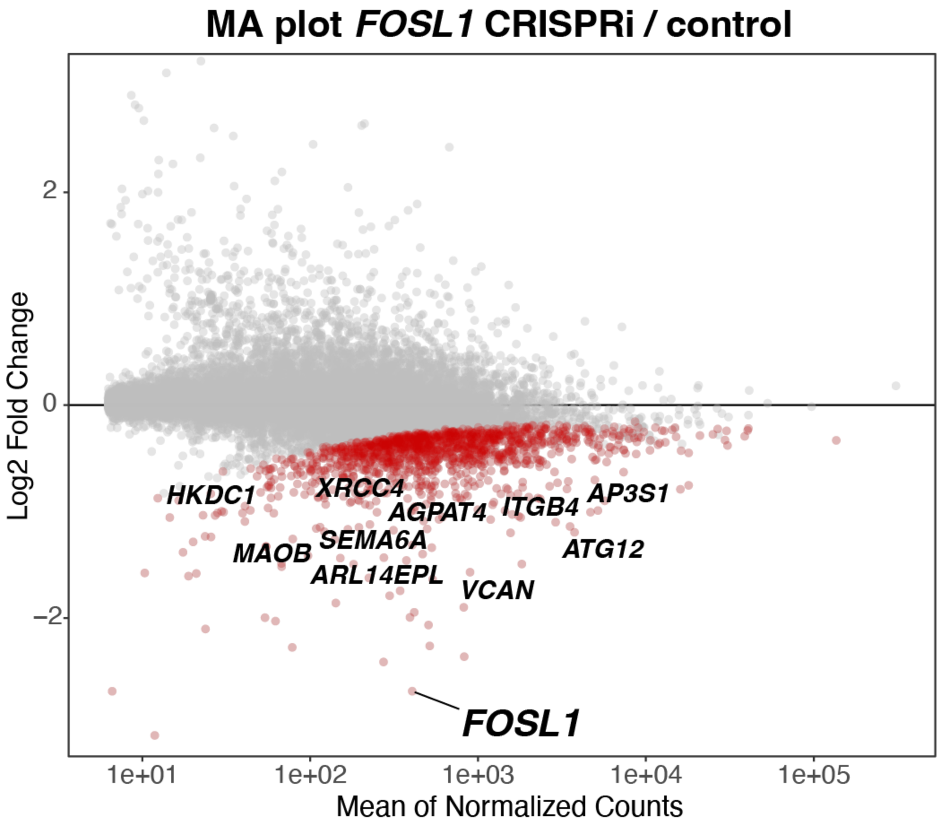

Fig. S28. MA plot showing global gene expression changes in HCT116 cells in response to silencing *FOSL1*. Significantly downregulated genes are shown in red. Text labels show the CRISPR target gene (*FOSL1*) as well as the 10 most significantly downregulated LTR10 target genes.

Fig. S29.

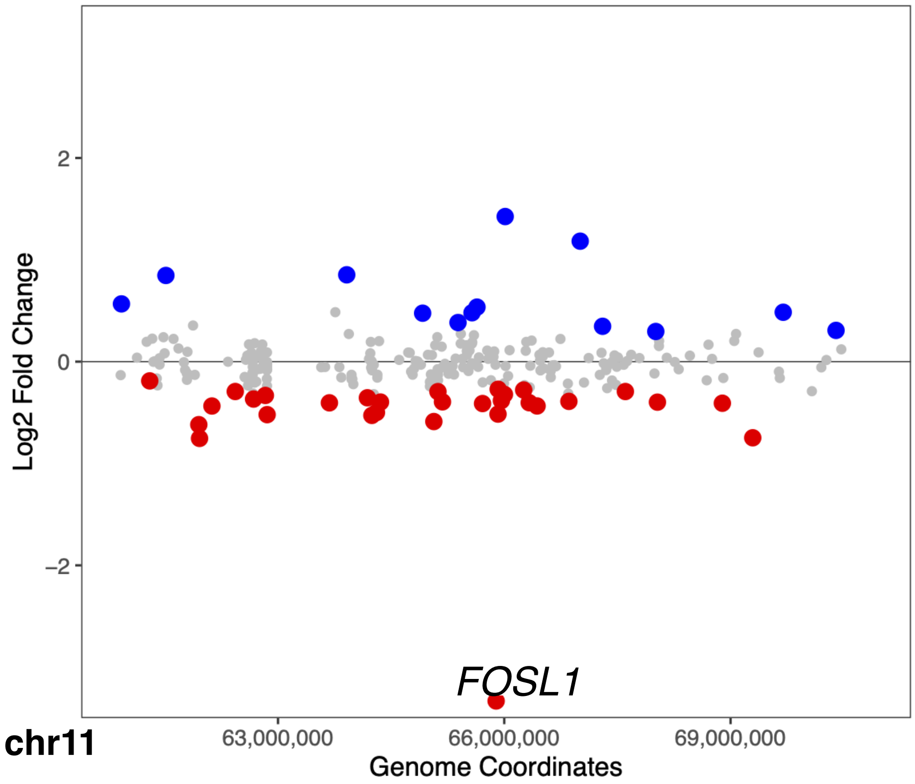

Fig. S29. Scatterplot of gene expression changes in response to silencing *FOSL1* in a 10Mb window around the target. Significantly downregulated genes are shown in red, significantly upregulated genes are shown in blue. The most significantly downregulated gene (*FOSL1*) is labelled.

Fig. S30.

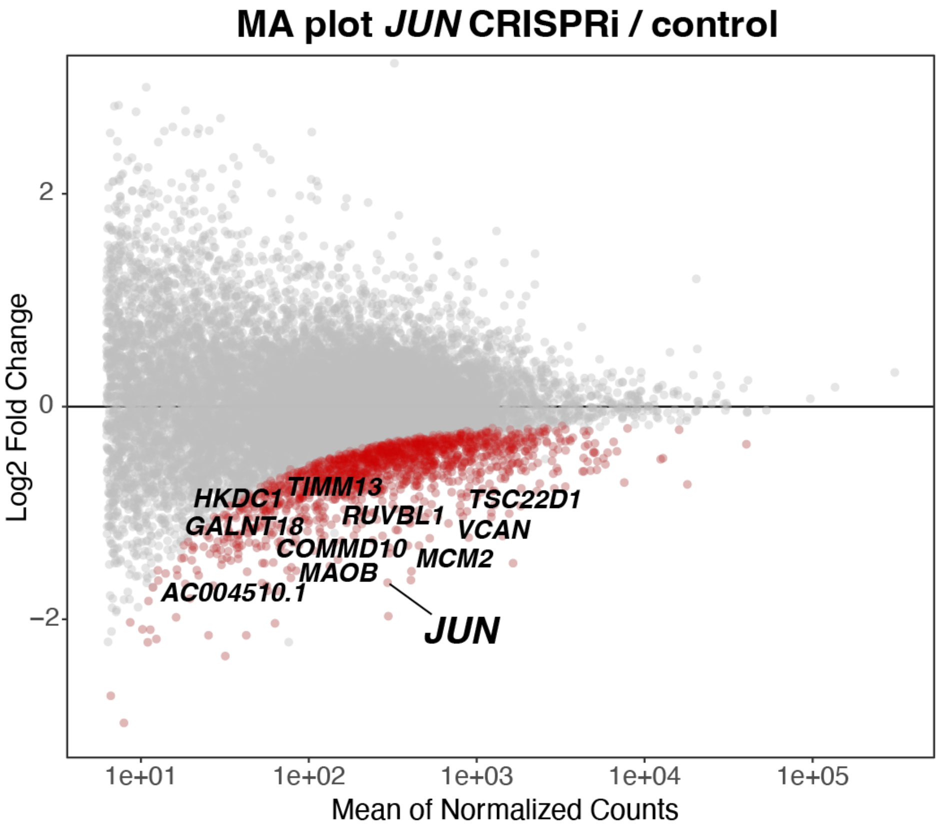

Fig. S30. MA plot showing global gene expression changes in HCT116 cells in response to silencing *JUN*. Significantly downregulated genes are shown in red. Text labels show the CRISPR target gene (*JUN*) as well as the 10 most significantly downregulated LTR10 target genes.

Fig. S31.

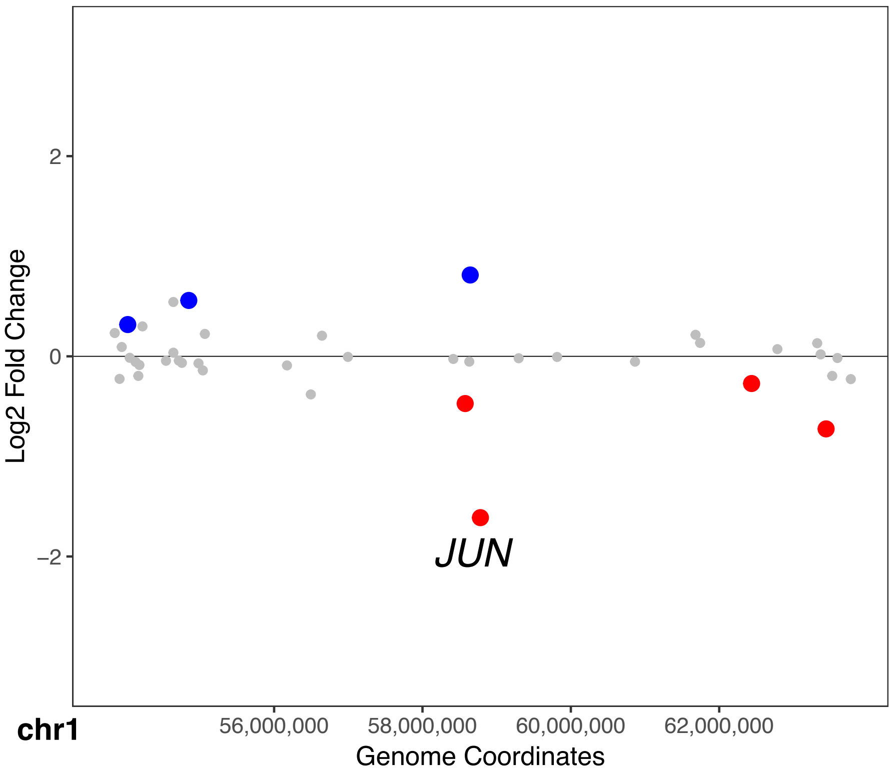

Fig. S31. Scatterplot of gene expression changes in response to silencing *JUN* in a 10Mb window around the target. Significantly downregulated genes are shown in red, significantly upregulated genes are shown in blue. The most significantly downregulated gene (*JUN*) is labelled.

Fig. S32.

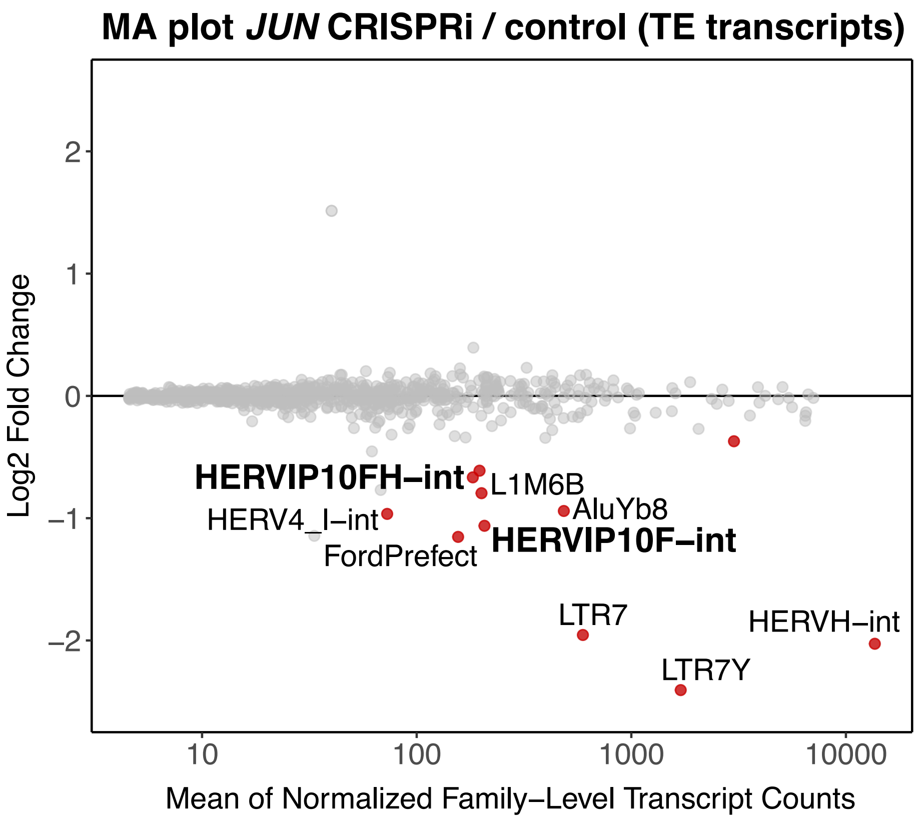

Fig. S32. MA plot of TE subfamilies showing significant differential expression in HCT116 cells subject to *JUN* silencing, based on RNA-seq. Significantly downregulated TE subfamilies are shown in red (adjusted $p<0.05$, $log2FC<0$). Significantly downregulated HERVI/LTR10 subfamilies are labeled in bold.

Fig. S33.

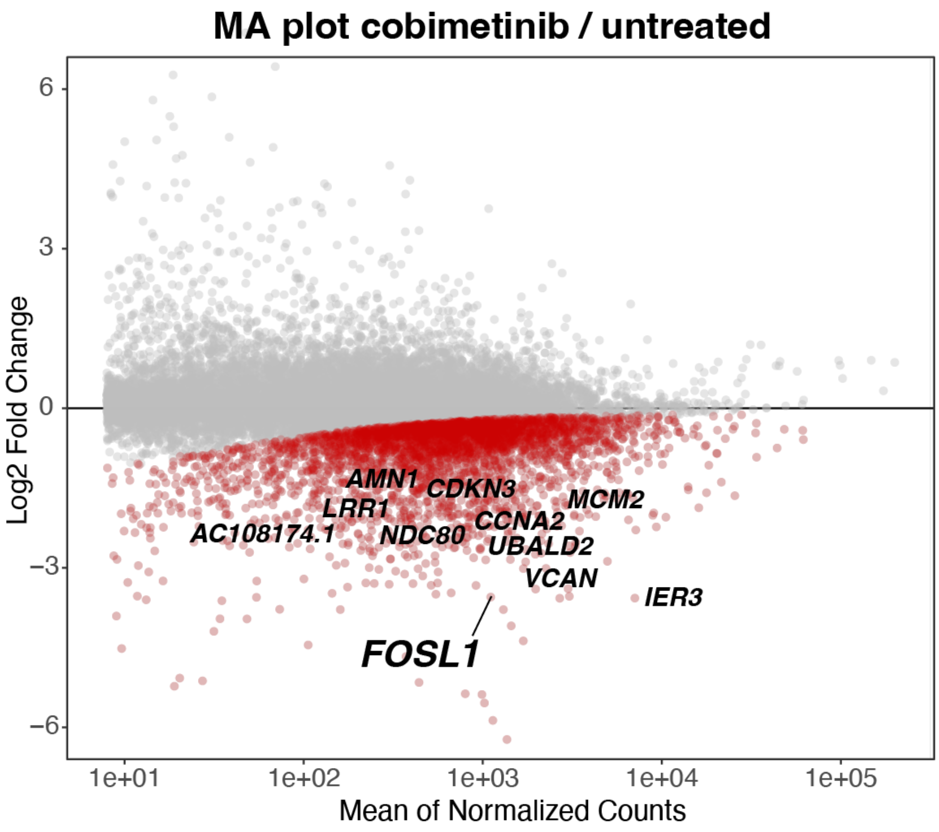

Fig. S33. MA plot showing global gene expression changes in HCT116 cells in response to 24hr cobimetinib treatment. Significantly downregulated genes are shown in red. Text labels show the most significantly downregulated AP1 subunit (*FOSL1*) as well as the 10 most significantly downregulated LTR10 target genes.

Fig. S34.

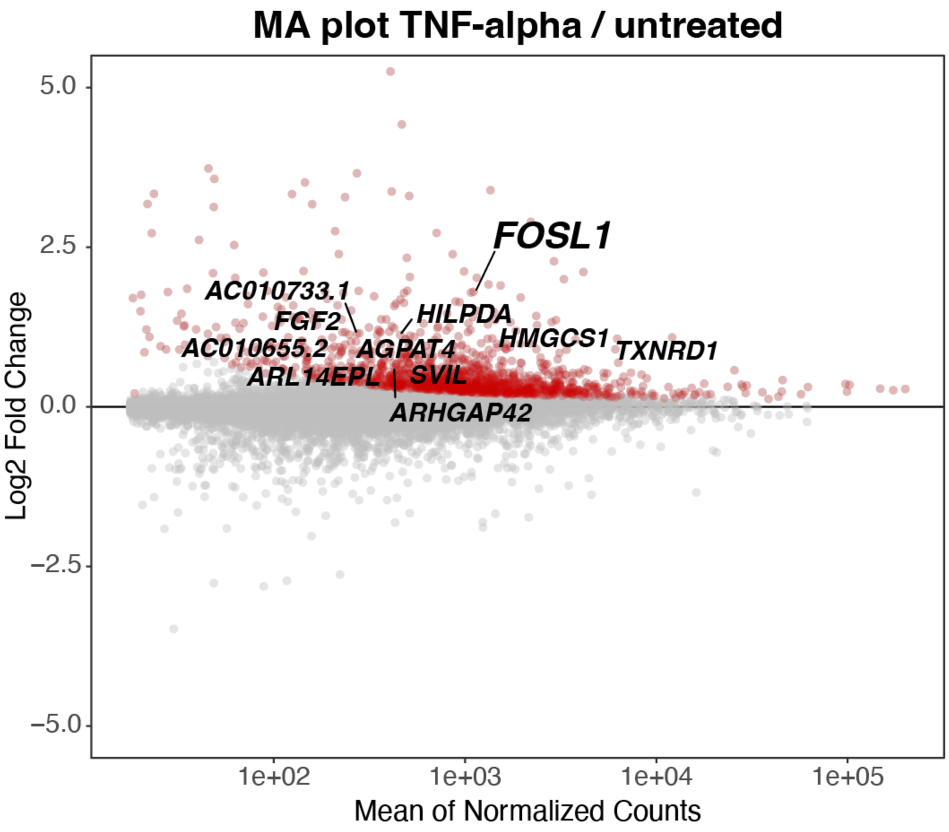

Fig. S34. MA plot showing global gene expression changes in cells in response to 24hr TNF-alpha treatment. Significantly upregulated genes are shown in red. Text labels show the most significantly upregulated AP1 subunit (*FOSL1*) as well as the 10 most significantly upregulated LTR10 target genes.

Fig. S35.

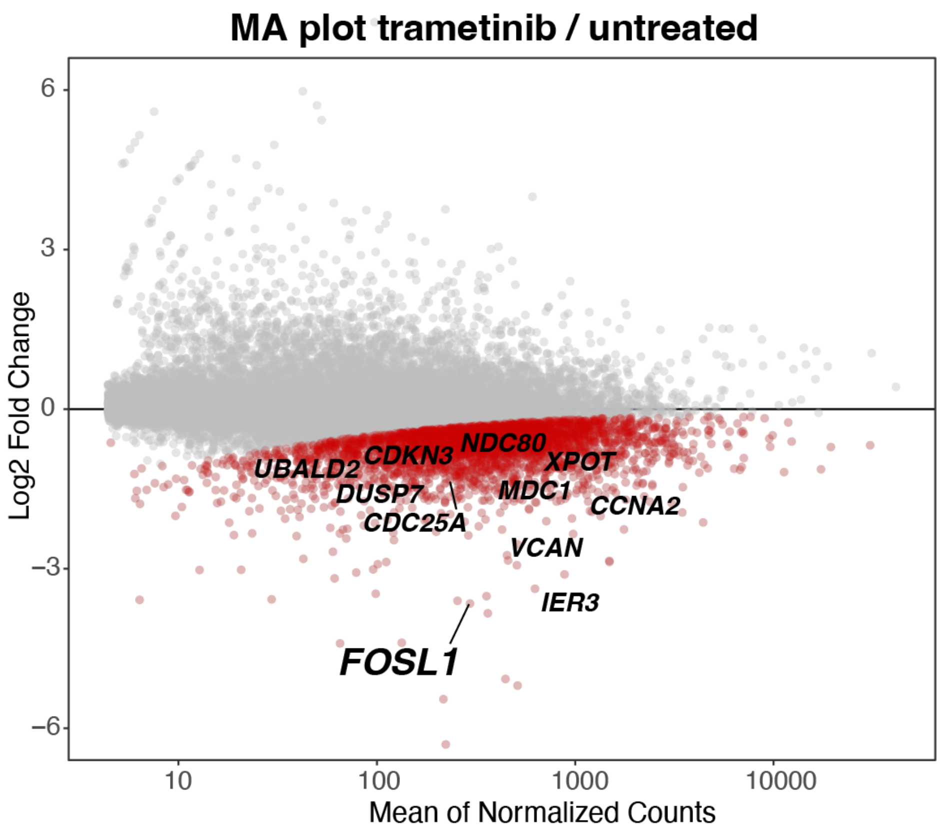

Fig. S35. MA plot showing global gene expression changes in HCT116 cells in response to 24hr trametinib treatment. Significantly downregulated genes are shown in red. Text labels show the most significantly downregulated AP1 subunit (*FOSL1*) as well as the 10 most significantly downregulated LTR10 target genes. RNA-seq data was downloaded from a published dataset (GEO accession GSE78519).

Fig. S36.

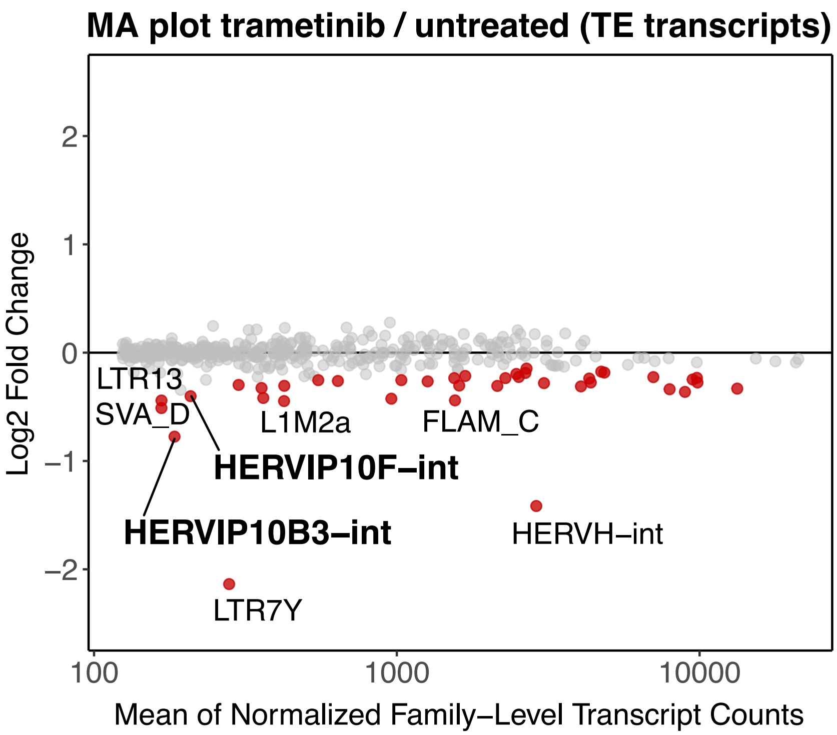

Fig. S36. MA plot of TE subfamilies showing significant differential expression in HCT116 cells in response to 24hr trametinib treatment. Significantly downregulated TE subfamilies are shown in red. Significantly downregulated HERVI/LTR10 subfamilies are labeled in bold. RNA-seq data was downloaded from a published dataset (GEO accession GSE78519).

Fig. S37.

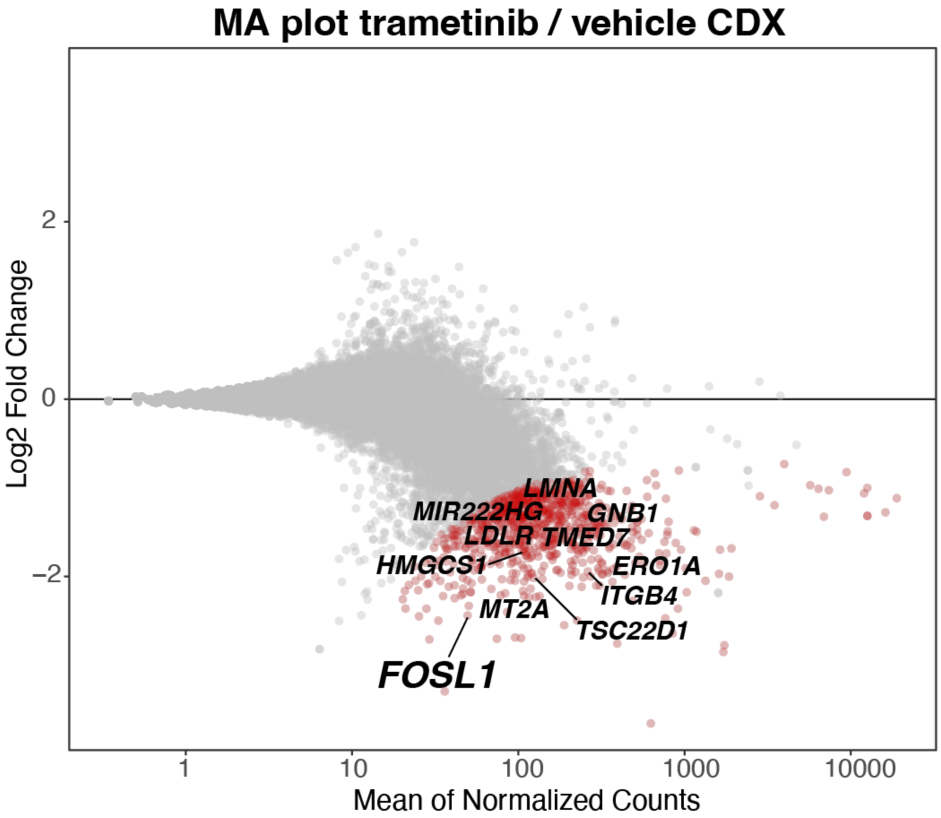

Fig. S37. MA plot showing global gene expression changes in inhouse HCT116 cell line-based xenografts (CDXs), comparing trametinib treatment to vehicle controls. Significantly downregulated genes are shown in red. Text labels show the most significantly downregulated AP1 subunit (*FOSL1*) as well as the 10 most significantly downregulated LTR10 target genes.

Fig. S38.

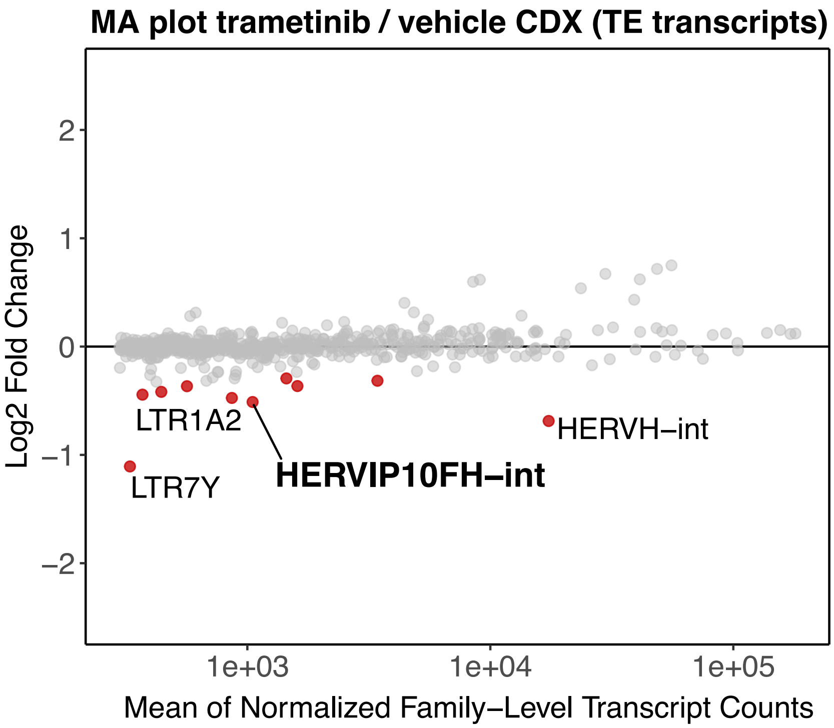

Fig. S38. MA plot of TE subfamilies showing significant differential expression in inhouse HCT116 cell line-based xenografts (CDXs), comparing trametinib treatment to vehicle controls. Significantly downregulated TE subfamilies are shown in red. Significantly downregulated HERVI/LTR10 subfamilies are labeled in bold.

Fig. S39.

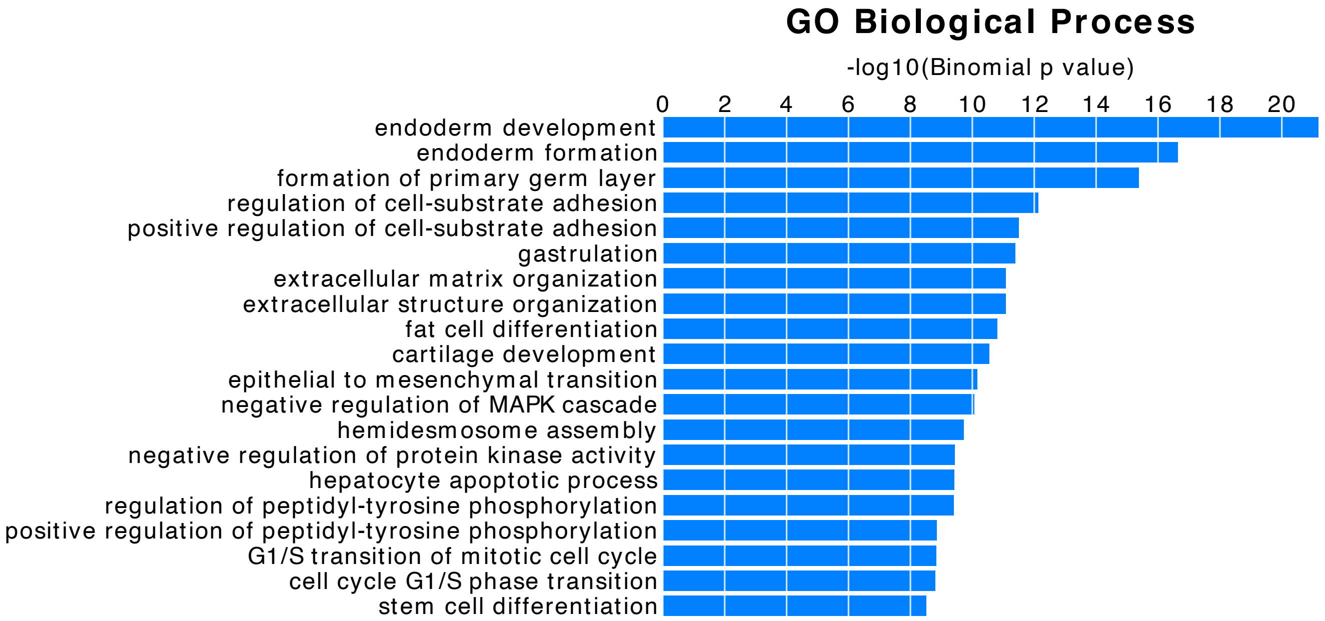

Fig. S39. Bargraph of Gene Ontology (GO) biological processes associated with the set of H3K27ac regions significantly downregulated by cobimetinib (n=1634), as predicted by GREAT.

Fig. S40.

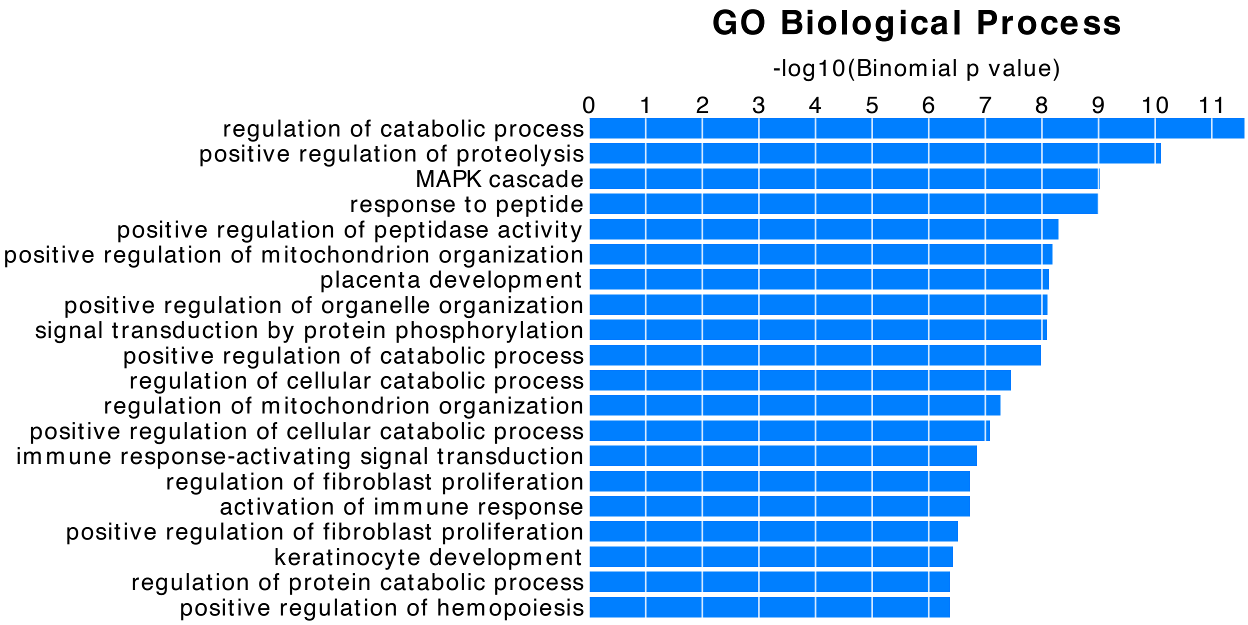

Fig. S40. Bargraph of Gene Ontology (GO) biological processes associated with the set of H3K27ac regions significantly upregulated by TNF-alpha (n=775), as predicted by GREAT.

Fig. S41.

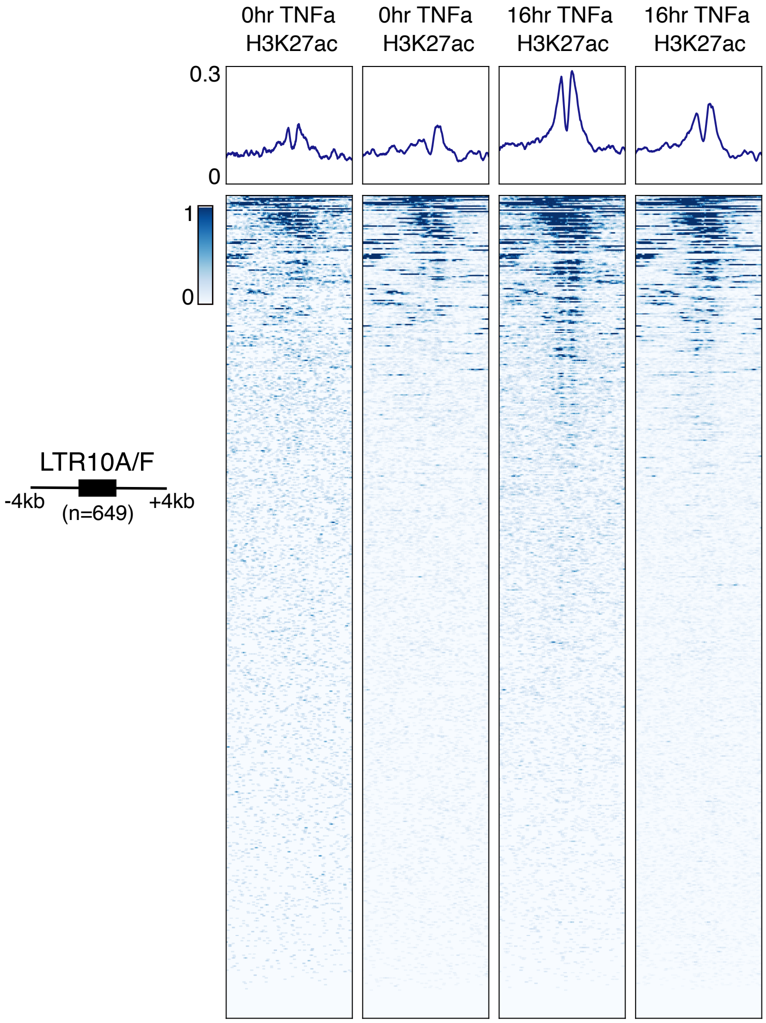

Fig. S41. Heatmap of H3K37ac ChIP-seq from SW480 colon cancer cells, either untreated or treated with TNF-alpha for 16hr, over the merged set of 649 LTR10A/F elements (GSE102796). Metaprofiles represent the normalized signal across elements.

Fig. S42.

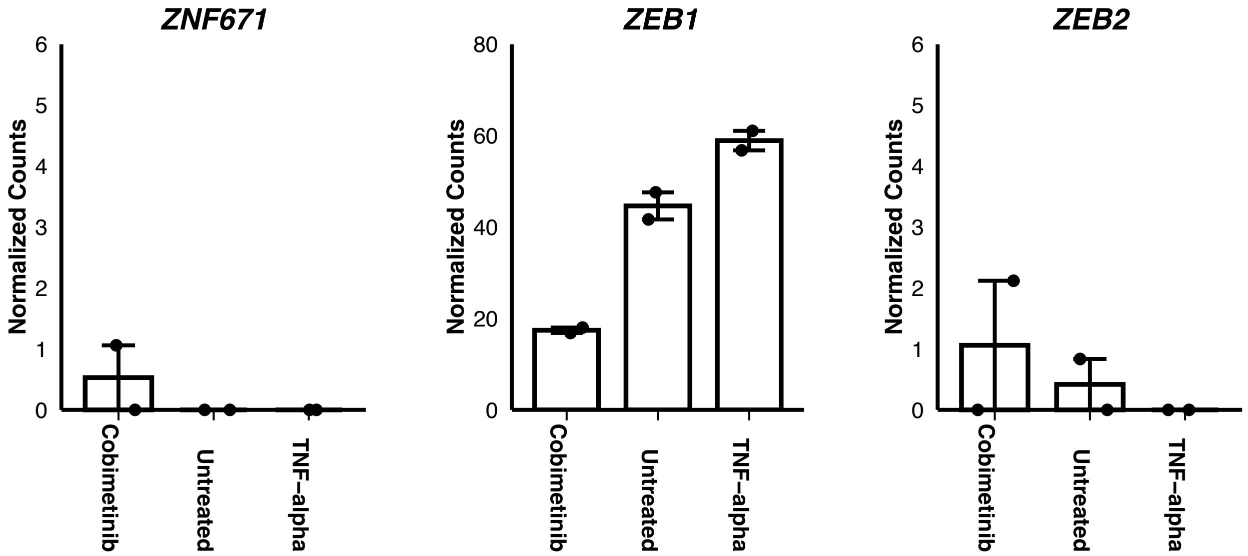

Fig. S42. Normalized RNA-seq expression values of predicted LTR10 repressors *ZNF671*, *ZEB1*, and *ZEB2* in HCT116 cells. Cells were treated with 24 hr cobimetinib (n=2), 24 hr TNF-alpha (n=2), or untreated (n=2). Points on the x-axis indicate normalized counts of zero.

Fig. S43.

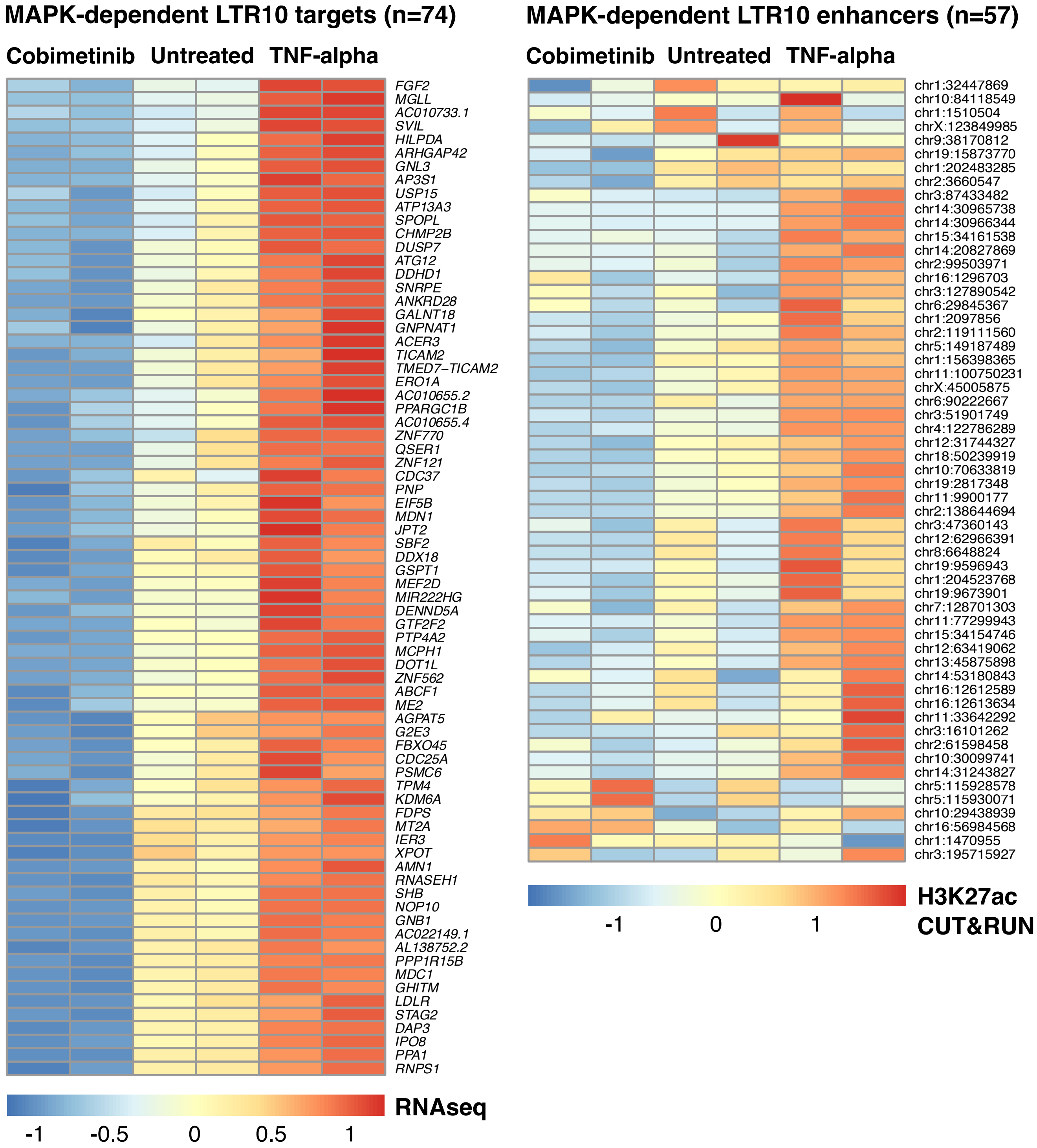

Fig. S43. Heatmaps of normalized RNA-seq expression values (left) and normalized H3K27ac CUT&RUN signal (right) for MAPK-dependent predicted LTR10 target genes and enhancers. Normalized values for each treatment replicate are shown.

Fig. S44.

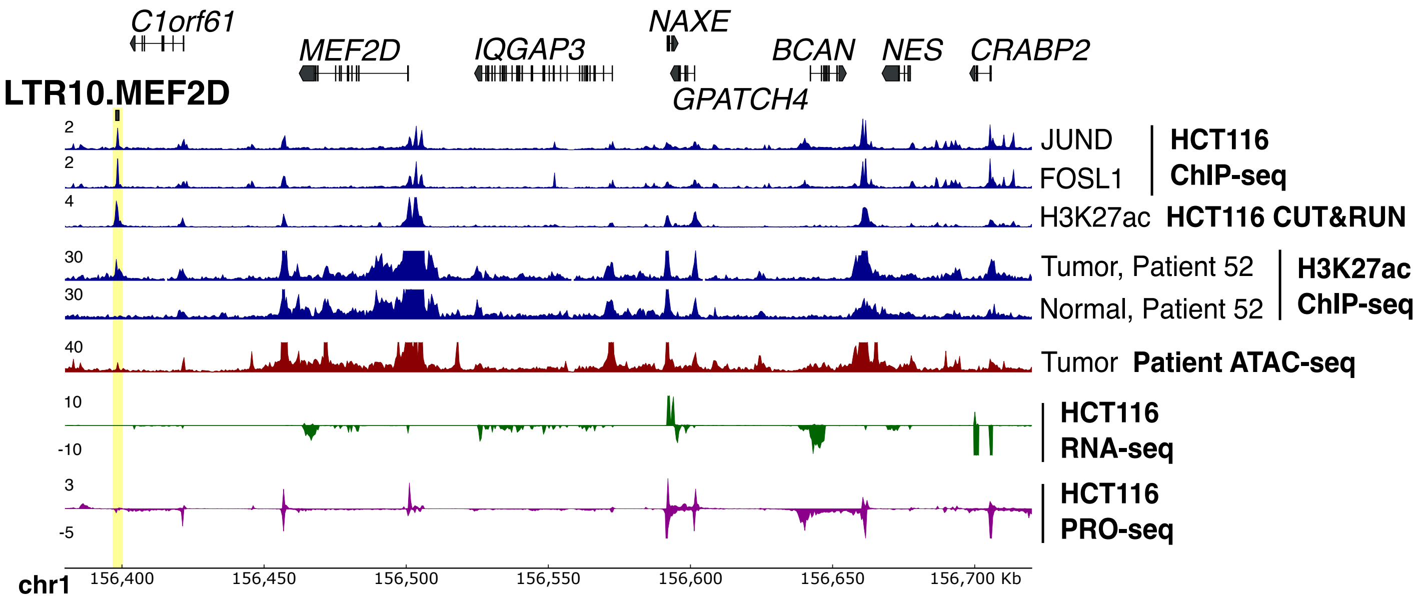

Fig. S44. Genome browser screenshot of the *MEF2D* locus with the LTR10.MEF2D enhancer labeled. From top to bottom: JUND and FOSL1 ChIP-seq (GSE32465), H3K27ac CUT&RUN (in-house), H3K27ac ChIP-seq from matched tumor/normal samples from the CEMT Canadian Epigenome Project (patient AKCC52), tumor ATAC-seq from TCGA (patient COAD P022), HCT116 RNA-seq (in-house), and HCT116 PRO-seq (GSE129501). Axis numbers represent the upper limit of the range; the lower limit is always zero.

Fig. S45.

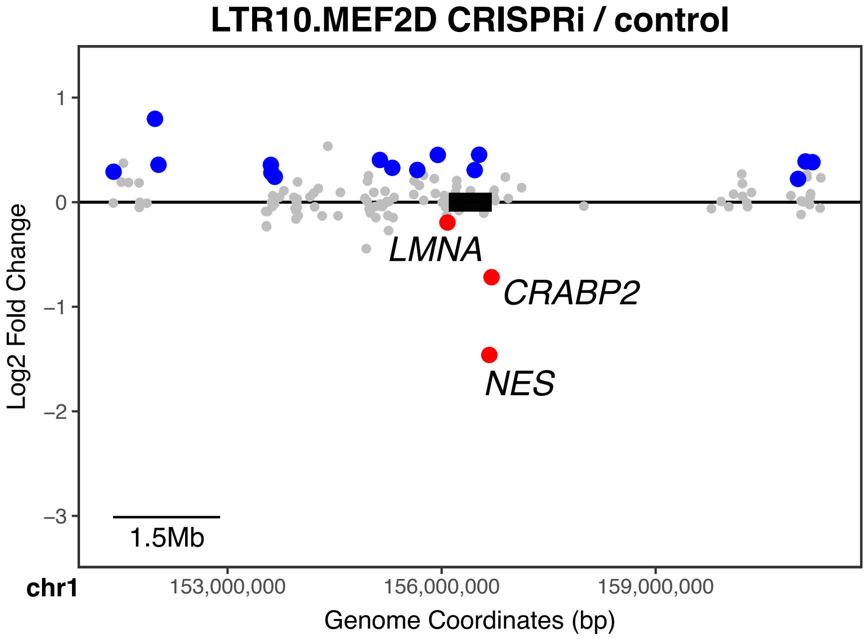

Fig. S45. Scatterplot of local gene expression changes in response to CRISPR silencing of the LTR10.MEF2D element. Significantly downregulated genes are shown in red; significantly upregulated genes are shown in blue. Significantly downregulated genes within 1.5 MB of the targeted element are labeled (element box not drawn to scale).

Fig. S46.

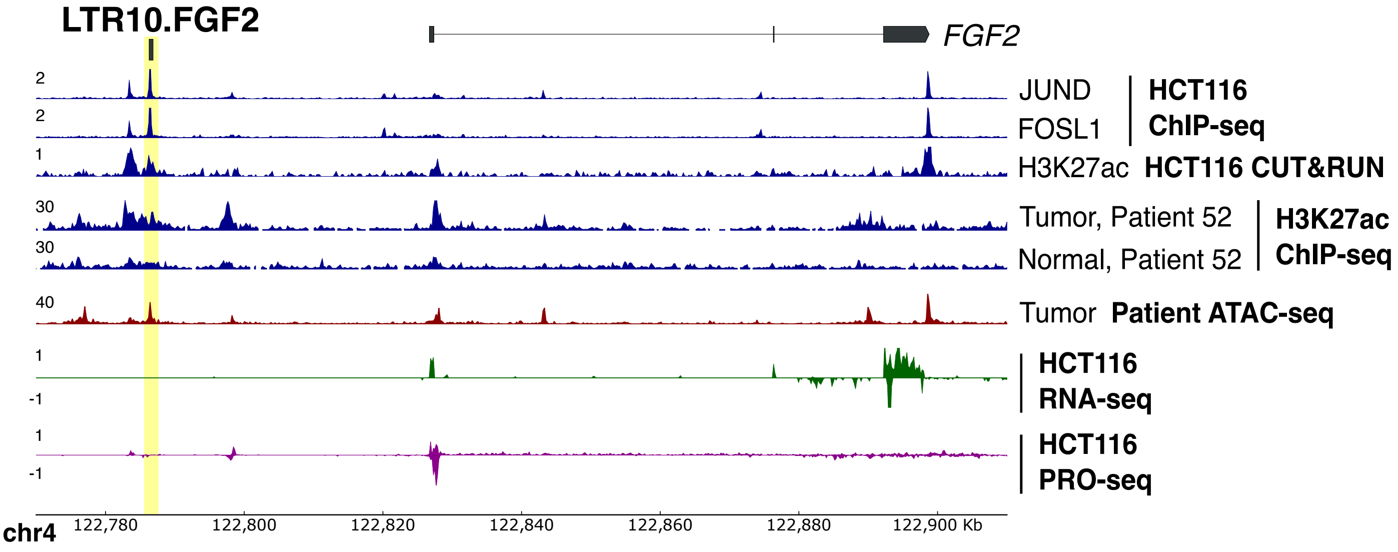

Fig. S46. Genome browser screenshot of the *FGF2* locus with the LTR10.FGF2 enhancer labeled. From top to bottom: JUND and FOSL1 ChIP-seq (GSE32465), H3K27ac CUT&RUN (in-house), H3K27ac ChIP-seq from matched tumor/normal samples from the CEMT Canadian Epigenome Project (patient AKCC52), tumor ATAC-seq from TCGA (patient COAD P022), HCT116 RNA-seq (in-house), and HCT116 PRO-seq (GSE129501). Axis numbers represent the upper limit of the range; the lower limit is always zero.

Fig. S47.

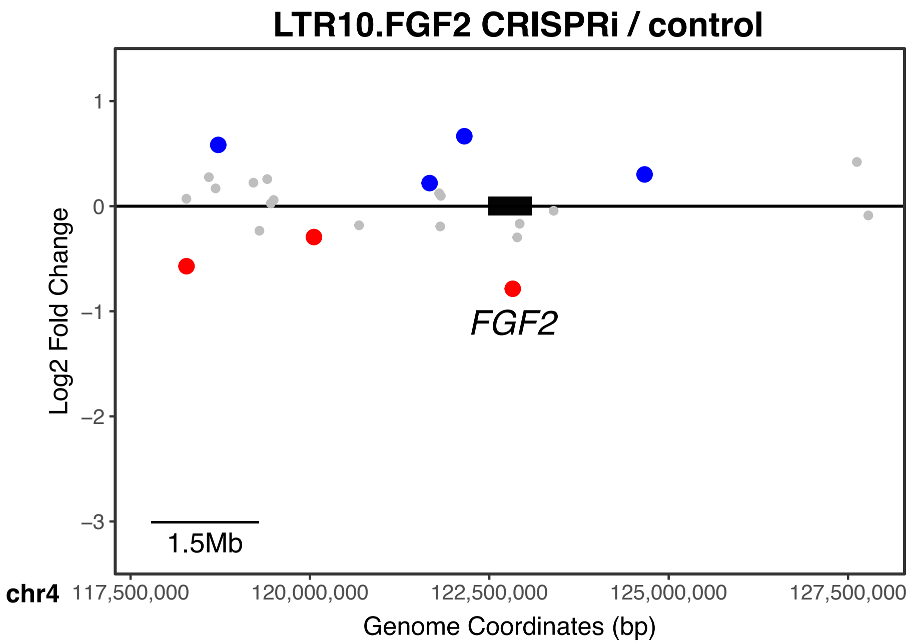

Fig. S47. Scatterplot of local gene expression changes in response to CRISPR silencing of the LTR10.FGF2 element. Significantly downregulated genes are shown in red; significantly upregulated genes are shown in blue. Significantly downregulated genes within 1.5 MB of the targeted element are labeled (element box not drawn to scale).

Fig. S48.

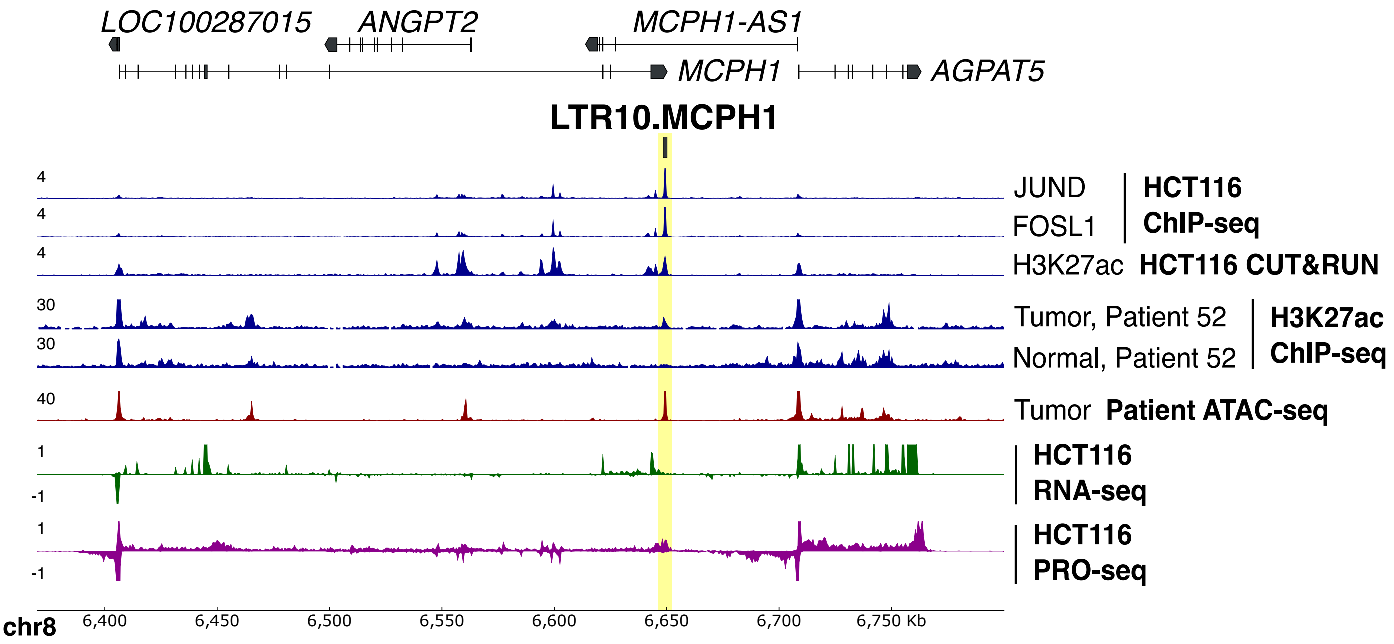

Fig. S48. Genome browser screenshot of the *MCPH1* locus with the LTR10.MCPH1 enhancer labeled. From top to bottom: JUND and FOSL1 ChIP-seq (GSE32465), H3K27ac CUT&RUN (in-house), H3K27ac ChIP-seq from matched tumor/normal samples from the CEMT Canadian Epigenome Project (patient AKCC52), tumor ATAC-seq from TCGA (patient COAD P022), HCT116 RNA-seq (in-house), and HCT116 PRO-seq (GSE129501). Axis numbers represent the upper limit of the range; the lower limit is always zero.

Fig. S49.

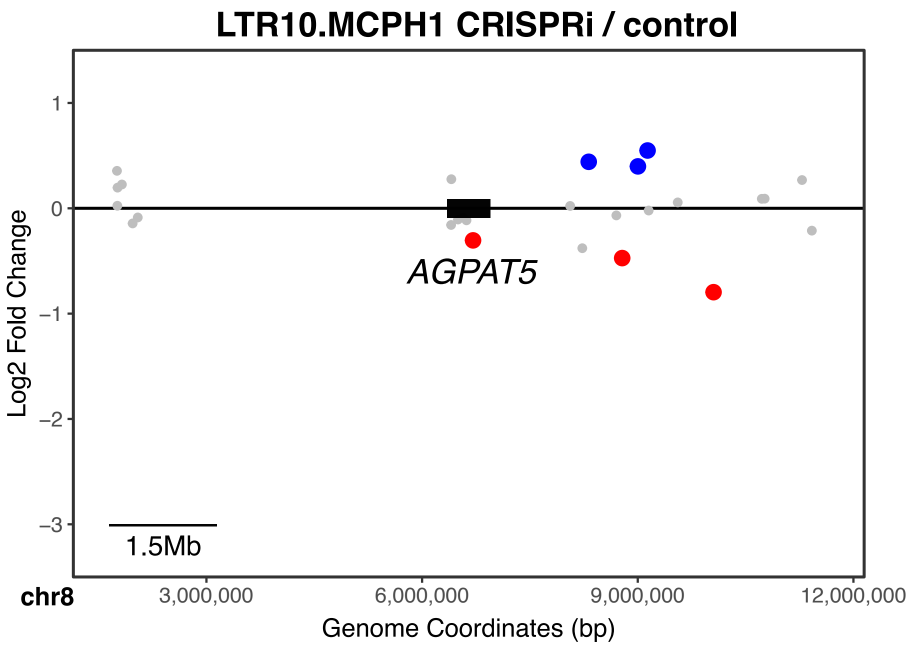

Fig. S49. Scatterplot of local gene expression changes in response to CRISPR silencing of the LTR10.MCPH1 element. Significantly downregulated genes are shown in red; significantly upregulated genes are shown in blue. Significantly downregulated genes within 1.5 MB of the targeted element are labeled (element box not drawn to scale).

Fig. S50.

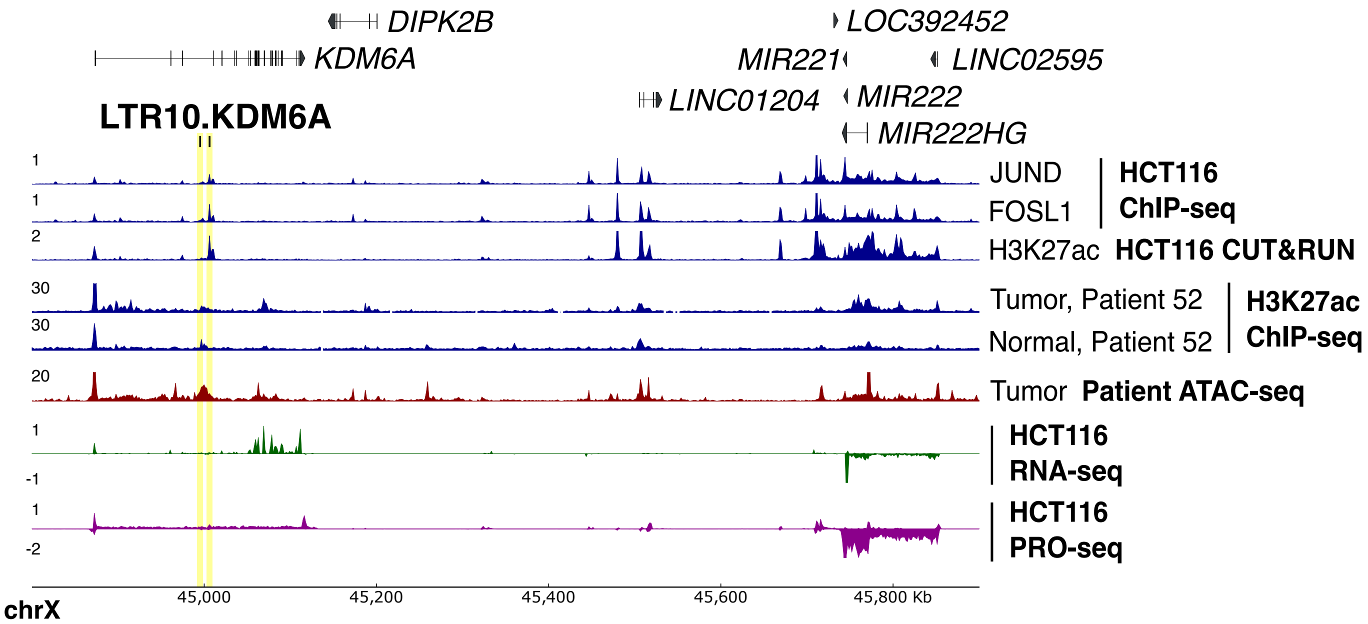

Fig. S50. Genome browser screenshot of the *KDM6A* locus with the LTR10.KDM6A enhancer labeled. From top to bottom: JUND and FOSL1 ChIP-seq (GSE32465), H3K27ac CUT&RUN (in-house), H3K27ac ChIP-seq from matched tumor/normal samples from the CEMT Canadian Epigenome Project (patient AKCC52), tumor ATAC-seq from TCGA (patient COAD P022), HCT116 RNA-seq (in-house), and HCT116 PRO-seq (GSE129501). Axis numbers represent the upper limit of the range; the lower limit is always zero.

Fig. S51.

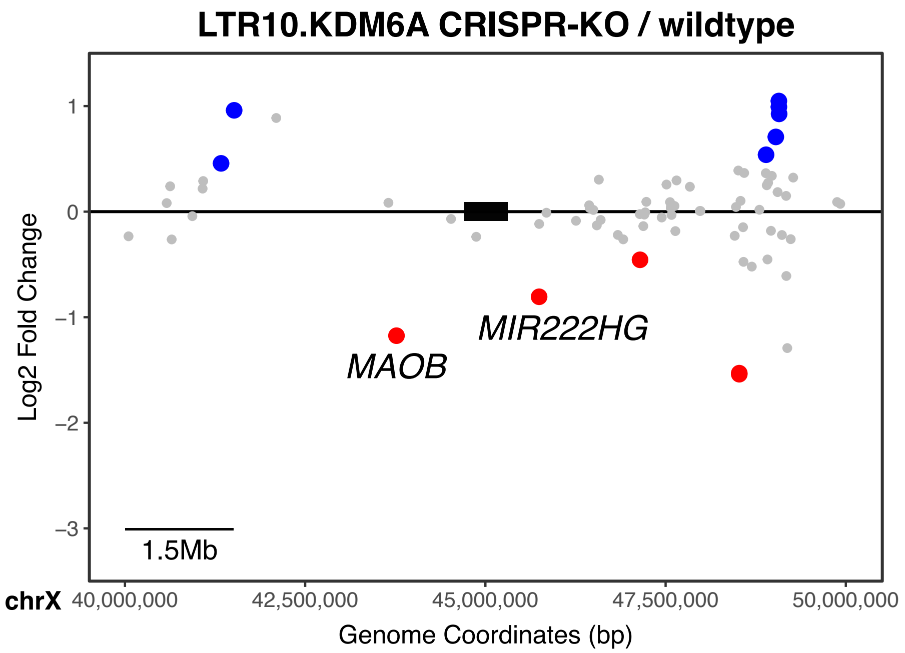

Fig. S51. Scatterplot of local gene expression changes in response to CRISPR deletion of the LTR10.KDM6A element. Multiple CRISPR deletion KO clones were generated and compared against wildtype HCT116 cells. Significantly downregulated genes are shown in red; significantly upregulated genes are shown in blue. Significantly downregulated genes within 1.5 MB of the targeted element are labeled (element box not drawn to scale).

Fig. S52.

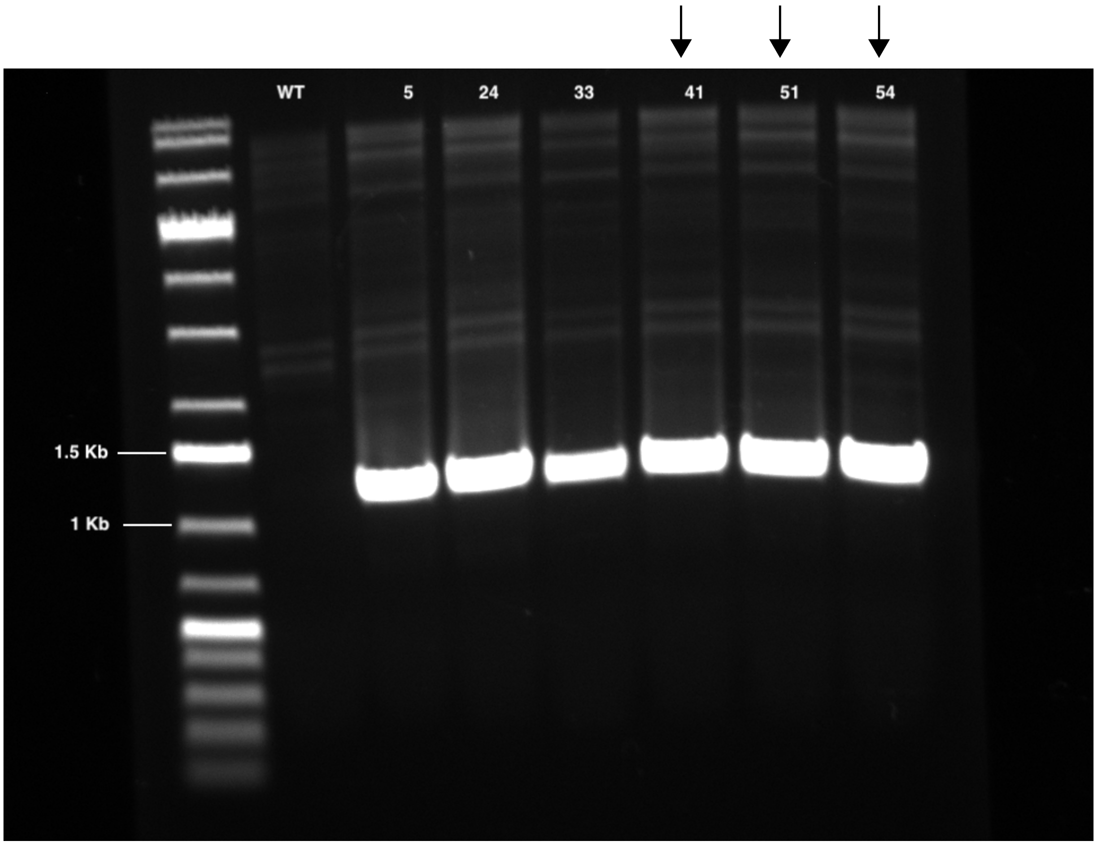

Fig. S52. External PCR validation of LTR10.KDM6A CRISPR KO clones. PCR primers used for validation (KDM6A_up_external, KDM6A_down_external) flank LTR10.KDM6A element. Expected KO amplicon size is 1245 bp. Expected wildtype amplicon size (unobserved) is 8854 bp. Arrows indicate clones that were used for DEseq2 analysis against wildtype HCT116 cells, based on PCA results.

Fig. S53.

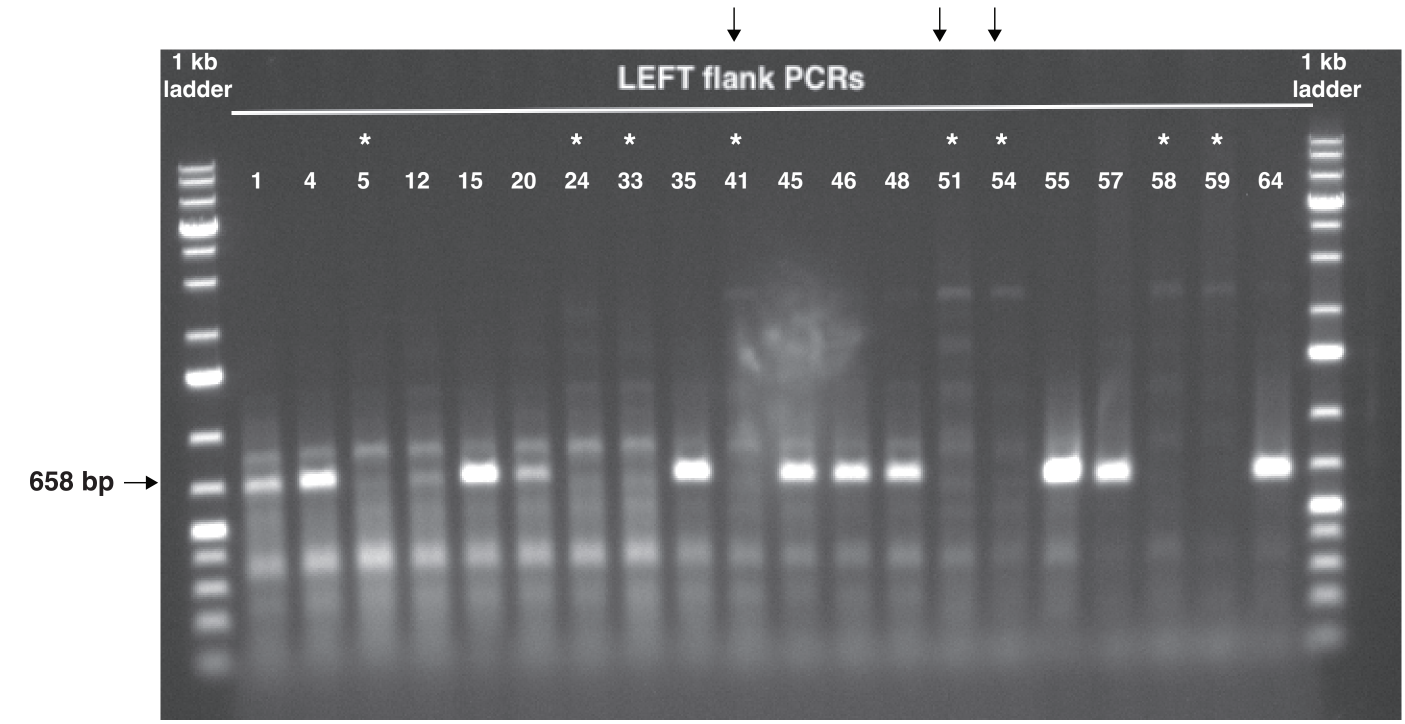

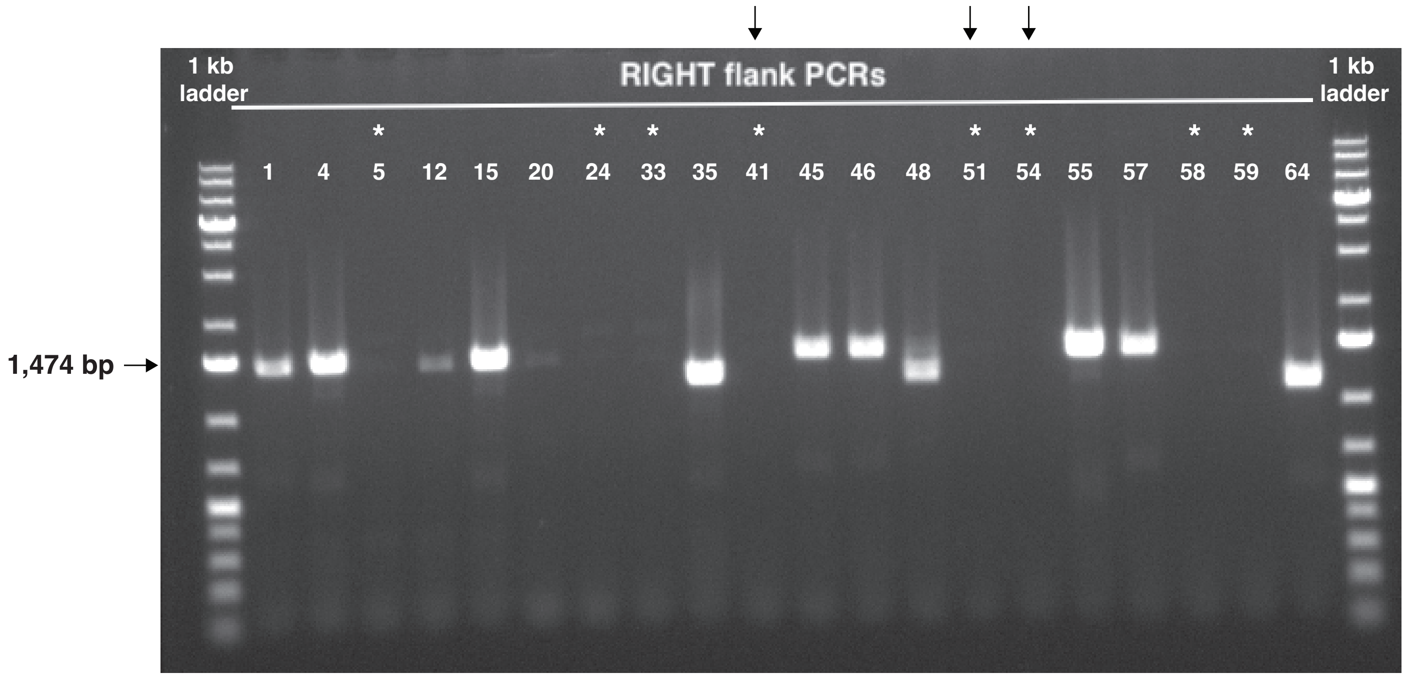

Fig. S53. Internal PCR validation of LTR10.KDM6A CRISPR KO clones. Expected wildtype amplicon size is 658 bp for upstream PCR (left flank), 1474 bp for downstream PCR (right flank). Asterisks indicate homozygous KO clones, based on no product from both flanks. Arrows indicate clones that were used for DEseq2 analysis against wildtype HCT116 cells, based on PCA results.

Fig. S54.

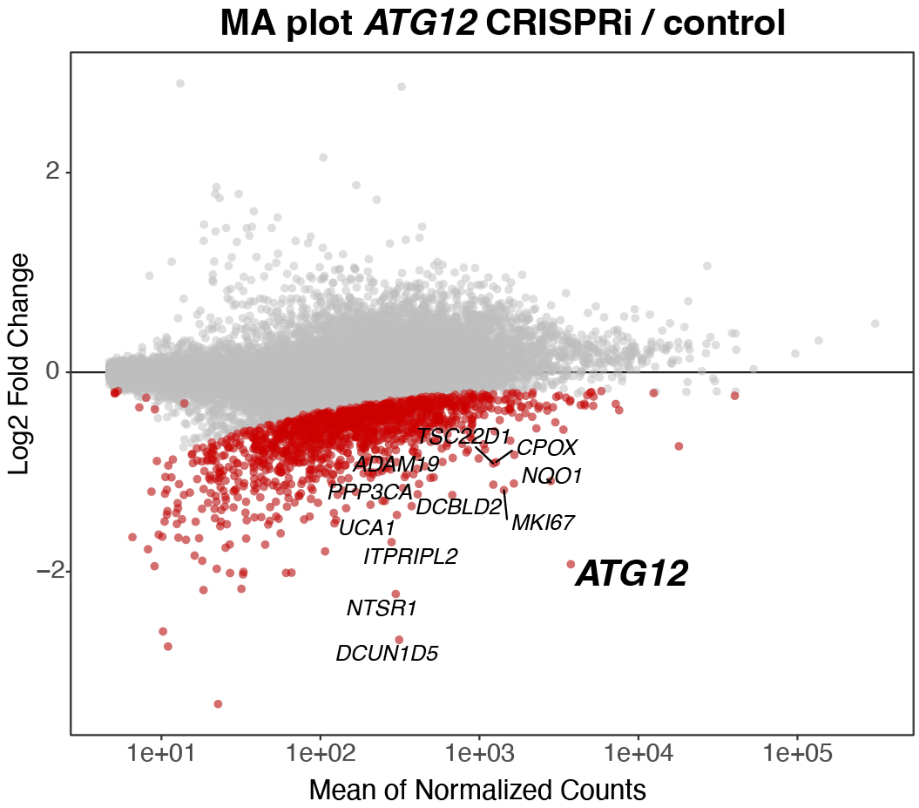

Fig. S54. MA plot showing global gene expression changes in cells in response to silencing *ATG12* (TSS). Significantly downregulated genes are shown in red.

Fig. S55.

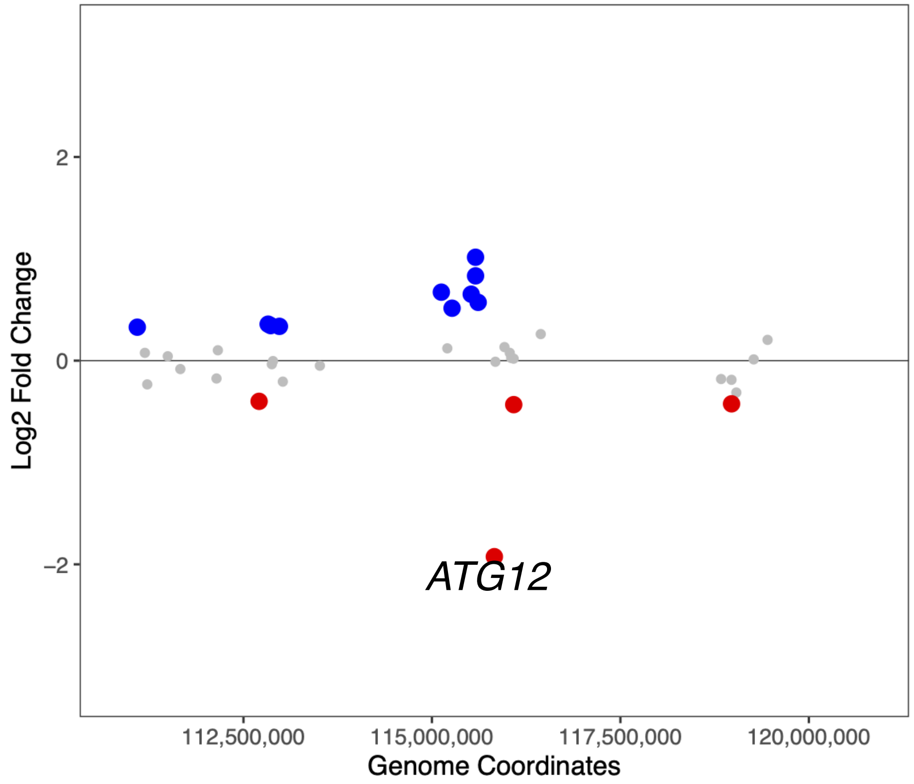

Fig. S55. Scatterplot of gene expression changes in response to silencing *ATG12* (TSS) in a 10Mb window around the target. Significantly downregulated genes are shown in red, significantly upregulated genes are shown in blue. The most significantly downregulated gene (*ATG12*) is labelled.

Fig. S56.

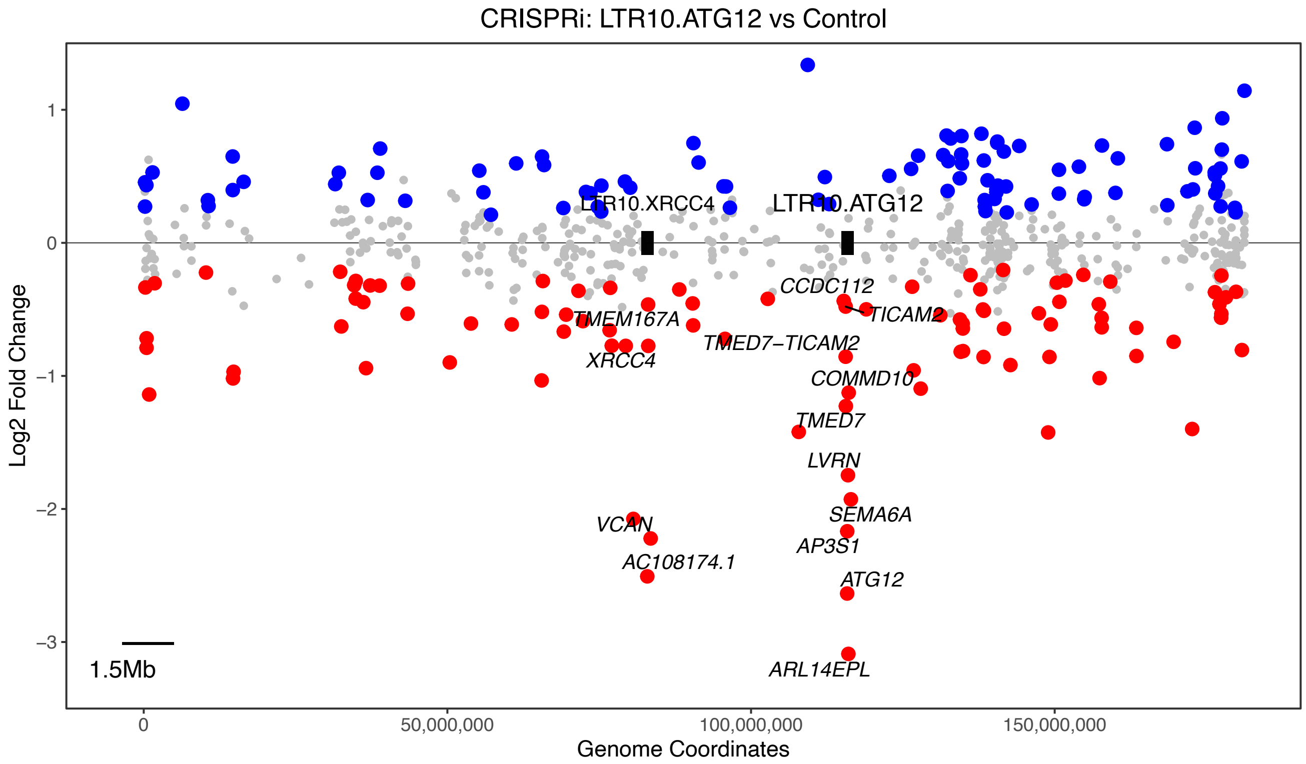

Fig. S56. Scatterplot of gene expression changes in response to silencing LTR10.ATG12, showing all of hg38 chromosome 5. The locations of LTR10.XRCC4 and LTR10.ATG12 are marked (element boxes not drawn to scale). Significantly downregulated genes are shown in red; significantly upregulated genes are shown in blue. Significantly downregulated genes within 1.5 Mb of either LTR10 element are labelled.

Fig. S57.

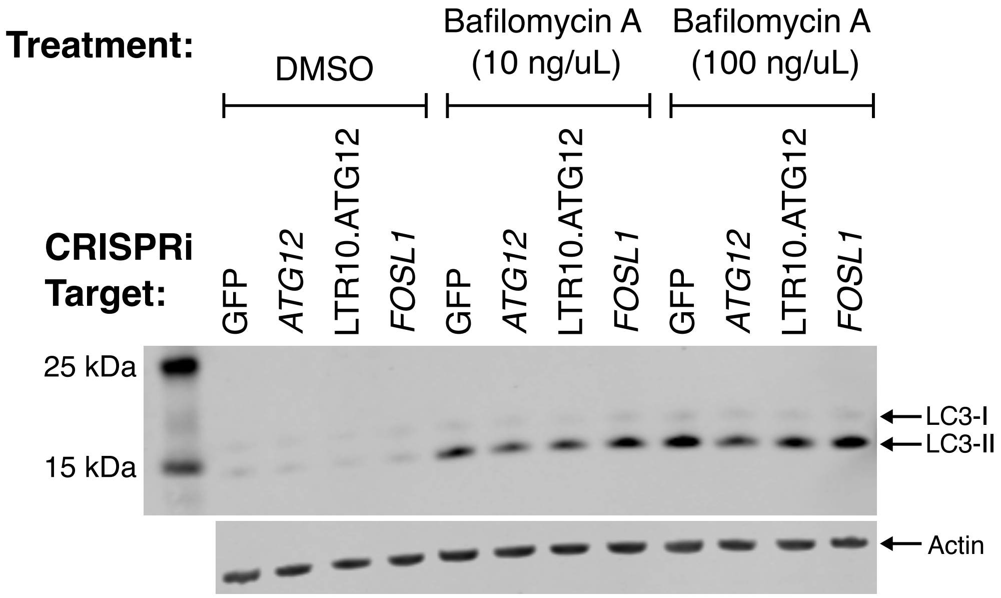

Fig. S57. Immunoblot of LC3-I and LC3-II in each CRISPRi cell line, after treating cells for 6 hr with DMSO, bafilomycin A (10 ng/uL), or bafilomycin A (100 ng/uL).

Fig. S58.

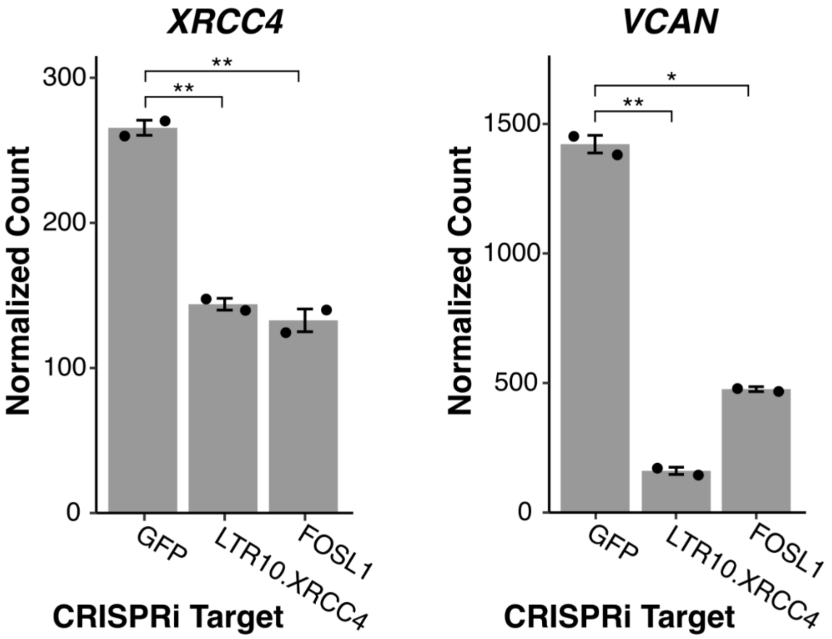

Fig. S58. Normalized RNA-seq expression values of *XRCC4* and *VCAN* in dCas9-KRAB-MeCP2 HCT116 cells stably transfected with gRNAs targeting the LTR10.XRCC4 element, the *FOSL1* transcription start site, or non-targeting (GFP) control.

Fig. S59.

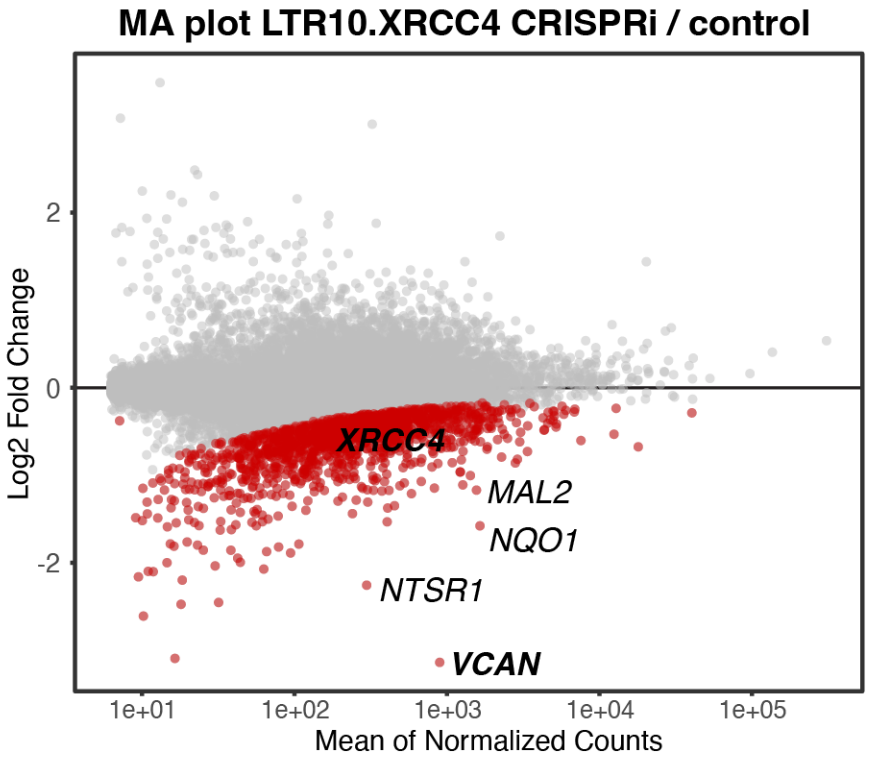

Fig. S59. MA plot showing global gene expression changes in cells in response to silencing LTR10.XRCC4. Significantly downregulated genes are shown in red.

Fig. S60.

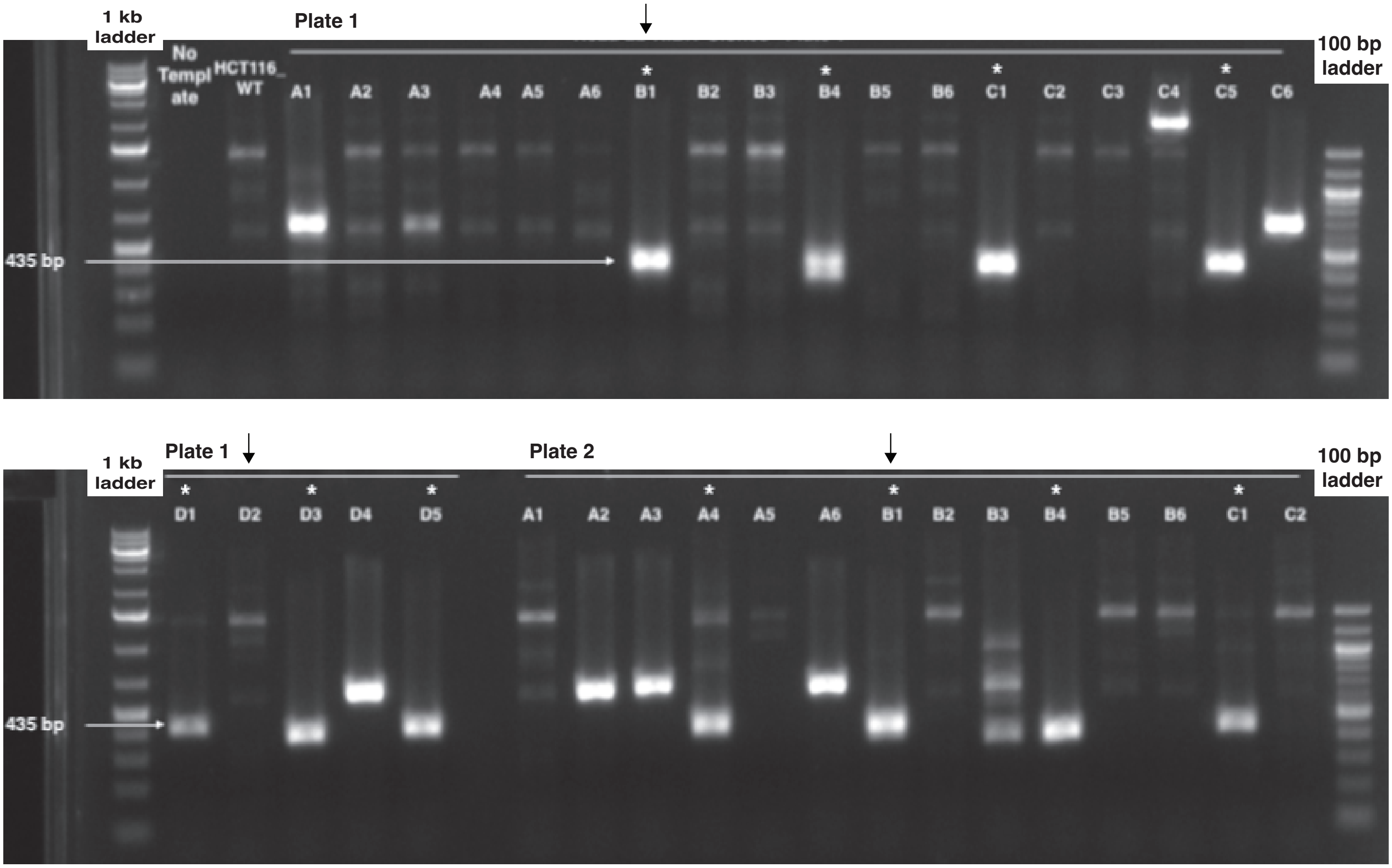

Fig. S60. External PCR validation of LTR10.XRCC4 CRISPR KO clones. PCR primers used for validation (XRCC4_up_external, XRCC4_down_external) flank LTR10.XRCC4 enhancer. Expected KO amplicon size is 435 bp. Expected wildtype amplicon size is 7763 bp. Asterisks indicate clones with 435 bp dominant product, indicative of a KO. Several clones showed no product at 435 bp and were propagated to serve as negative controls. Arrows indicate clones that were used for irradiation and mouse xenograft experiments: wildtype D2 and knockout B1.

Fig. S61.

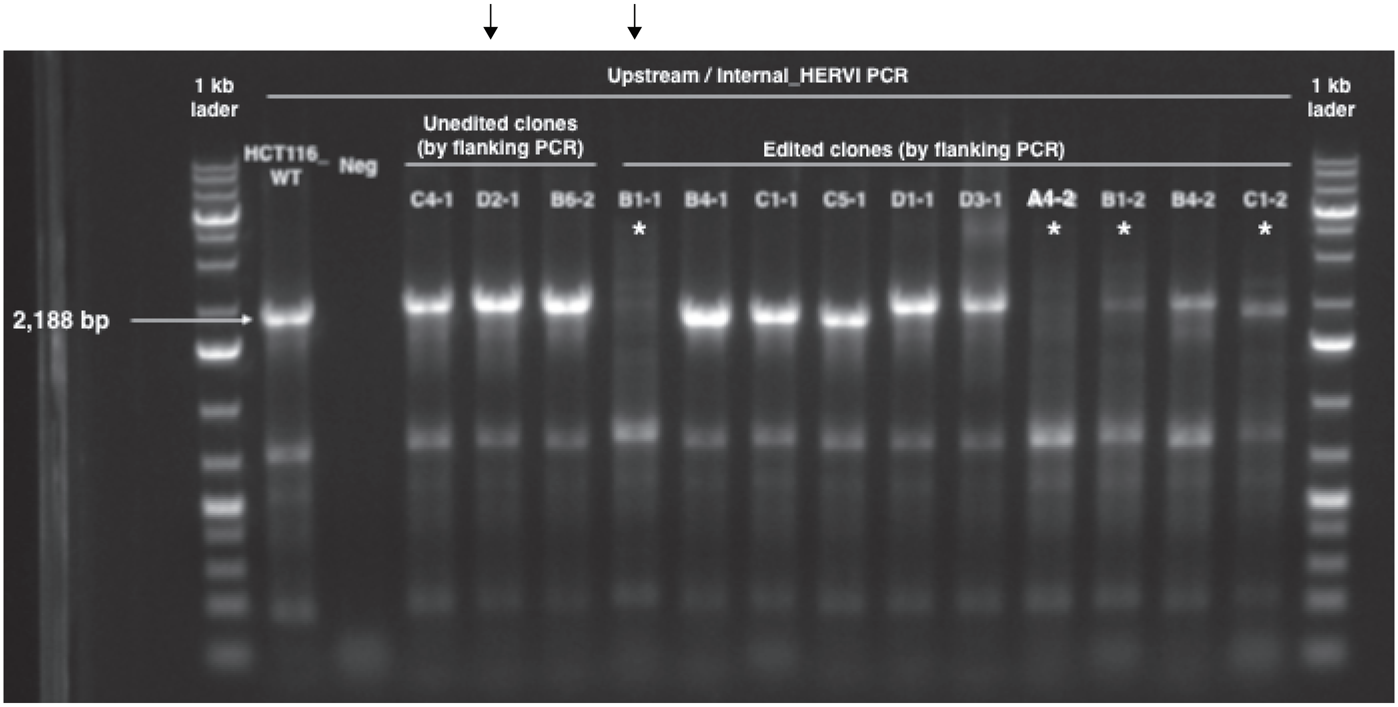

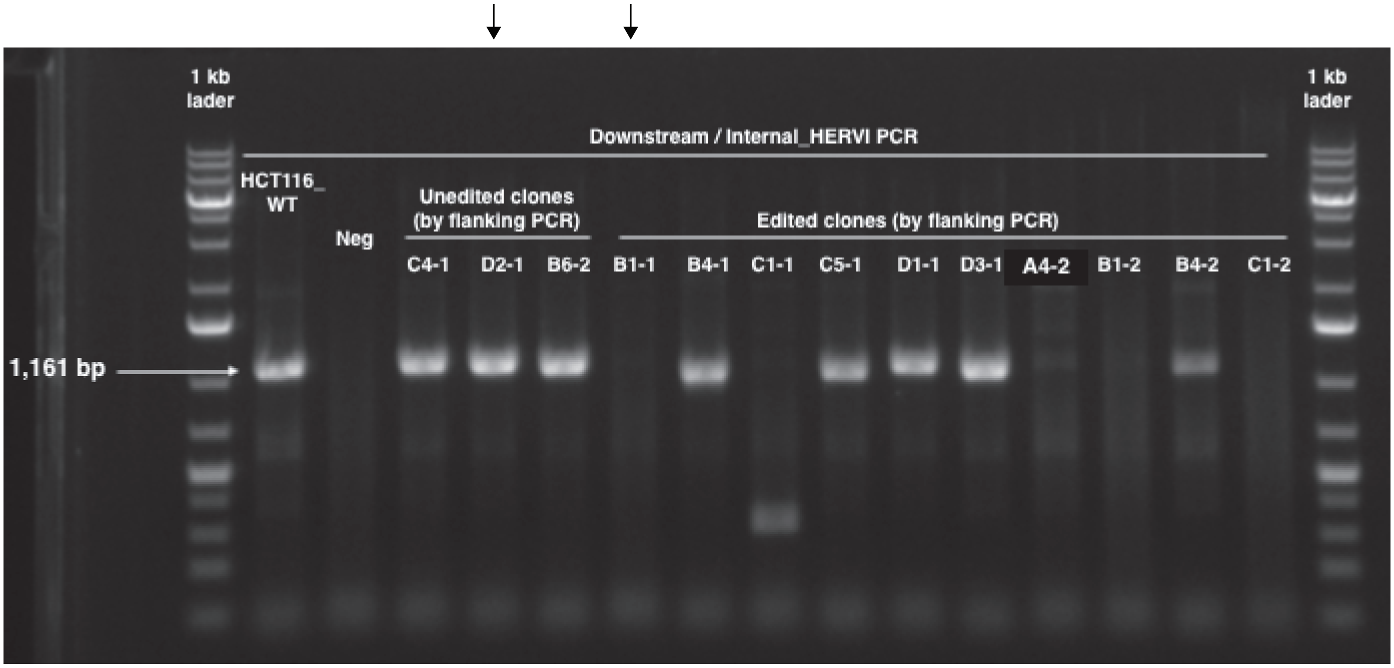

Fig. S61. Internal PCR validation of LTR10.XRCC4 CRISPR KO clones. Expected wildtype amplicon size is 2188 bp for upstream PCR, 1161 bp for downstream PCR. Asterisks indicate KO clones. Clones B1-1 and A4-2 showed no product in both reactions, indicative of homozygous KO clones. Clones B1-2 and C1-2 lacked product in the downstream reaction but showed a faint product in the upstream reaction. Arrows indicate clones that were used for irradiation and mouse xenograft experiments: wildtype D2-1 and knockout B1-1.

Fig. S62.

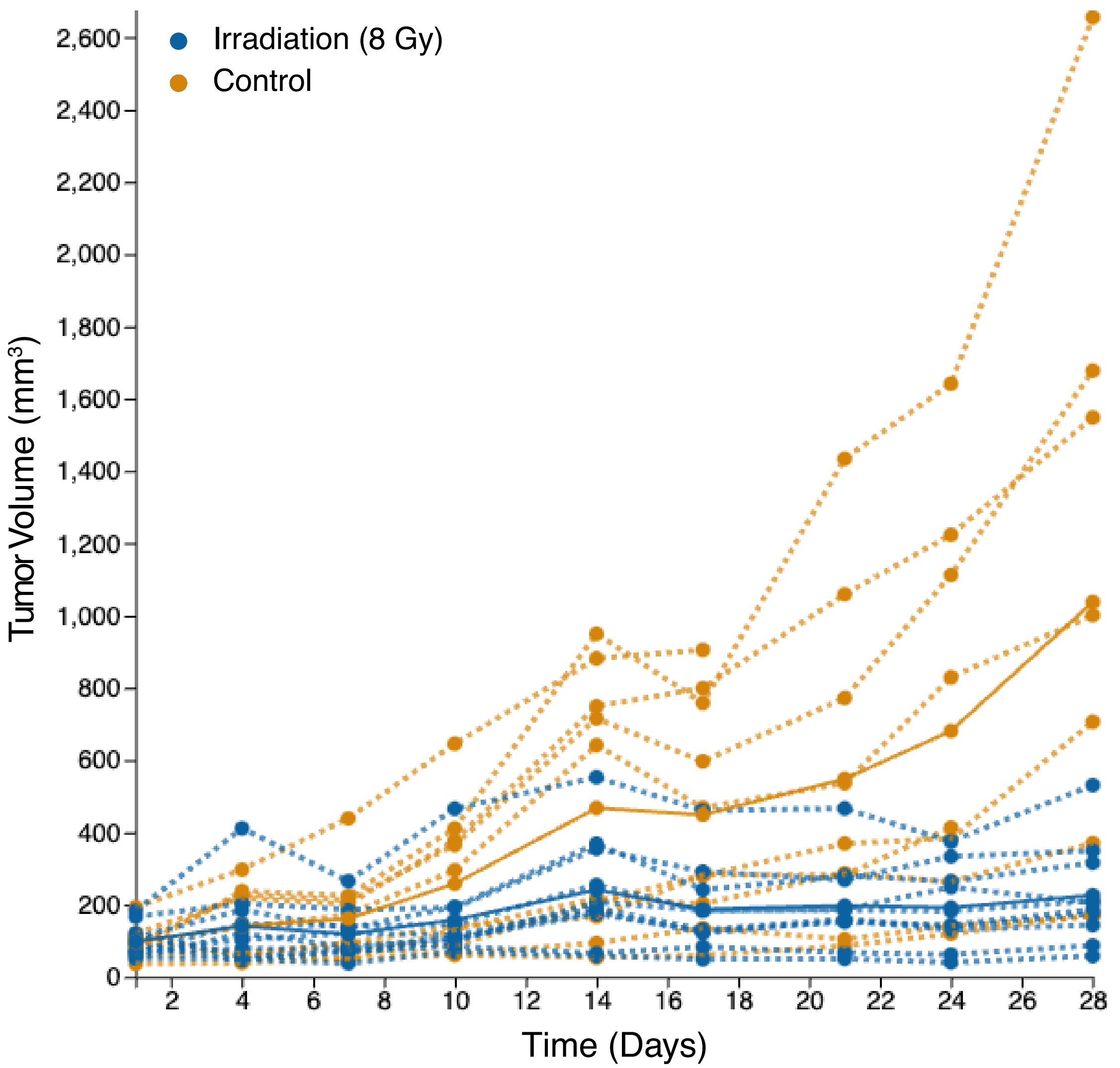

Fig. S62. Individual growth curves across replicates for wildtype HCT116 xenograft tumors, with irradiation (n=10) and without irradiation (n=10), for 28 days.

Fig. S63.

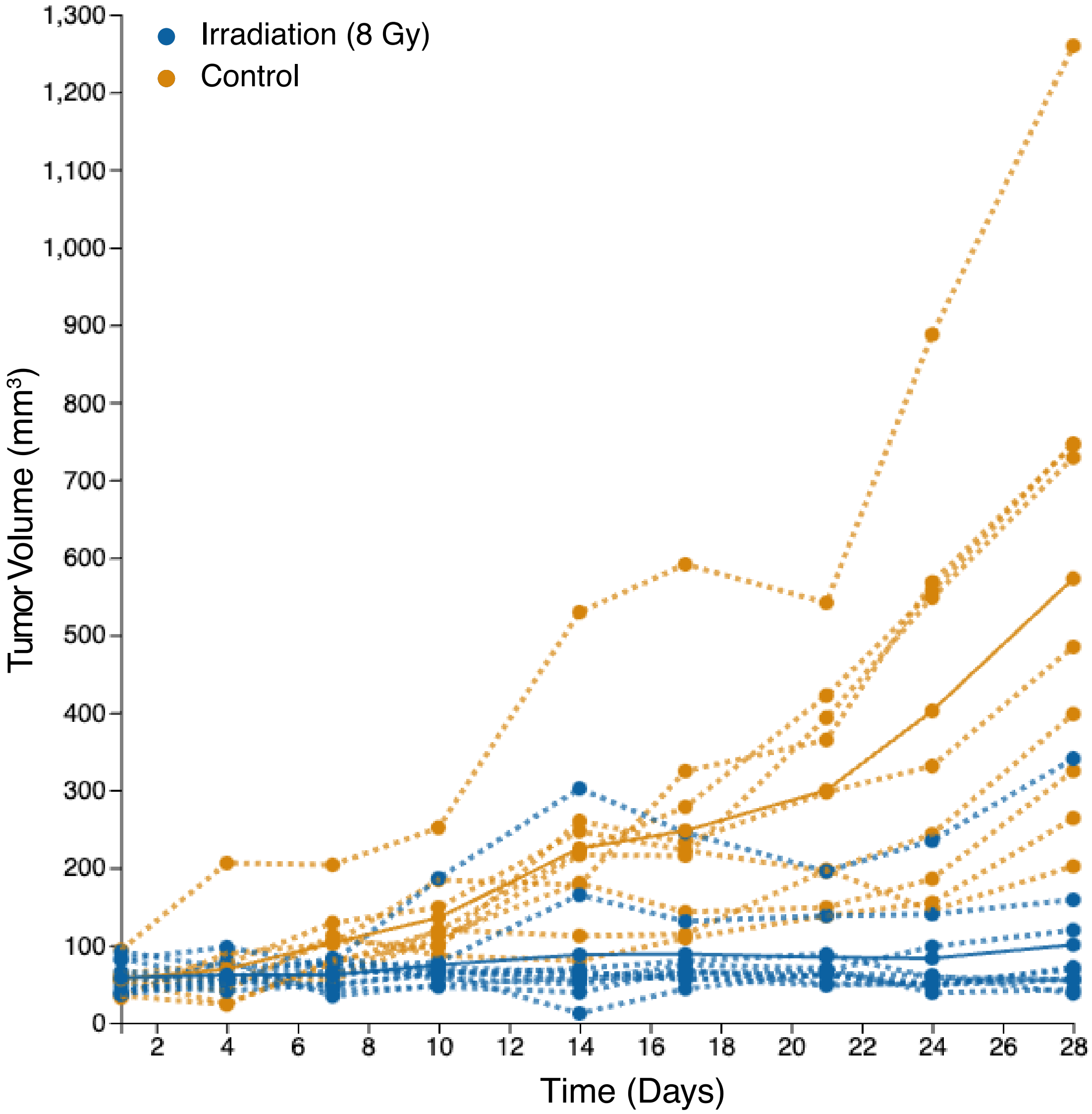

Fig. S63. Individual growth curves across replicates for LTR10.XRCC4 knockout HCT116 xenograft tumors, with irradiation (n=10) and without irradiation (n=9), for 28 days.

Fig. S64.

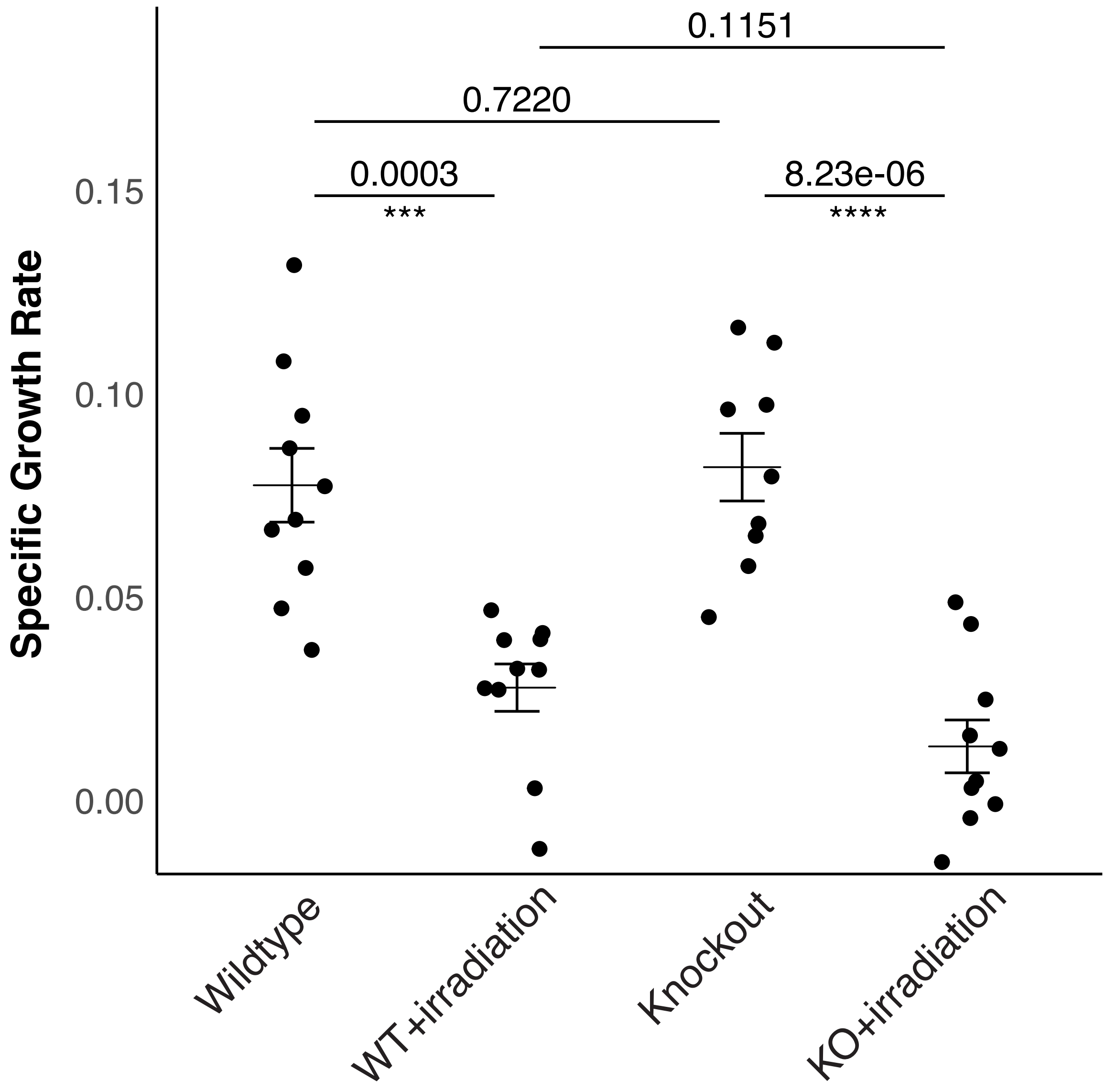

Fig. S64. Specific growth rates for each HCT116 xenograft tumor, separated by group: wildtype (n=10), wildtype + irradiation (n=10), LTR10.XRCC4 knockout (n=9), and LTR10.XRCC4 knockout + irradiation (n=10). Welch's t-test p-values are shown for each comparison.

Fig. S65.

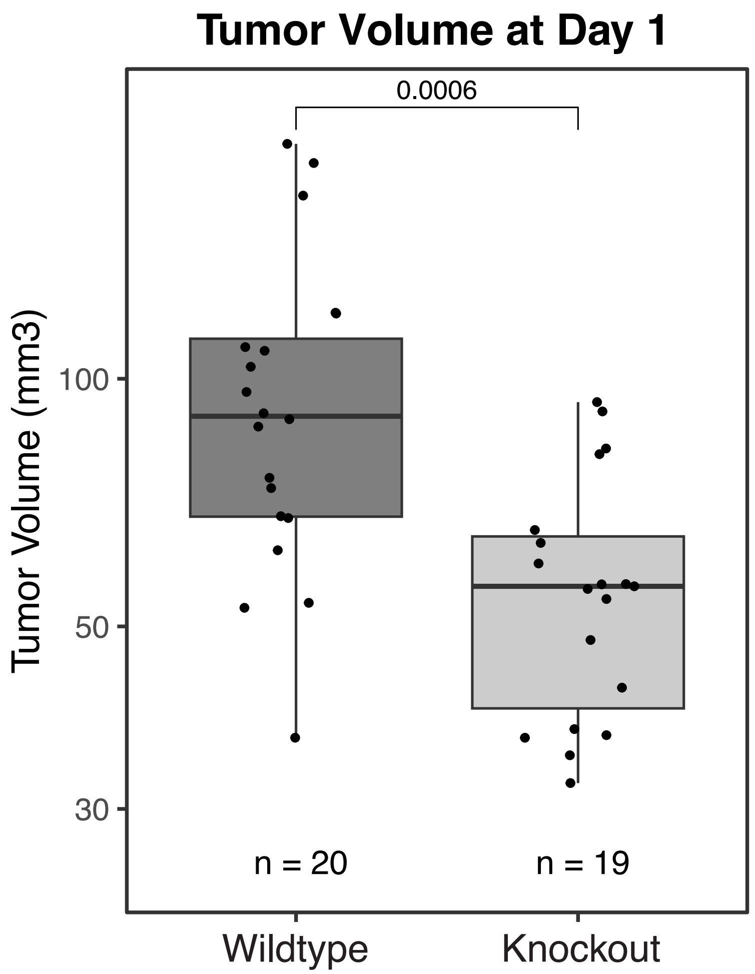

Fig. S65. Boxplot comparing tumor sizes at day 1 of irradiation for each HCT116 xenograft tumor, separated by group: wildtype (n=20) versus LTR10.XRCC4 knockout (n=19). Welch's t-test p-value is shown.

Fig. S66.

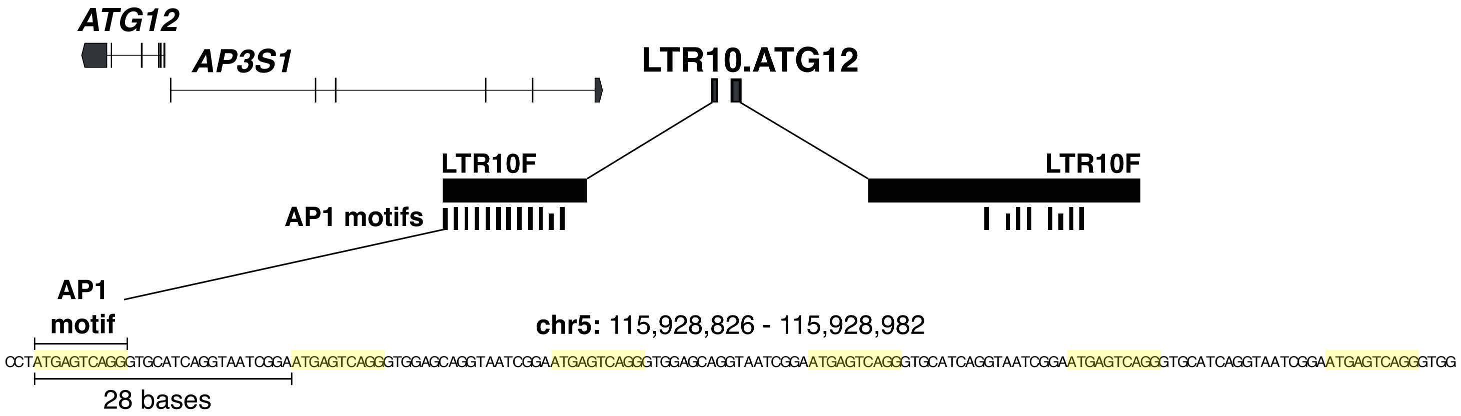

Fig. S66. Genome browser screenshot of LTR10.ATG12 showing a close-up of the 28 bp tandem repeat region that includes the AP1 motif.

Fig. S67.

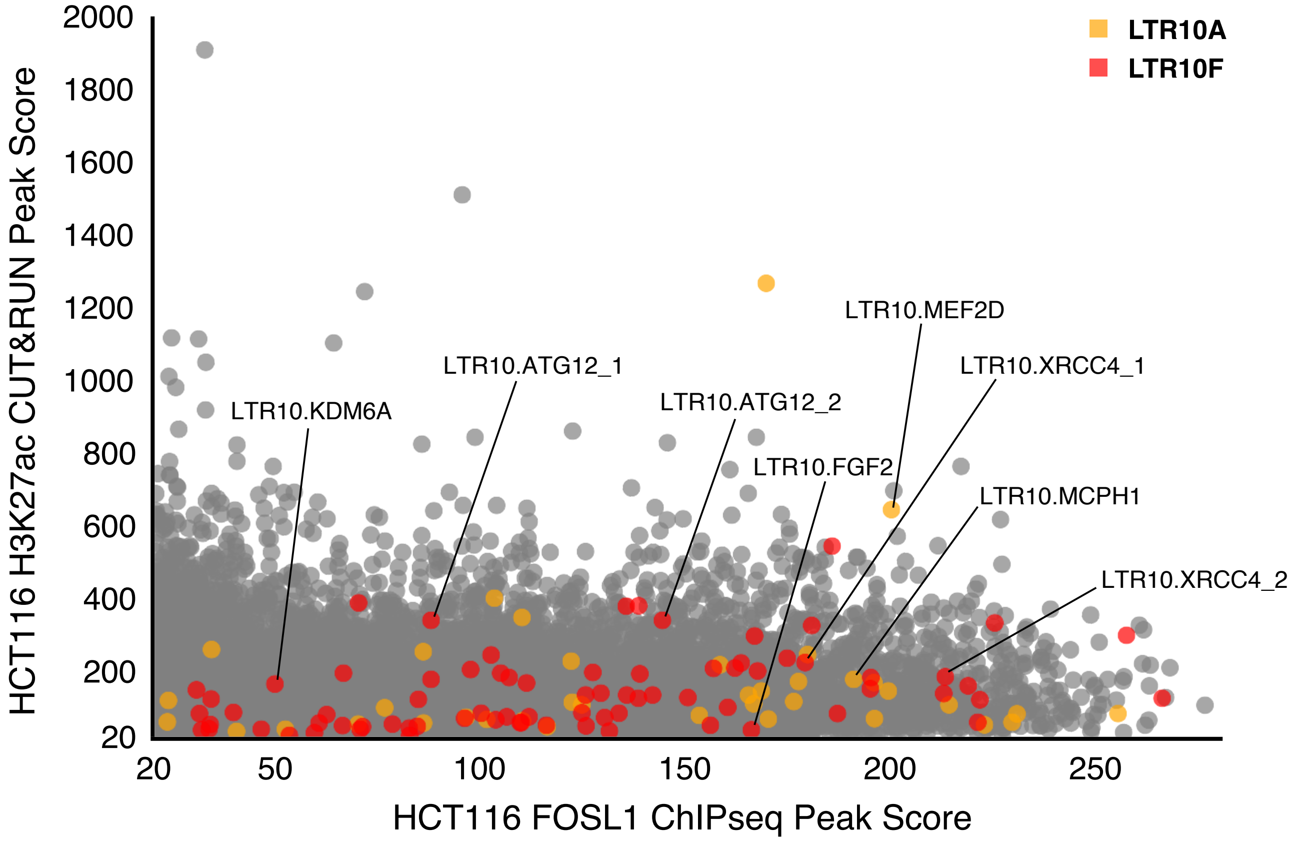

Fig. S67. Scatterplot of H3K27ac and FOSL1 peak scores for all HCT116 peak regions that have both marks. HCT116 H3K27ac peaks (in-house CUT&RUN, filtered for peak score over 20) were intersected with HCT116 FOSL1 peaks (public ChIP-seq from GSE32465, filtered for peak score over 20) to define a list of genomic peaks with both marks (n=19,315). LTR10A/F elements were then intersected with these H3K27ac+FOSL1 peak regions. 143 distinct LTR10A/F elements contribute to 115 of these peaks. LTR10A-derived peaks are shown in orange, LTR10F-derived peaks are shown in red, and all other genomic H3K27ac+FOSL1 peaks are shown in grey. CRISPR-validated LTR10 enhancers are labeled by name.

Fig. S68.

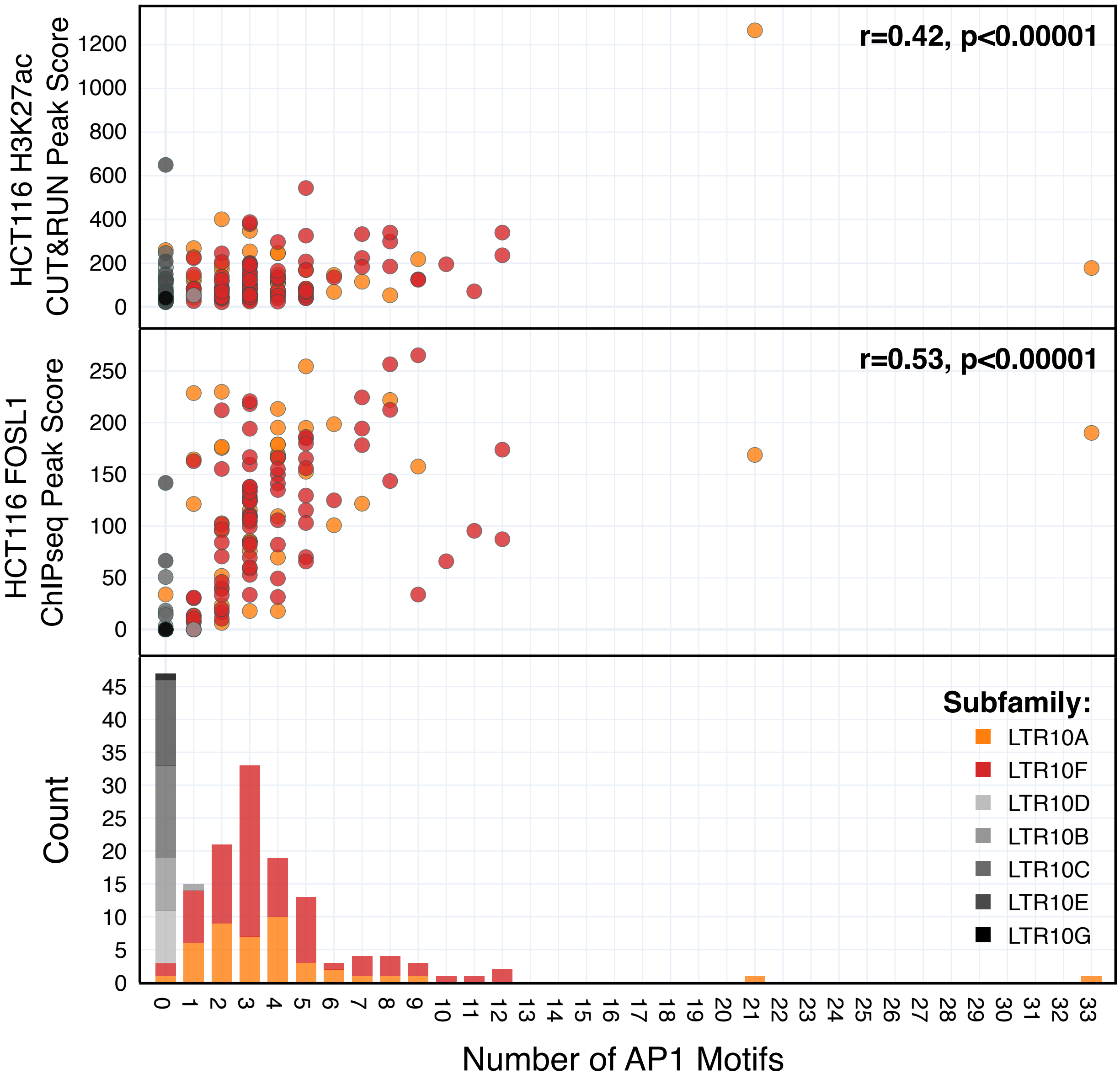

Fig. S68. Scatterplots showing correlations between the number of AP1 motifs with HCT116 H3K27ac signal (in-house CUT&RUN) and HCT116 FOSL1 signal (public ChIP-seq from GSE32465). Only LTR10 elements marked by H3K27ac are included. The Pearson correlation coefficient (r) and p-value are shown for each plot.

Fig. S69.

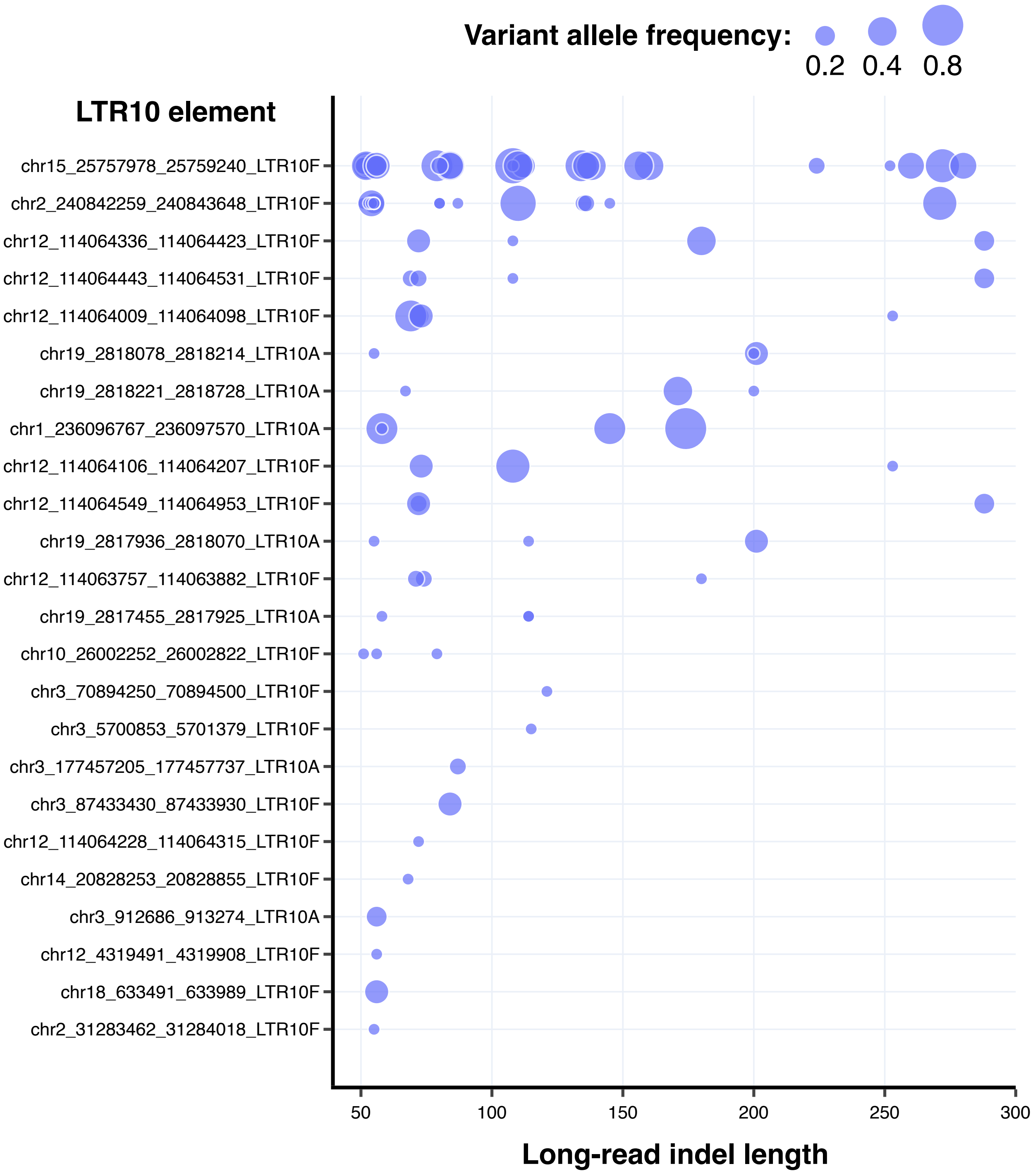

Fig. S69. Scatterplot of long-read indels between 50-300 bp in length overlapping LTR10A or LTR10F elements. Each dot represents a distinct indel, plotted by its length and variant allele frequency (bubble size). Indels were derived from long-read structural variant calls generated from 15 individuals *(77)*.

Fig. S70.

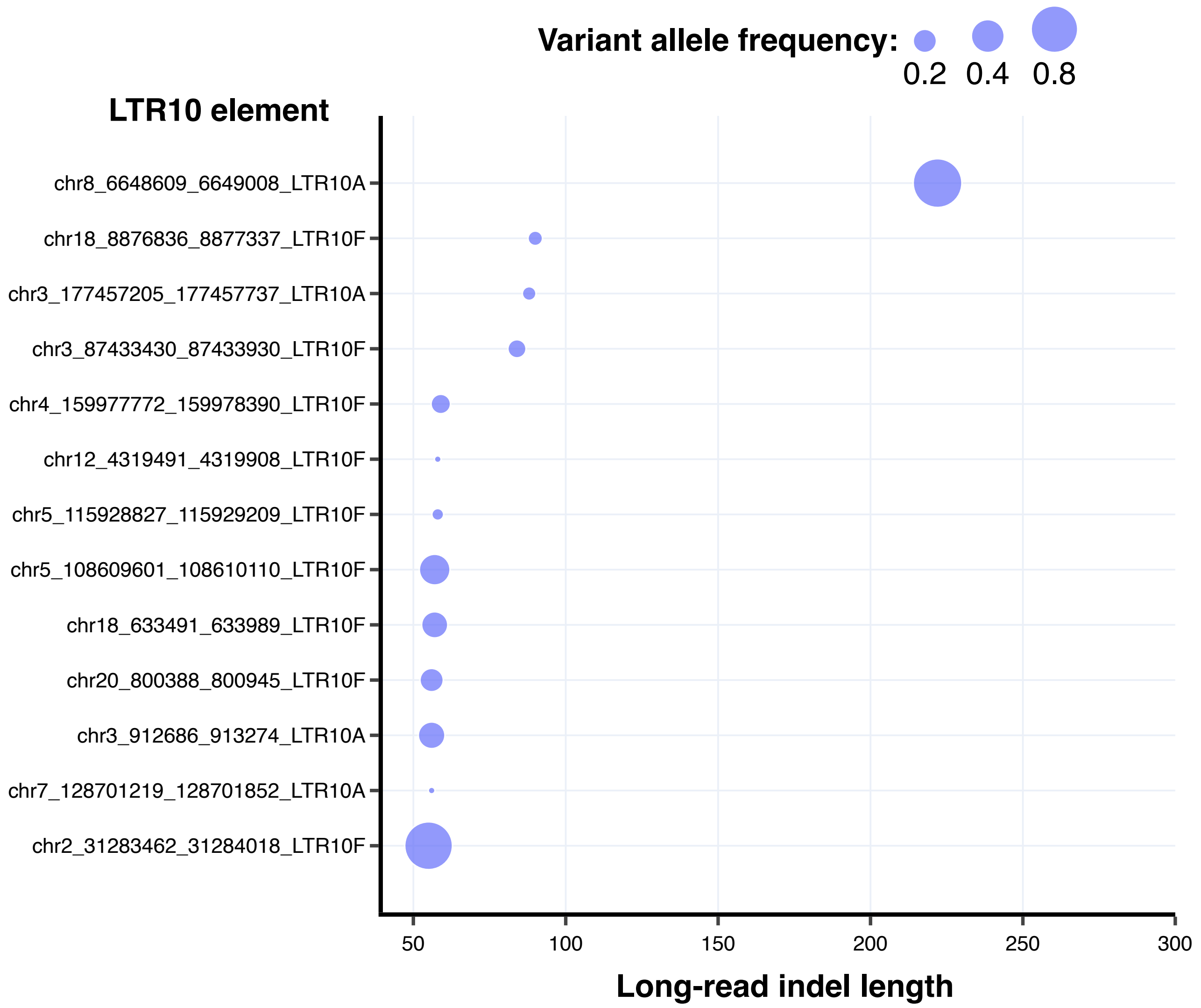

Fig. S70. Scatterplot of long-read indels between 50-300 bp in length overlapping LTR10A or LTR10F elements. Each dot represents a distinct indel, plotted by its length and variant allele frequency (bubble size). Indels were derived from long-read structural variant calls generated from 25 individuals *(78)*.

Fig. S71.

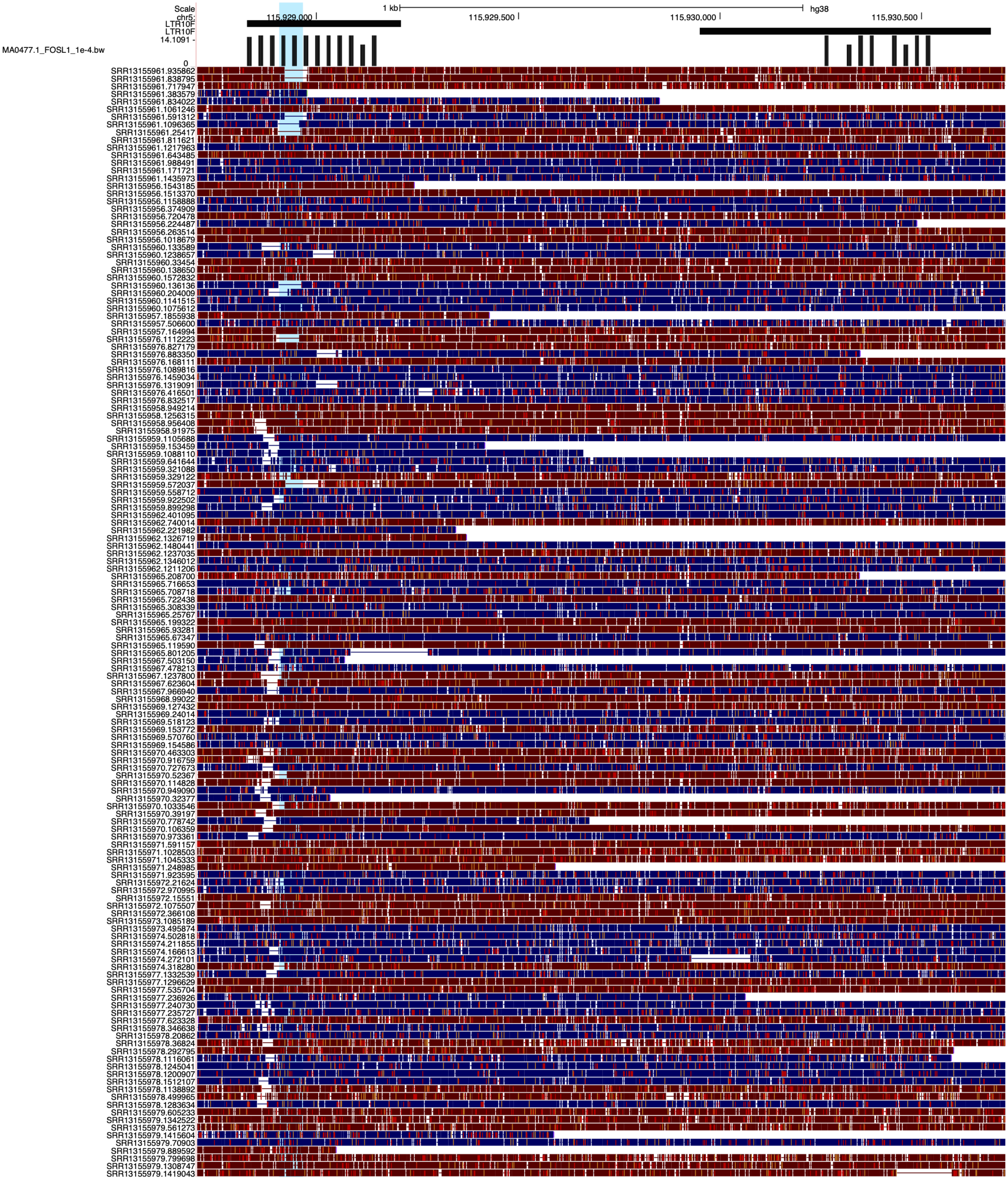

Fig. S71. Genome browser screenshot of a deletion within the LTR10.ATG12 element, showing reads from a long-read dataset of 25 Asian individuals *(78)*. The blue highlight shows the location of the reported deletion.

Fig. S72.

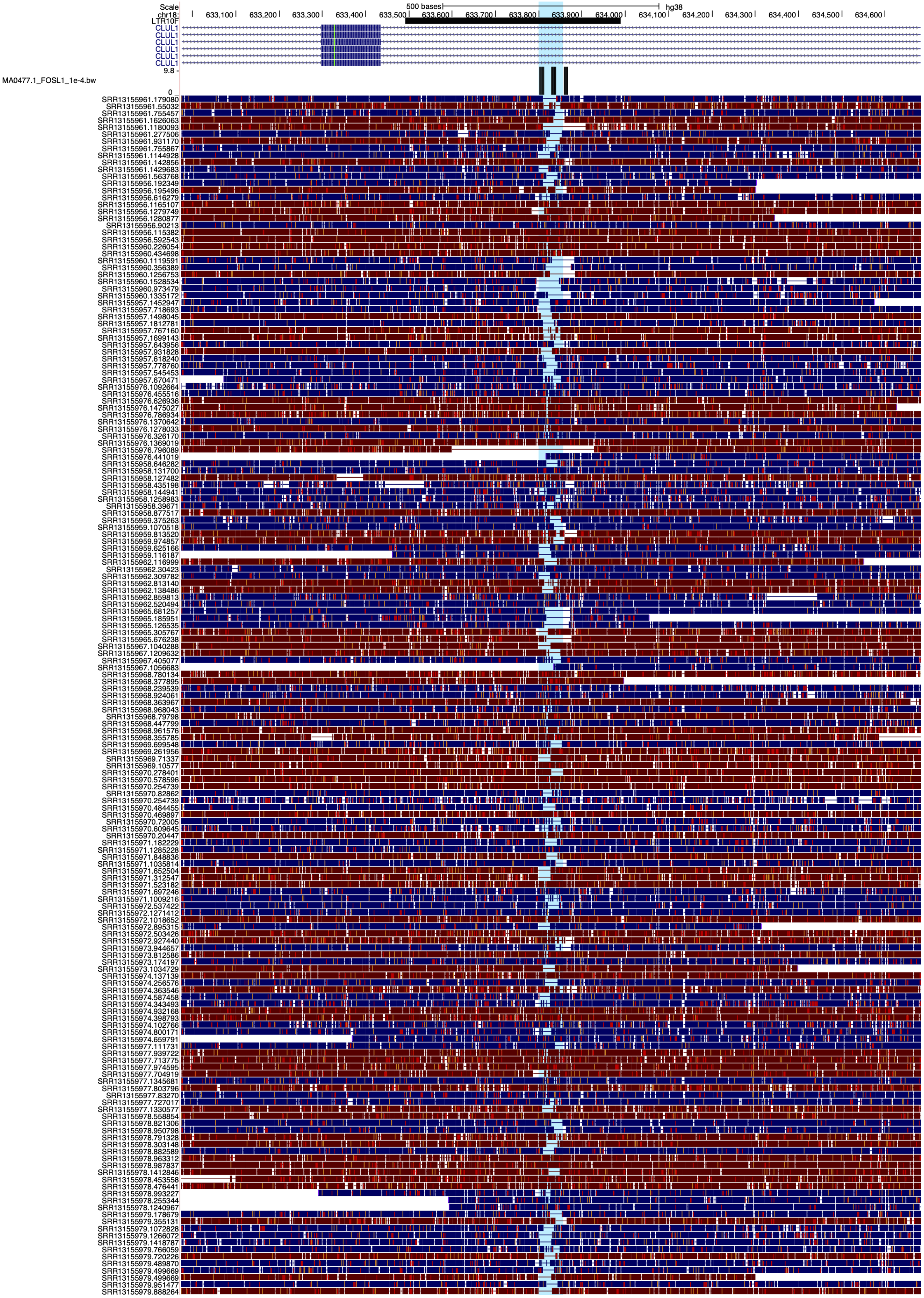

Fig. S72. Genome browser screenshot of a deletion within an LTR10F element within the CLUL1 gene, showing reads from a long-read dataset of 25 Asian individuals *(78)*. The blue highlight shows the location of the reported deletion.

Fig. S73.

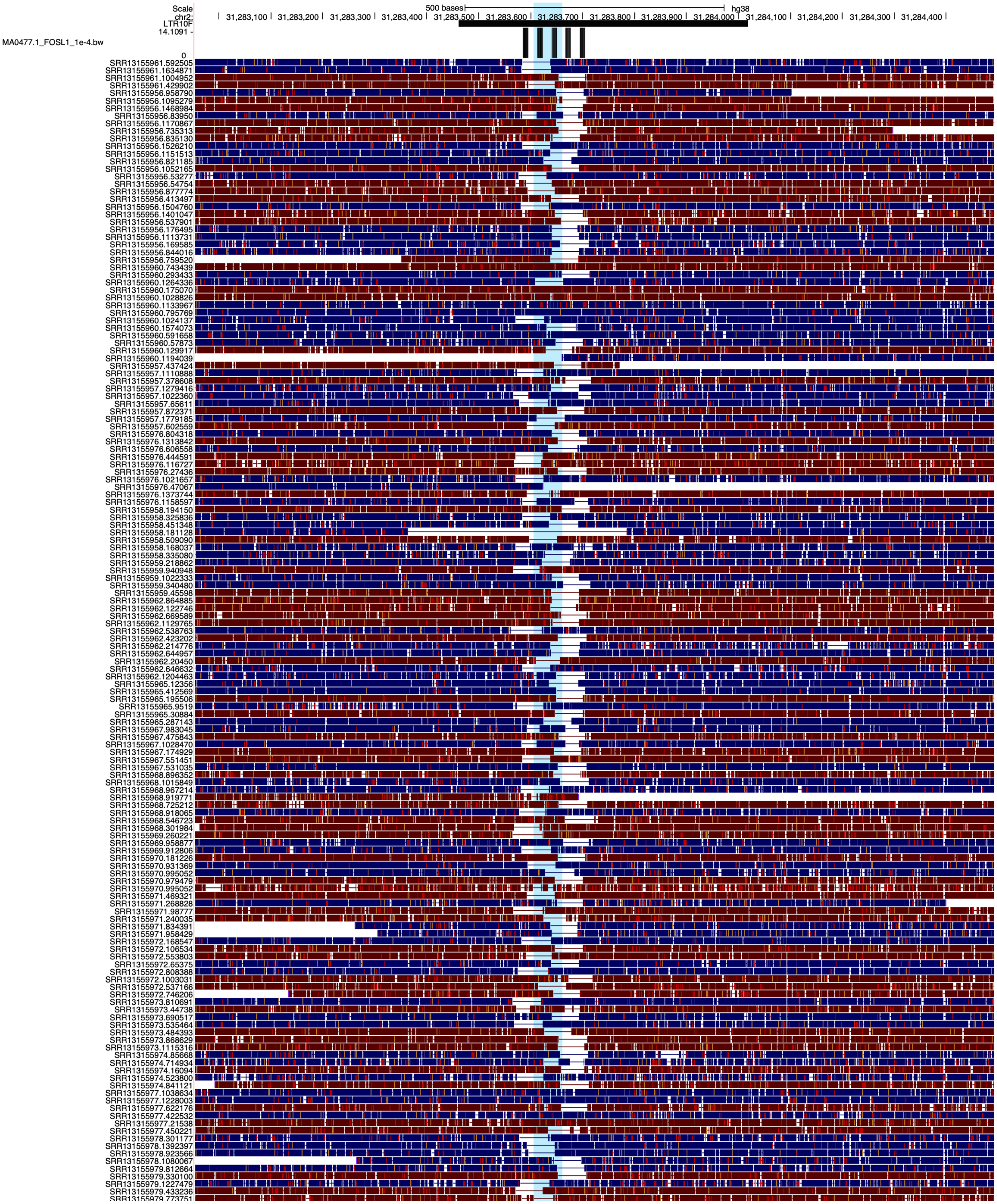

Fig. S73. Genome browser screenshot of a chr2 deletion within an LTR10F element, showing reads from a long-read dataset of 25 Asian individuals *(78)*. The blue highlight shows the location of the reported deletion.

Fig. S74.

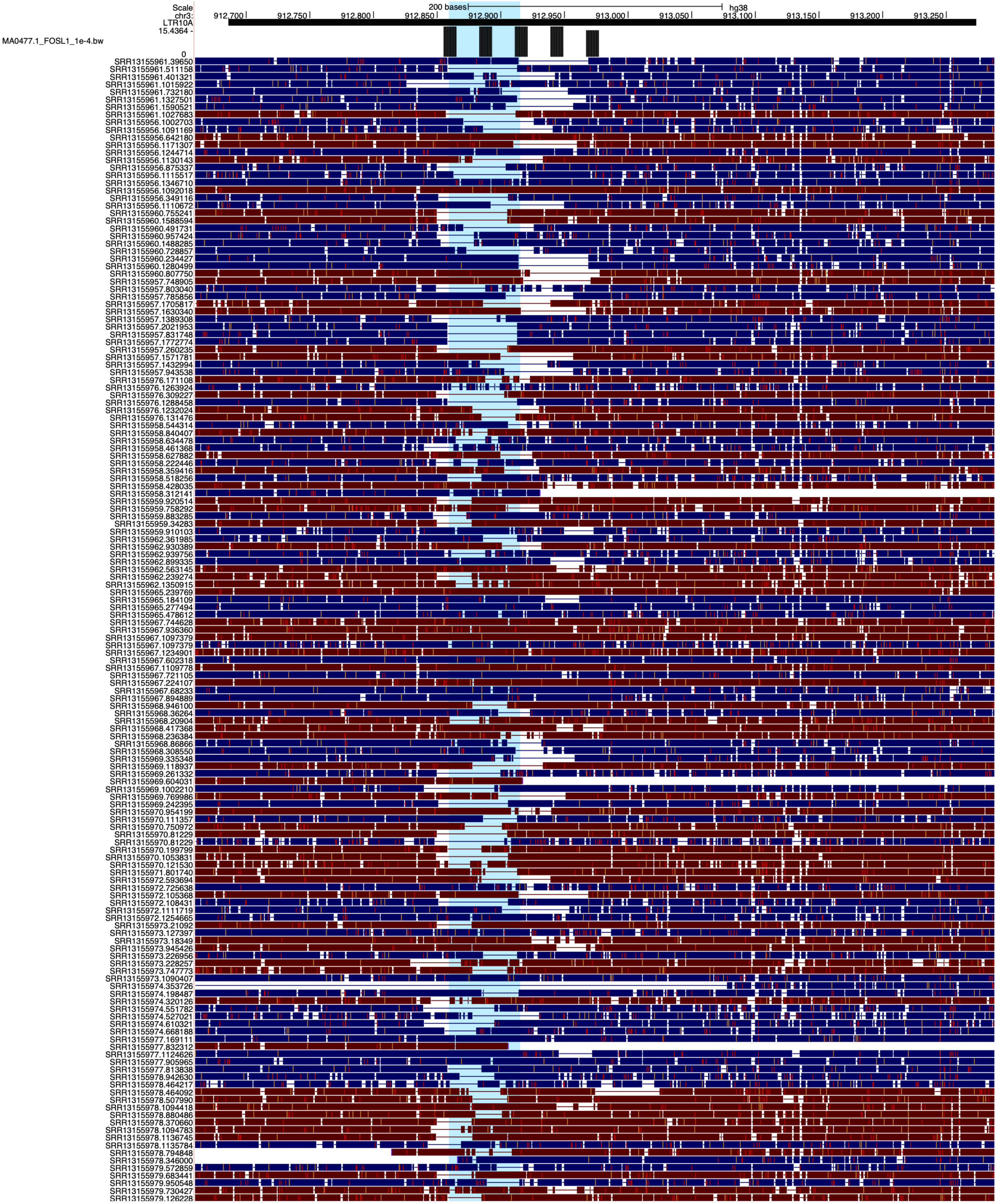

Fig. S74. Genome browser screenshot of a chr3 deletion within an LTR10A element, showing reads from a long-read dataset of 25 Asian individuals *(78)*. The blue highlight shows the location of the reported deletion.

Fig. S75.

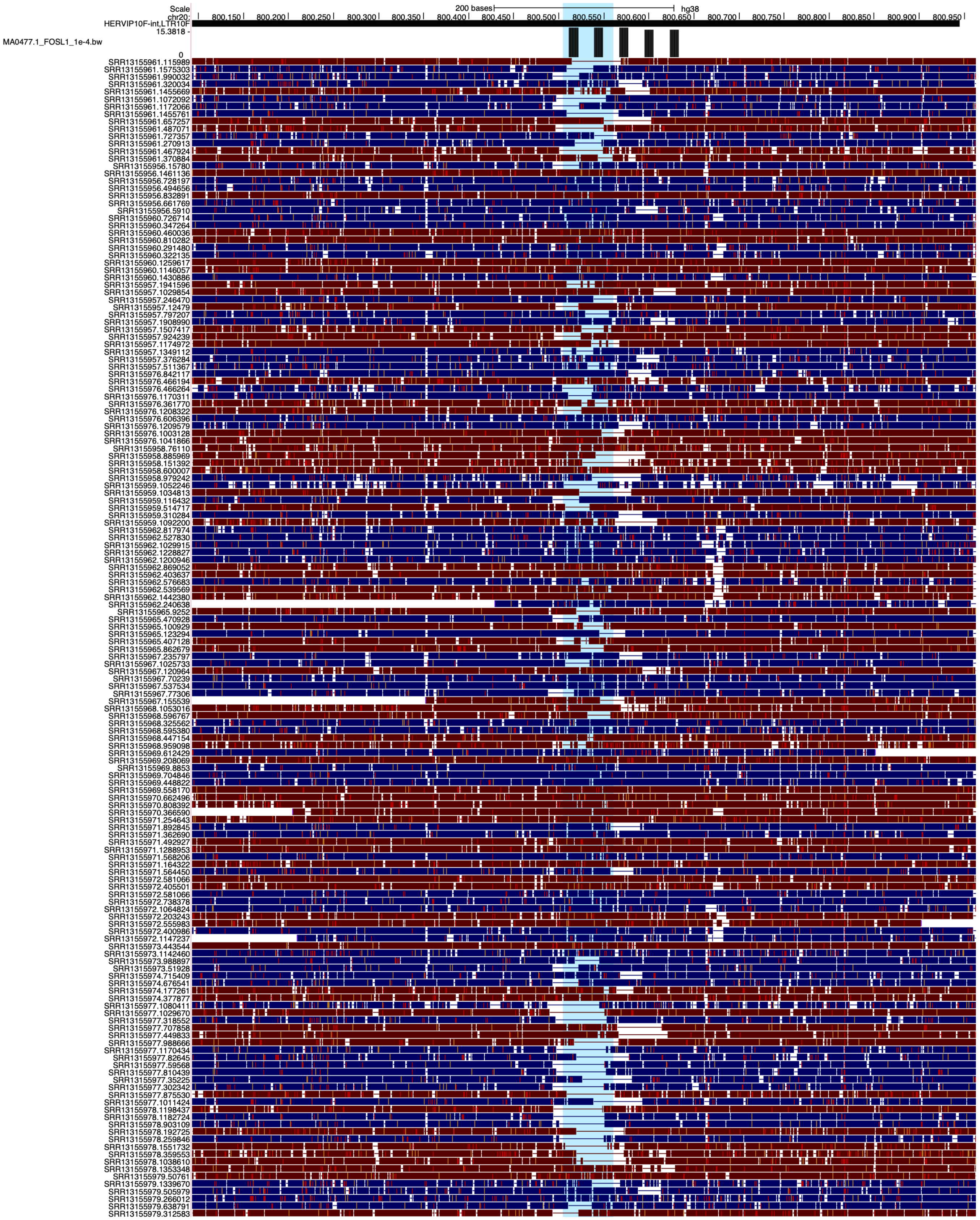

Fig. S75. Genome browser screenshot of a chr20 deletion within an LTR10F element, showing reads from a long-read dataset of 25 Asian individuals *(78)*. The blue highlight shows the location of the reported deletion.

Fig. S76.

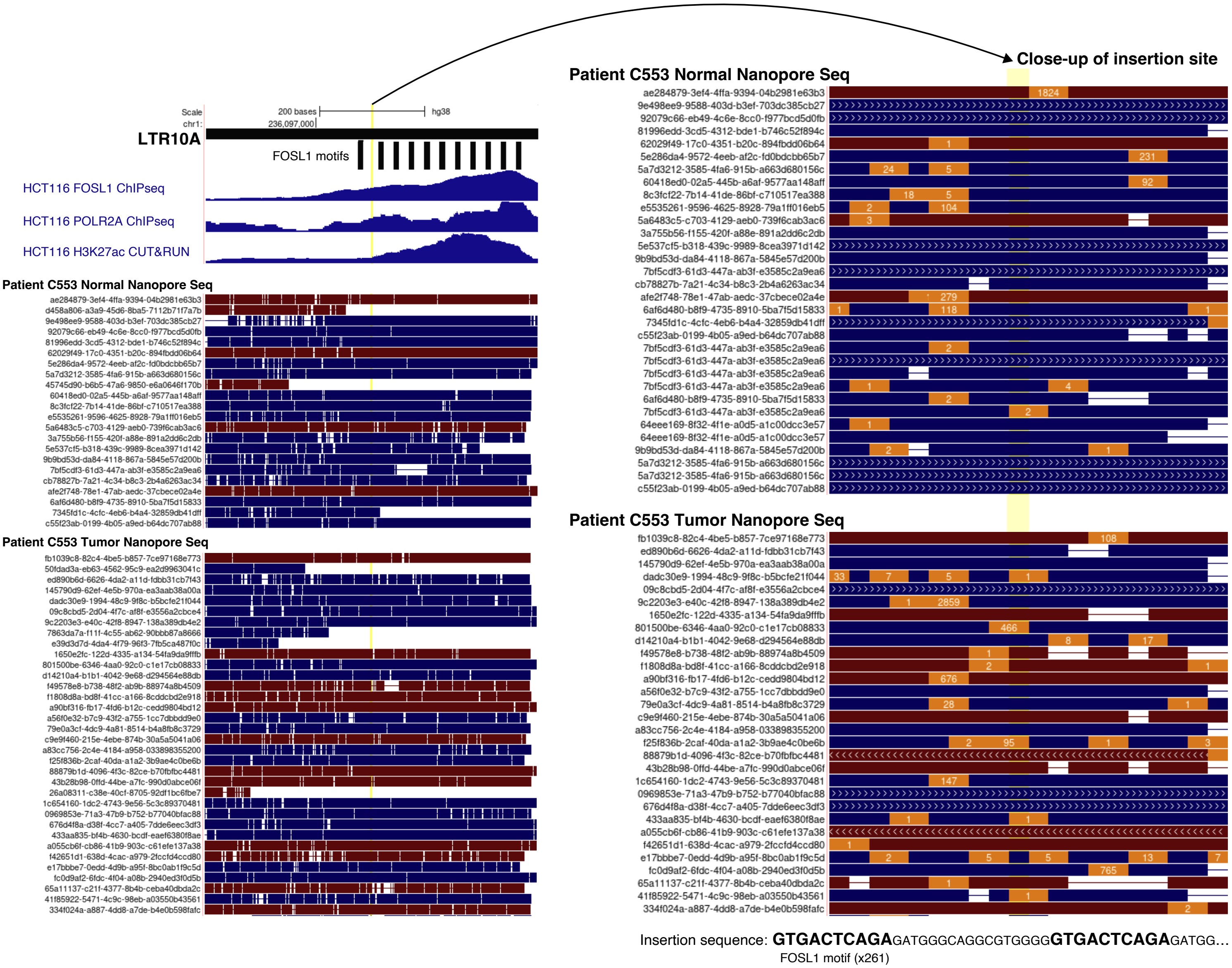

Fig. S76. Genome browser screenshots of a tumor-specific LTR10A VNTR expansion on chr1. Matched tumor/normal reads from patient C553 are derived from a long-read nanopore sequencing dataset of 20 patients with colorectal adenocarcinomas *(80)*. The yellow highlight shows the approximate location of the predicted tumor-specific mosaic expansion, as called by Sniffles2 *(81)*. A close-up of reads at the insertion site is shown on the right, with orange boxes indicating insertions and numbers within orange boxes indicating insertion sequence lengths (in bp).

Fig. S77.

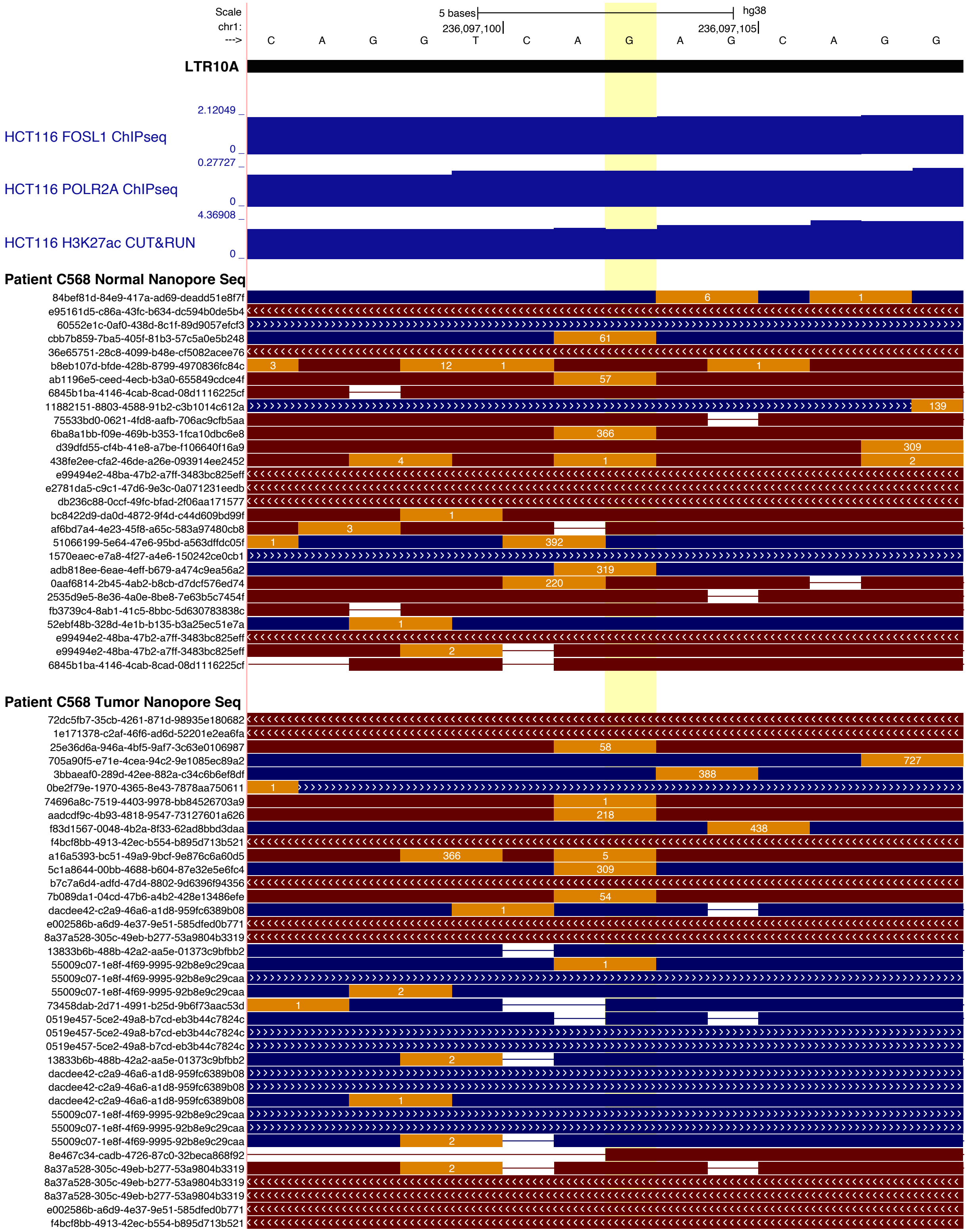

Fig. S77. Genome browser screenshot of a tumor-specific LTR10A VNTR expansion on chr1. Matched tumor/normal reads from patient C568 are derived from a long-read nanopore sequencing dataset of 20 patients with colorectal adenocarcinomas *(80)*. The yellow highlight shows the approximate location of the predicted tumor-specific mosaic expansion, as called by Sniffles2 *(81)*. Orange boxes indicate insertions and numbers within orange boxes indicate insertion sequence lengths (in bp).

Fig. S78.

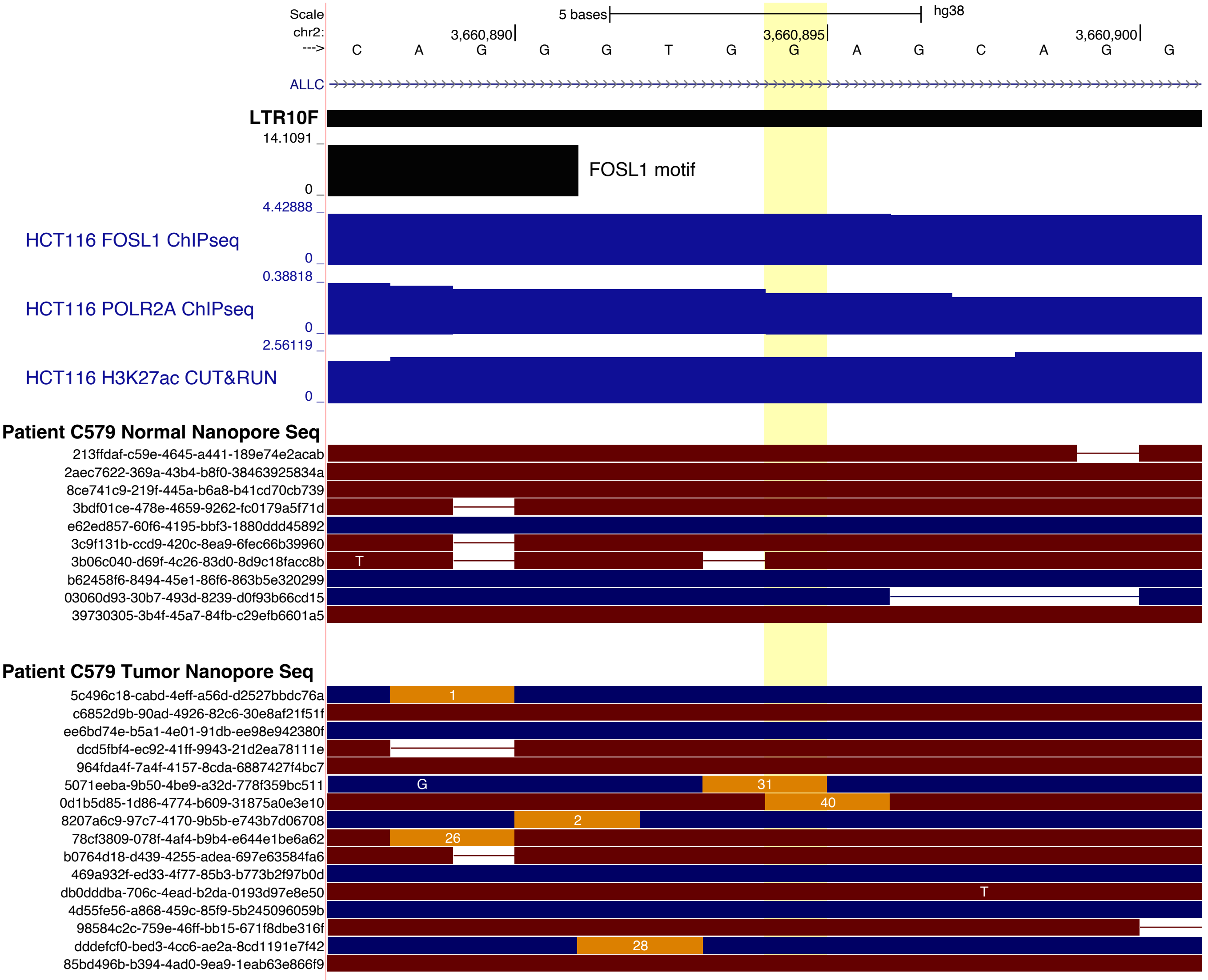

Fig. S78. Genome browser screenshot of a tumor-specific LTR10F VNTR expansion on chr2. Matched tumor/normal reads from patient C579 are derived from a long-read nanopore sequencing dataset of 20 patients with colorectal adenocarcinomas *(80)*. The yellow highlight shows the approximate location of the predicted tumor-specific mosaic expansion, as called by Sniffles2 *(81)*. Orange boxes indicate insertions and numbers within orange boxes indicate insertion sequence lengths (in bp).

Fig. S79.

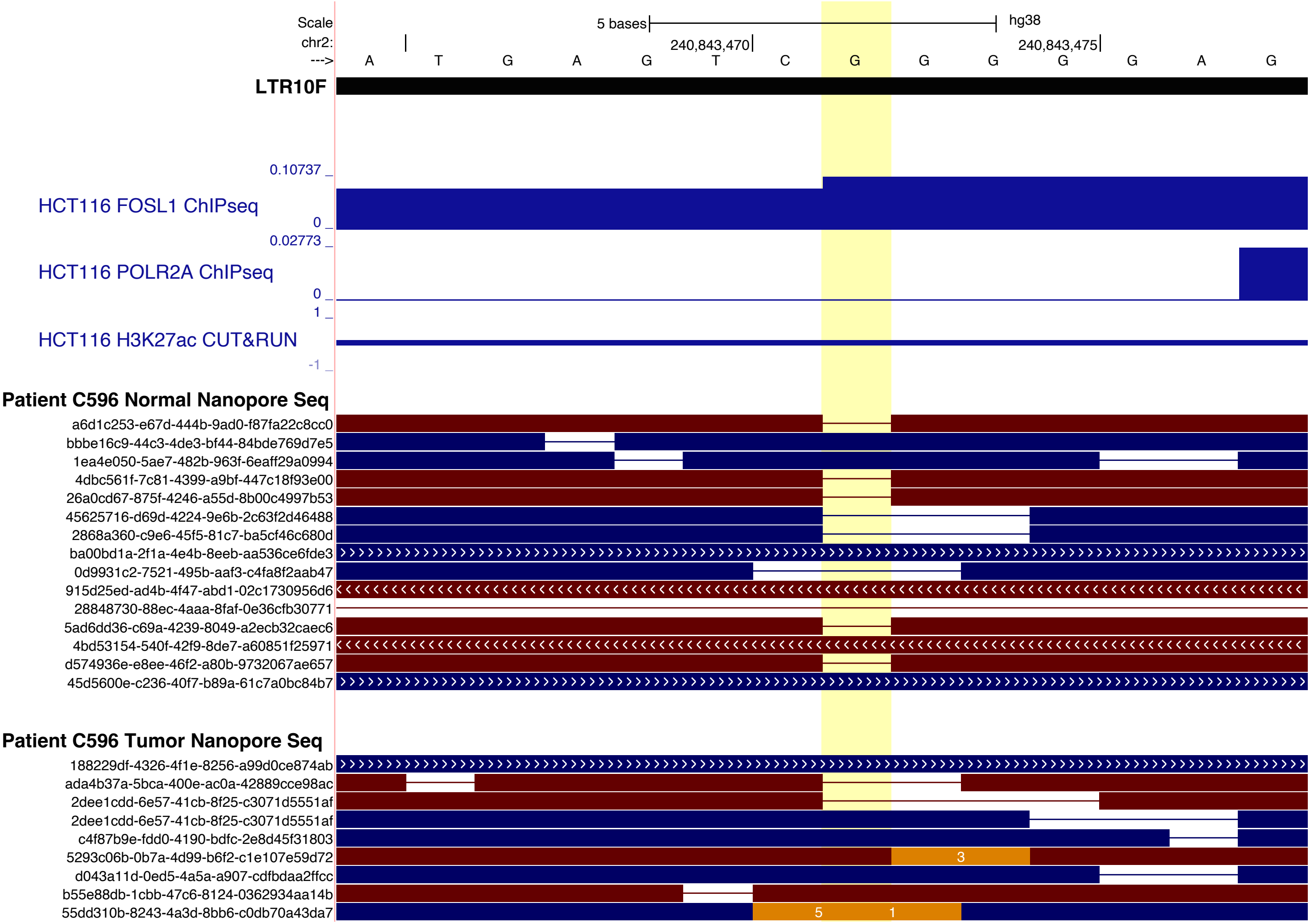

Fig. S79. Genome browser screenshot of a tumor-specific LTR10F VNTR expansion on chr2. Matched tumor/normal reads from patient C596 are derived from a long-read nanopore sequencing dataset of 20 patients with colorectal adenocarcinomas *(80)*. The yellow highlight shows the approximate location of the predicted tumor-specific mosaic expansion, as called by Sniffles2 *(81)*. Orange boxes indicate insertions and numbers within orange boxes indicate insertion sequence lengths (in bp).

Fig. S80.

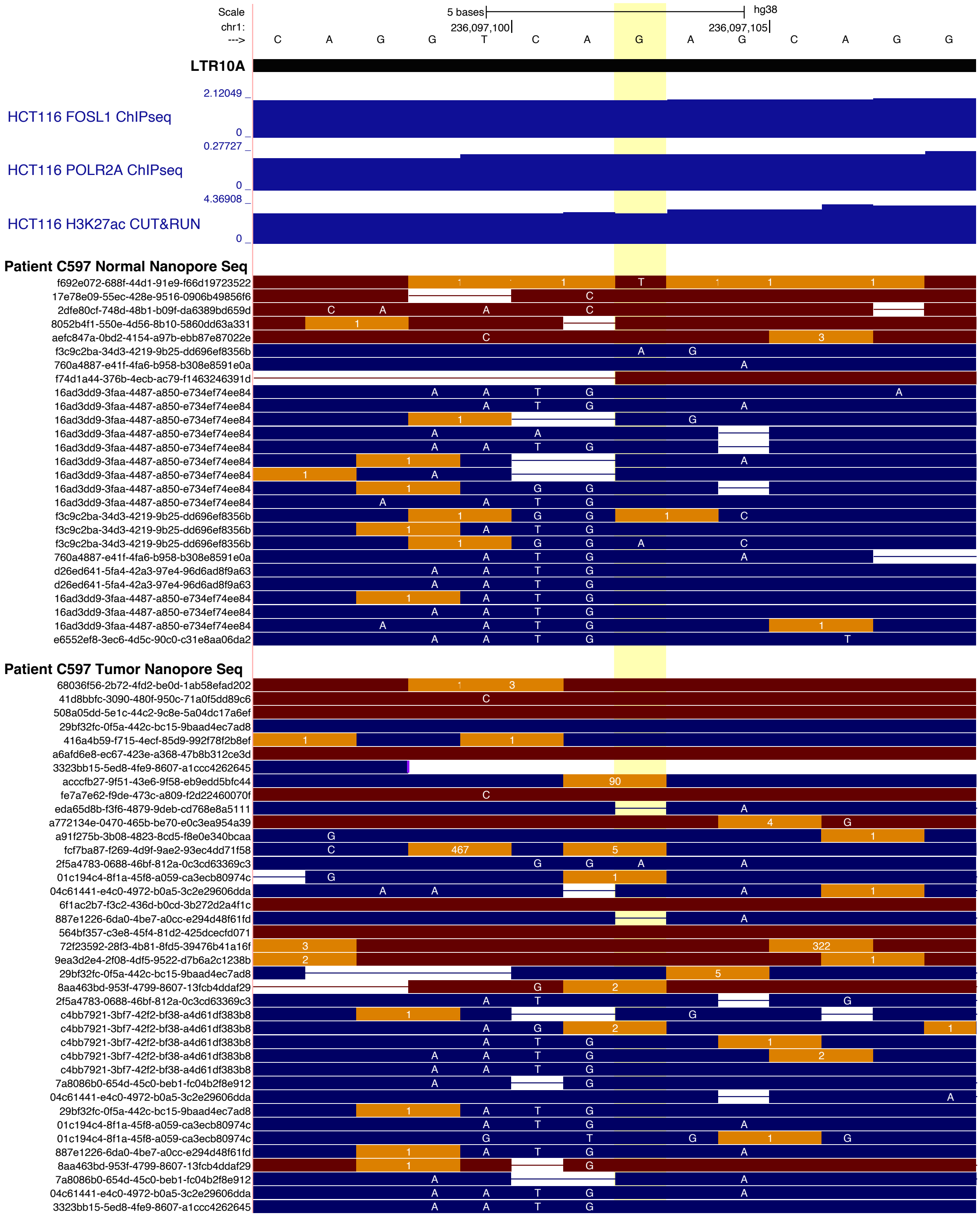

Fig. S80. Genome browser screenshot of a tumor-specific LTR10A VNTR expansion on chr1. Matched tumor/normal reads from patient C597 are derived from a long-read nanopore sequencing dataset of 20 patients with colorectal adenocarcinomas *(80)*. The yellow highlight shows the approximate location of the predicted tumor-specific mosaic expansion, as called by Sniffles2 *(81)*. Orange boxes indicate insertions and numbers within orange boxes indicate insertion sequence lengths (in bp).

Fig. S81.

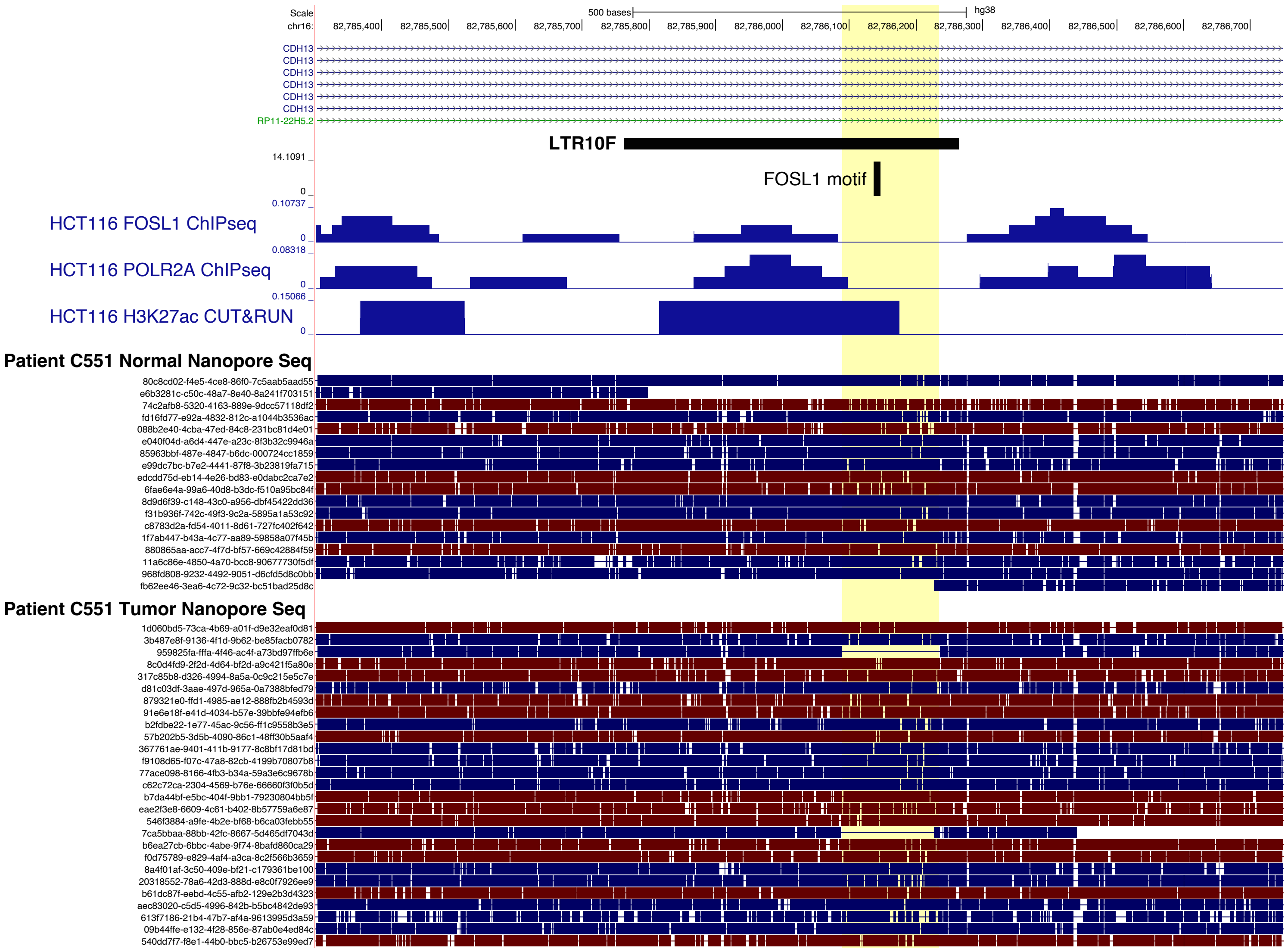

Fig. S81. Genome browser screenshot of a tumor-specific LTR10F deletion on chr16. Matched tumor/normal reads from patient C551 are derived from a long-read nanopore sequencing dataset of 20 patients with colorectal adenocarcinomas *(80)*. The yellow highlight shows the location of the predicted tumor-specific mosaic deletion, as called by Sniffles2 *(81)*.

Fig. S82.

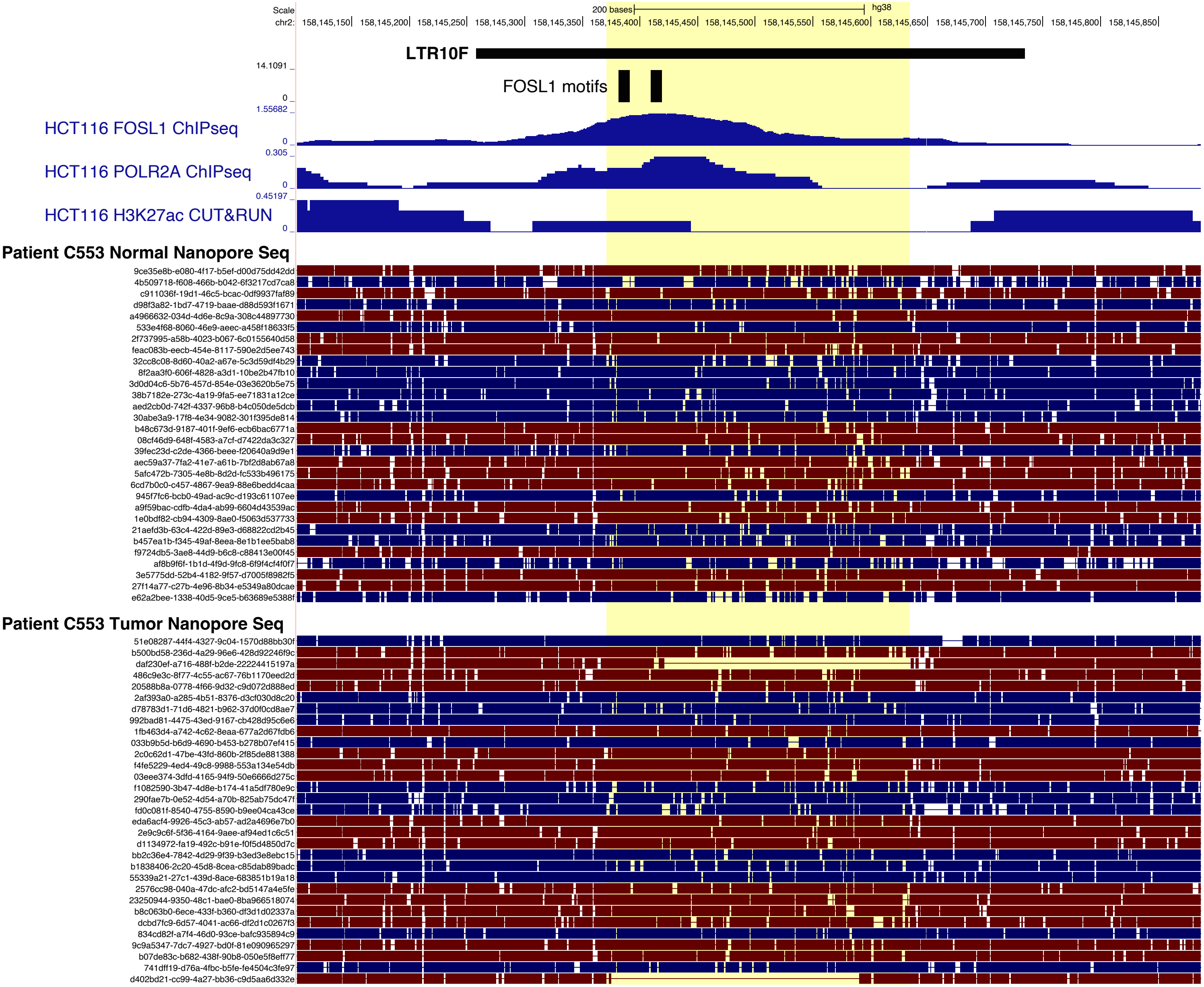

Fig. S82. Genome browser screenshot of a tumor-specific LTR10F deletion on chr2. Matched tumor/normal reads from patient C553 are derived from a long-read nanopore sequencing dataset of 20 patients with colorectal adenocarcinomas *(80)*. The yellow highlight shows the approximate location of the predicted tumor-specific mosaic deletion, as called by Sniffles2 *(81)*.

Fig. S83.

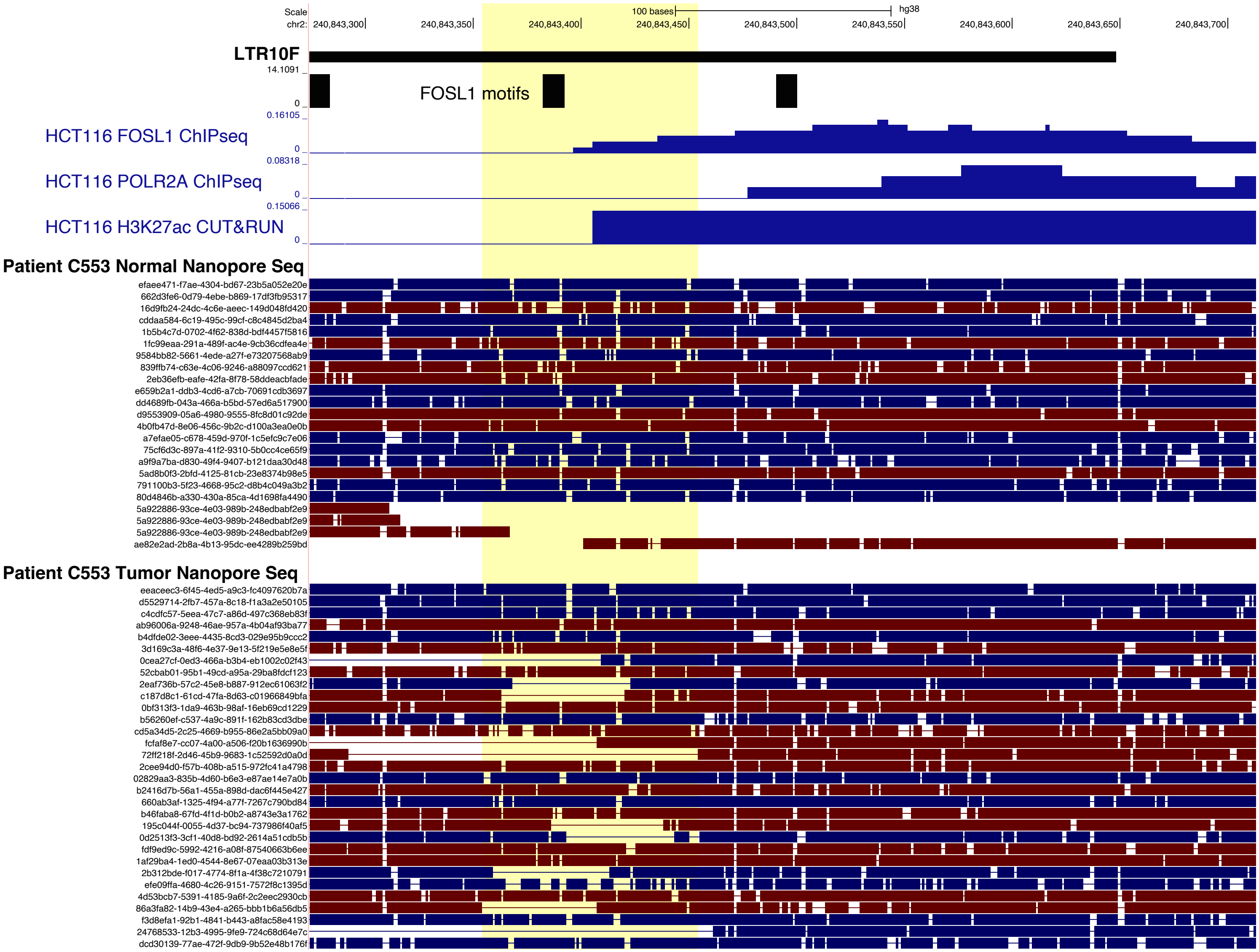

Fig. S83. Genome browser screenshot of a tumor-specific LTR10F deletion on chr2. Matched tumor/normal reads from patient C553 are derived from a long-read nanopore sequencing dataset of 20 patients with colorectal adenocarcinomas *(80)*. The yellow highlight shows the approximate location of the predicted tumor-specific mosaic deletion, as called by Sniffles2 *(81)*.

Fig. S84.

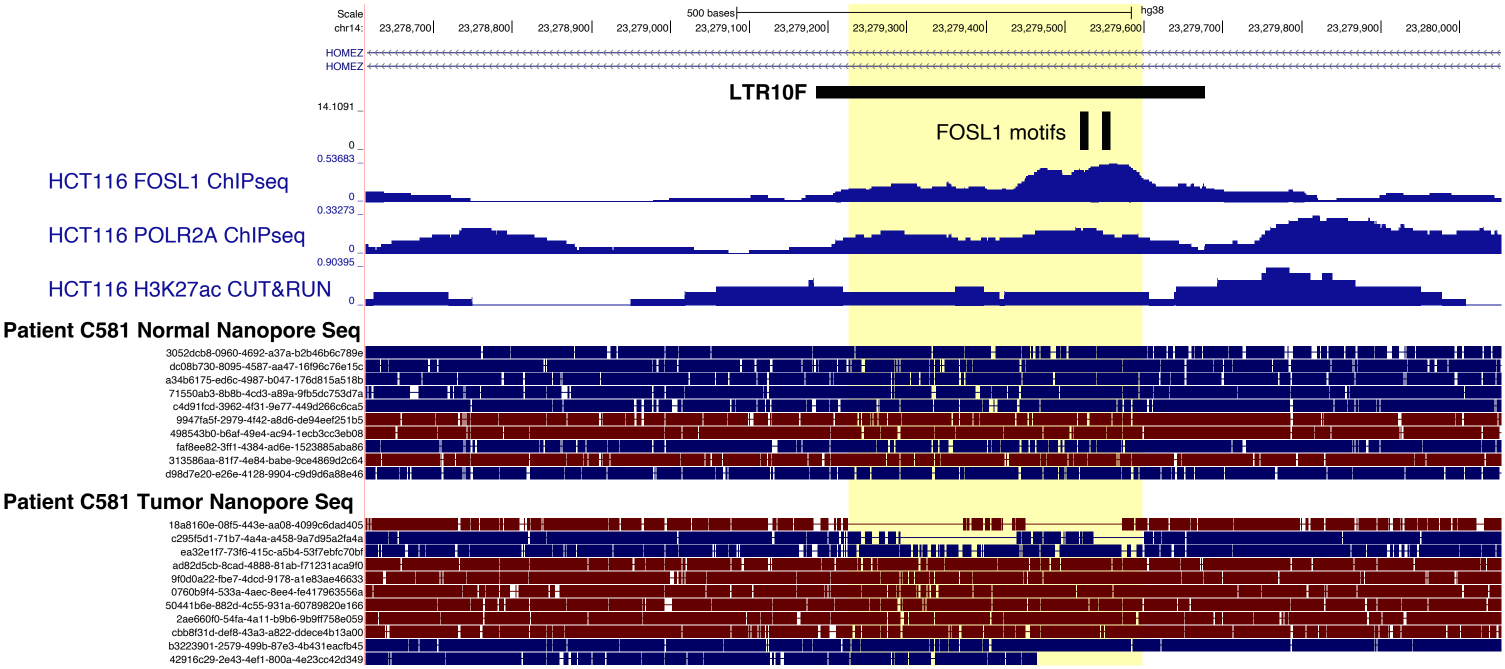

Fig. S84. Genome browser screenshot of a tumor-specific LTR10F deletion on chr14. Matched tumor/normal reads from patient C581 are derived from a long-read nanopore sequencing dataset of 20 patients with colorectal adenocarcinomas *(80)*. The yellow highlight shows the approximate location of the predicted tumor-specific mosaic deletion, as called by Sniffles2 *(81)*.

Legends for tables S1 to S27

Table S1. Enrichment scores for TEs in tumor accessible chromatin from TCGA.

Table S2. Colorectal cancer (CRC) cell lines with mutation status in CRC critical genes, with links to public datasets of H3K27ac ChIP-seq for each cell line.

Table S3. Predicted LTR10-derived promoters in the human genome.

Table S4. Enrichment scores for LTR10A/F elements in regulatory regions defined by Roadmap.

Table S5. Enrichment scores for LTR10A/F elements in human transcription factor datasets generated by Cistrome DB.

Table S6. Enrichment scores for KRAB zinc finger proteins ZNF562 and ZNF671 ChIP-seq peaks in TE regions belonging to different TE subfamilies.

Table S7. DEseq2 results for matched tumor/normal bulk RNA-seq samples from 38 colorectal cancer patients from TCGA.

Table S8. Wilcoxon rank-sum test results for *FOSL1*, LTR10A and LTR10F in tumor vs normal epithelia. Single cell RNA-seq samples from 36 colorectal cancer patients obtained from *(40)*.

Table S9. DEseq2 results for 358 tumor bulk RNA-seq samples from 358 colorectal cancer patients with *KRAS* mutation status from TCGA.

Table S10. Results from univariate and multivariate Cox regression survival analyses.

Table S11. DEseq2 results for trametinib-treated HCT116 cells and HCT116 cell line-based xenografts.

Table S12. DEseq2 results for CRISPR silencing of AP1 component *FOSL1*.

Table S13. DEseq2 results for HCT116 cells treated with 24 hr cobimetinib, 24 hr TNF-alpha, or untreated.

Table S14. Predicted LTR10 enhancers that regulate AP1/MAPK target genes.

Table S15. DEseq2 results for CRISPR silencing of LTR10.ATG12 enhancer.

Table S16. DEseq2 results for CRISPR silencing of LTR10.XRCC4 enhancer.

Table S17. DEseq2 results for CRISPR silencing of LTR10.MEF2D enhancer.

Table S18. DEseq2 results for CRISPR silencing of LTR10.FGF2 enhancer.

Table S19. DEseq2 results for CRISPR silencing of LTR10.MCPH1 enhancer.

Table S20. DEseq2 results for CRISPR deletion of LTR10.KDM6A enhancer.

Table S21. Top six predicted sgRNA for targeting all LTR10A/F elements in the hg38 human genome, designed using CRISPR-TE and CRISPOR.

Table S22. DEseq2 results for CRISPR silencing of *ATG12* transcription start site.

Table S23. Mouse xenograft results showing how the deletion of LTR10.XRCC4 affects tumor response to irradiation.

Table S24. Fraction of H3K72ac and AP1 peaks in HCT116 cells that are derived from LTR10A/F elements.

Table S25. GTEx cis-eQTLs associated with LTR10 VNTR regions.

Table S26. Tumor-specific LTR10 VNTR expansions and contractions from long-read matched tumor/normal whole-genome sequencing from *(80)*.

Table S27. Primers, gRNAs and LTR10 sequences used for CRISPR experiments and functional validation.
